# Modulation of Stem Cells as Therapeutics for Severe Mental Disorders and Cognitive Impairments

**DOI:** 10.3389/fpsyt.2020.00080

**Published:** 2020-04-30

**Authors:** Yongbo Zhang, Yingying Zhao, Xiaopeng Song, Hua Luo, Jinmei Sun, Chunyu Han, Xiaohuan Gu, Jun Li, Guilan Cai, Yanbing Zhu, Zhandong Liu, Ling Wei, Zheng Zachory Wei

**Affiliations:** ^1^Department of Neurology, Beijing Friendship Hospital, Capital Medical University, Beijing, China; ^2^Experimental and Translational Research Center, Beijing Friendship Hospital, Capital Medical University, Beijing, China; ^3^Department of Anesthesiology, Emory University School of Medicine, Atlanta, GA, United States; ^4^McLean Imaging Center, McLean Hospital, Harvard Medical School, Belmont, MA, United States; ^5^Emory Critical Care Center, Department of Surgery, Emory University School of Medicine, Atlanta, GA, United States; ^6^Department of Biological Psychiatry, Peking University Sixth Hospital, Beijing, China; ^7^Department of Biological Psychiatry, Peking University Institute of Mental Health, Beijing, China; ^8^Department of Biological Psychiatry, NHC Key Laboratory of Mental Health (Peking University), Beijing, China; ^9^Department of Biological Psychiatry, National Clinical Research Center for Mental Disorders, Beijing, China; ^10^Department of Neurology, Emory University School of Medicine, Atlanta, GA, United States

**Keywords:** stem cell therapy, severe mental illnesses, calcium signaling, diagnosis, regeneration, function recovery

## Abstract

Severe mental illnesses (SMI) such as schizophrenia and bipolar disorder affect 2-4% of the world population. Current medications and diagnostic methods for mental illnesses are not satisfying. In animal studies, stem cell therapy is promising for some neuropsychiatric disorders and cognitive/social deficits, not only treating during development (targeting modulation and balancing) but also following neurodegeneration (cell replacement and regenerating support). We believe that novel interventions such as modulation of particular cell populations to develop cell-based treatment can improve cognitive and social functions in SMI. With pathological synaptic/myelin damage, oligodendrocytes seem to play a role. In this review, we have summarized oligodendrogenesis mechanisms and some related calcium signals in neural cells and stem/progenitor cells. The related benefits from endogenous stem/progenitor cells within the brain and exogenous stem cells, including multipotent mesenchymal-derived stromal cells (MSC), fetal neural stem cells (NSC), pluripotent stem cells (PSC), and differentiated progenitors, are discussed. These also include stimulating mechanisms of oligodendrocyte proliferation, maturation, and myelination, responsive to the regenerative effects by both endogenous stem cells and transplanted cells. Among the mechanisms, calcium signaling regulates the neuronal/glial progenitor cell (NPC/GPC)/oligodendrocyte precursor cell (OPC) proliferation, migration, and differentiation, dendrite development, and synaptic plasticity, which are involved in many neuropsychiatric diseases in human. On the basis of numerous protein annotation and protein-protein interaction databases, a total of 119 calcium-dependent/activated proteins that are related to neuropsychiatry in human are summarized in this investigation. One of the advanced methods, the calcium/cation-channel-optogenetics-based stimulation of stem cells and transplanted cells, can take advantage of calcium signaling regulations. Intranasal-to-brain delivery of drugs and stem cells or local delivery with the guidance of brain imaging techniques may provide a unique new approach for treating psychiatric disorders. It is also expected that preconditioning stem cell therapy following precise brain imaging as pathological confirmation has high potential if translated to cell clinic use. Generally, modulable cell transplantation followed by stimulations should provide paracrine protection, synaptic modulation, and myelin repair for the brain in SMI.

## Introduction

Severe mental illnesses (SMI) or psychotic disorders, referring to bipolar disorder (BD), major depressive disorder (MDD), and schizophrenia and schizoaffective disorder, cause long-duration and severe disability at different ages. Among them, schizophrenia and BD affect 2-4% of the world population. Current medications have not shown significant improvement in these diseases (1, 2). Researchers have sought to identify a clinically relevant brain pathology of schizophrenia or bipolar disorder behind the persistent psychosis condition, which still appears to be unknown (3). There seem to be, although controversial, psychological and physiological over-excitabilities, or immunological hyper-body, as well as hyper-active, neurons and hyper-connectivity of the excitatory neuronal network in certain brain regions (hyper-brain). Recent advances indicate that abnormal myelination can occur in schizophrenia patients, such that using brain imaging can detect abnormalities of the white matter (WM), and that electro- and magnetoencephalography (EEG/MEG) and functional magnetic resonance imaging (fMRI) show neuronal dysconnectivity (4). These changes in myelination, excitability, and functional connectivity can be caused by genetic and epigenetic transcriptional dysregulation, triggering and largely relying upon persistent Ca^2+^ signaling modulation (5–7). To date, there are challenges for animal models of the development of severe mental illnesses. Schizophrenia and bipolar disorder, among the most common severe conditions, both show significant decreases in occupational, social, and/or personal functioning. Based on symptoms, schizophrenia diagnosis usually requires hallucinations or delusions, although the patients also develop abnormal psychomotor behavior, cognitive deficits, sensory gating dysfunction, and negative symptoms (months). Bipolar disorder has a single manic episode (weeks), one or several hypomanic episodes, and major depressive episodes, which can be accompanied by psychosis (days to weeks).

Multipotent mesenchymal-derived stromal cells (MSC) and lineage-restricted neuronal/glial progenitor cells (NPC/GPC), oligodendrocyte precursor cells (OPC), and immature neurons differentiated from types of pluripotent stem cells (PSC) have all been considered appropriate cell sources for transplant into various animal models with neuropsychiatric pathology and cognitive impairments (8–11). Transplantation of lineage-committed, fully differentiated cells has been considered to provide competent cell sources, especially for CNS repair, in which a particular type of neuron or glia may be needed. Examples may include an inhibitory preceding neuron or an input from local balances in pre-synaptic inhibitory neurotransmitters (targeting the disturbed connectivity). Fundamental stem cell research that allows the generation of a pure and differentiated specific type of cell is still needed. Despite this, certain cell types have been tested in various neurological (mild-to-moderate and severe) and mild-cognitive-impairment/neuropsychological models. The models are, for example, BD, Huntington's disease (HD), MDD, neurotrauma, Parkinson's disease (PD), schizophrenia and schizoaffective disorder, and stroke (12, 13). The types of cells that have recently been focused on have included human and rat bone marrow MSC (BMSC), human immature neurons, and human and mouse NPC. Among them, oligodendrocyte, from either transplanted cells or endogenous progenitors, is capable of playing a dominant supporting/modulatory role in neuronal activity at axons (14). Considering that, in patients with schizophrenia, there are some deficits in brain myelination and expression of myelin-related genes, or there are oligodendrocyte abnormalities in the brain (15), correction of the oligodendrocyte phenotypes as well as neurotrophic supports *via* transplantation could be considered in treating the psychotic disorder.

Ca^2+^ signals mediate both myelin damage (pathophysiological Ca^2+^ overload) and re-myelination repairs (physiological Ca^2+^ activity). Oligodendrocyte injury has been identified as a chronic detrimental change in the brain, with neurodevelopmental and neurodegeneration problems. Ca^2+^ influx and signaling play multiple roles in the oligodendrocyte biology through different Ca^2+^ channels (16). Expression of these channels is symphonized in the oligodendrocytes and OPC, although many of them have been reported to be expressed in neurons as well. They are subtypes of (1) α-amino-3-hydroxy-5-methyl-4-isoxazolepropionic acid (AMPA) receptor (AMPAR), (2) γ-aminobutyric acid (GABA) receptor (GABAR), (3) glycine receptor (GlyR), (4) inositol trisphosphate receptor (IP3R), (5) muscarinic acetylcholine receptor (mAChR), (6) metabotropic glutamate receptor (mGluR), (7) sodium-calcium exchanger (NCX), (8) N-methyl-D-aspartate (NMDA) receptor (NMDAR), (9) P2X/P2Y purinoreceptor, (10) ryanodine receptor (RyR), (11) serotonin receptor (or 5-hydroxytryptamine receptor, 5-HTR), and (12) L-type Ca^2+^ currents-generating voltage-gated calcium (Ca_v1_) channel (or L-type voltage-operated Ca^2+^ channel, L-VOCC), among a total of four types (L, N, P, and T-types) of VOCCs essential in activity-dependent release out from endocrine cells and neurons.

This review is primarily focused on stem cell therapy and is evidence-based in identifying promising therapeutic treatments against some neuropsychiatric symptoms and cognitive/social deficits. In order to overcome neurodevelopmental disorders and neurodegeneration by using stem cells, we discuss their ability demonstrated in stem cell transplantation not only to recover the endogenous neurogenic niche during neural development/regeneration but also to re-initiate oligodendrogenesis, synaptogenesis, and myelinogenesis (17). Additionally, through the action of the cellular and molecular mechanisms, transplanted stem cells and their derived cells secrete autocrine and paracrine factors (e.g., oxytocin), preventing neurocognitive impairments. Furthermore, a strategy of stimulating or preconditioning stem cells may provide additional benefits, such as further increasing secretions or expressions of the autocrine and paracrine factors as well as evoking activity-dependent responses and repairs, many of which show physiological Ca^2+^ signal activation and are regulated by Ca^2+^-activated proteins. The review will then focus on these mechanisms in stem cell therapy for neuropsychiatric and psychological disorders.

## Oligodendrogenesis, Calcium Signal, and Myelination

Post-natal oligodendrogenesis and myelinogenesis, orgininated by OPC from the parenchyma and subventricular zone (SVZ) regions, are regulated in an activity-dependent manner and in response to injury stimulus. Many morphogens, growth factors (e.g., BDNF, FGF2, NGF, NT-3, and PDGF), and extracellular matrix elements have been identified as important for oligodendrocyte identity, differentiation, and functionality. In addition, SVZ neural stem cells (NSC) and NSC-derived transit-amplifying progenitor (TAP) cells further give rise to many precursor cells, such as PDGFRα-positive and NG2-positive OPC and DCX-positive neuroblast for gliogenesis and neurogenesis. Recent investigations using exogenous OPC transplantation for spinal cord injury have proved the concept of providing myelination and neuronal protection following engraftment (18).

Accumulating data have supported the hypothesis that Ca^2+^ signaling in preceding neurons, myelin, and OPC plays a role in the translation of the neuronal activity to oligodendrocyte differentiation and myelination (19). Stimulation of endogenous and transplanted NPC/OPC, activating physiological Ca^2+^ signals, is likely to enhance the myelination and modulate neuronal activity. The stimulation may therefore facilitate re-myelination repairs during development or following inflammatory/neurodegeneration-related myelin sheath and oligodendrocyte damages. An early response to regenerative stimulus following injury occurs when the differentiated oligodendrocytes re-form the myelin sheath around demyelinated axons (20). Among the regenerative stimuli, several Ca^2+^-binding and Ca^2+^-dependent factors have been reported to control the temporary maintenance, proliferation, and migration of OPC, beneficial for oligodendrogenesis and re-myelination (**Data Sheet 1**). The most studied Ca^2+^-binding proteins in neurons and glias are calcium-binding protein 4 (CABP4), voltage-gated calcium channel subunit alpha Ca_v2.1_, which is from the calcium channel alpha-1 subunit family (CACNA1A), and Ca_v2.2_ (CACNA1B), calmodulin family proteins including calmodulin-1 (CALM1), CALM2, and CALM3, calcineurin B homologous protein 1, which is from the calcineurin regulatory subunit family/CHP subfamily (CHP1), calsenilin, which is from the recoverin family (KCNIP3), and S-100 family proteins including S100A1, calvasculin (S100A4), and migration inhibitory factor-related protein 14 (S100A9). The current review summarizes these genes and some other Ca^2+^-dependent/activated proteins that are expressed in neurons, oligodendrocytes, and some NSC/NPC. There are long lists of candidate genes that are potentially affected by the hyper-excitability or abnormal neuro-connectivity and Ca^2+^ burden increase leading to myelin damage and neuro-toxicity; on the other hand, regenerative events and artificial modulation of physiological Ca^2+^ signals would re-inventory the endogenous stem cell pool as well as the capacity of exogenous cells. We sought to identify this capacity in neuronal replacement, trophic supports, and myelin repair for stem cell therapy by taking advantage of the Ca^2+^ signaling mechanisms in neurons and stem/progenitor cells.

During cortical development, intracellular Ca^2+^ activity coupling with downstream signaling pathways can be regulated by cell membrane-localized GABA receptors, gap junctions, ionotropic glutamate receptors (iGluRs), and mGluRs. Upon membrane depolarizations in cells, L-VOCCs are major players in Ca^2+^ influx [Ca^2+^]_i_. All over the body, L-type Ca^2+^ currents have been shown to be responsible for the excitation-secretion coupling of endocrine cells (for aldosterone secretion from the adrenal cortex), cardiomyocyte, and neurons (for transmitter release). In the brain, voltage-gated calcium channel subunit alpha 1C (CACNA1C), Ca_v1.2_, and CACNA1D, Ca_v1.3_ (common variants in CACNA1C and CACNA1D identified with BD, MDD, or schizophrenia), are the predominant L-type Ca_v_ channels and are expressed in various cell types including NPC. Regulation and control of the intracellular Ca^2+^ activity and Ca^2+^-dependent mechanisms during cortical and WM development are closely connected with the wingless/int-1 (Wnt) signaling pathway, Sonic hedgehog (Shh), and S-100 family proteins, which can be activated in both neurons and glias.

β-catenin (human gene name: *CTNNB1*), one of the Ca^2+^-dependent gene regulators and one of the most conserved genes across vertebrates, is the central counterpart in the canonical Wnt/β-catenin pathway. β-catenin has been associated with a variety of diseases ranging across dilated cardiomyopathy, fetal alcohol syndrome, primary hepatocellular carcinoma, colorectal cancer, ovarian carcinoma, breast cancer, lung cancer, and glioblastoma, and has been considered in the pathogenesis of common oral lesions in oral and maxillofacial region-oral potentially malignant disorders and their progression to oral squamous cell carcinoma, salivary gland tumors, and odontogenic tumors. *CTNNB1* variants have been reported in schizophrenia (21). To induce an activation of the canonical Wnt signaling, unbound cytoplasmic β-catenin in complex must be translocated to the nucleus. Transcriptional co‐activators of β-catenin may include lymphoid enhancer-binding factor 1 (LEF-1), T-cell-specific transcription factor 1 (TCF-1, human gene name: *TCF7*), and some other HMG-box DNA-binding proteins such as TCF-3 (human gene name: *TCF7L1*) and TCF-4 (human gene name: *TCF7L2*). Wnt proteins that activate β‐catenin have included: Wnt-1, Wnt-2, Wnt-2b, Wnt-3, Wnt-3a, Wnt-7a, Wnt-7b, Wnt-8, and Wnt-8c. *WNT2B* and *WNT7A* variants have been reported in BD (22). One of the schizoaffective disorder-related genes, disrupted in schizophrenia 1 (DISC1), and its regulator DIX domain containing 1 (DIXDC1) have been shown to positively regulate Wnt/β‐catenin signaling in human NPC and FoxG1^+^/Tbr2^+^-positive neurons. The study identifies and supports some significant genes after DISC1 disruption, including *ASCL1*, *DBX2*, *DLX1*, *DLX2*, *GSX2*, *MSX2*, *NEUROG2*, *PAX3*, *PAX7*, and *WNT8B* (in human gene names) (23). Prostaglandin E2 (PGE2) signaling, another crosstalk signaling of Wnt/β‐catenin, further transcribes some SMI-related genes, B-cell lymphoma 1 (Bcl-1, human gene name: *CCND1*), matrix metalloproteinase (MMP)-9 (human gene name: *MMP9*), and cyclooxygenase-2 (COX-2, human gene name: *PTGS2*). In distinct animal models, intranasal delivery of recombinant Wnt-3a proteins showed neuroprotective effects through activating the Wnt/β‐catenin pathway (24). It also promoted endogenous regenerative responses (such as neuroblast migration) in mouse models of ischemic stroke and traumatic brain injury (25). These effects could be blocked by a Wnt inhibitor of either Dickkopf related protein 1 (Dkk-1) or XAV-939. Wnt-3a delivery increased expressions of BDNF, Forkhead box protein M1 (FOXM1), GDNF, and VEGF (26, 27).

Hedgehog family Shh serves as a morphogenic protein during early neuronal development and is involved in the oligodendrogenesis of developing brain and spinal cord. In differentiating neurons, Shh inhibits Gli transcription factors under the control of electrical activity and Ca^2+^-mediated responses. Shh also regulates the oligodendrocyte basic helix-loop-helix (bHLH) transcriptional factors Olig1/2/3 (human gene name: *OLIG1*, *OLIG2*, *OLIG3*) in NPC/OPC and neurons. Shh initiates developmental signals through binding to the Brother of the cell-adhesion-molecule-related/downregulated by oncogenes (CDO) (BOC; human gene name: *BOC*), CDO (human gene name: *CDON*), Growth arrest-specific protein 1 (GAS-1; human gene name: *GAS1*), Protein patched homolog 1 (Ptch1; human gene name: *PTCH1*), and Ptch2 (human gene name: *PTCH2*) receptor/co-receptors in vertebrates. Mutating BOC gene, a co-receptor of the Shh signaling pathway, shows delayed myelination and less PDGFRα-positive OPC during development and reduced myelin basic protein (MBP) production in mice (28). Inactivation of Shh signaling by inhibiting downstream Smoothened (Smo) causes a dose-dependent decrease in MBP and myelin-associated glycoprotein (MAG) in differentiating oligodendrocytes, which impairs the myelin formation and repair. Interestingly, Shh-overexpressing BMSC showed increased Shh signaling, basic fibroblast growth factor (bFGF, human gene name: *FGF2*), and VEGF in an SCI model of rats (29).

S-100 family proteins, such as S-100A1, S-100A4, and S-100A9, are also Ca^2+^-dependent and functionally conserved across vertebrates. S-100 family proteins have been involved in the regulation of intracellular Ca^2+^ homeostasis, cell proliferation, migration, differentiation, cell survival, and apoptosis. There is evidence that an extracellular S-100A4 or S-100B can interact with epidermal growth factor (EGF) and bFGF to facilitate the heparin-binding growth factor signaling downstream. EGF and bFGF are reported to promote the proliferation and migration of OPC/NPC (30–32). The extracellular S-100 family proteins have been shown to interact with and respond to almost all types of neural cells, vasculature cells, and inflammatory/immune cells in the brain. High levels of the inflammatory S-100 family proteins can cause myelin damage and cognitive decline (33). Among them, S-100B, primarily essential to oligodendroglial differentiation and maturation, is released during earlier phases of brain injury, increasing excessive inflammatory stimuli and leading to neurodevelopmental problems in the long term. The S-100B/receptor for advanced glycation endproducts (RAGE) axis, normally inhibiting immature NG2-positive OPC from becoming mature MBP-positive oligodendrocyte, will likely be enhanced, impairing oligodendrogenesis and thereby reducing re-myelination. Furthermore, persistent elevations of S-100B level can also induce glial activation, astrogliosis, and NF-κB activation, potentially compromising neuronal/synaptic integrity and synaptogenesis in developing brains after neonatal/perinatal inflammatory conditions (34, 35).

Other Ca^2+^-dependent/regulated proteins expressed in oligodendrocyte may include (**Table 1**): AAA ATPase family proteins (human gene names: *ATAD3A*, *ATAD3B*, *ATAD3C*), cadherin family proteins such as the neural cadherin, CD antigen CD325 (human gene name: *CDH2*), dystroglycan family proteins such as dystroglycan (human gene name: *DAG1*), histone deacetylase family proteins such as the histone deacetylase 1 (human gene name: *HDAC1*), LDLR family proteins such as the low-density lipoprotein receptor-related protein 2 (human gene name: *LRP2*), pleiotrophin family proteins such as neurite outgrowth-promoting factor 2 (human gene name: *MDK*), and tenascin family proteins such as the neural recognition molecule J1-160/180 (human gene name: *TNR*).

**Table 1:** Summary of calcium-dependent/activated proteins that are related to neuropsychiatry in human.

Human Gene	Protein Name (UniProtKB ID)	Family	Potentially Interacted Protein		
			Calcium-dependent gene	SMI-related gene	Other gene
***ADCY8***	Ca^2+^/calmodulin-activated adenylyl cyclase (P40145)	Adenylyl cyclase class 4/guanylyl cyclase family	*CALM1,CALM2*	*CACNA1C,DRD1,DRD5*	*ADRB2,AKAP5,CAV3,CFTR,GNAI1,GNAL,GNAS,GNAZ,GPR83,GRM3,KRAS,PPP2CA,PPP2R1A,PRKACA,PRKACB,PRKACG,PRKAR1B,PRKAR2B,RAPGEF3,RAPGEF4*
***APEX1***	DNA-(apurinic or apyrimidinic site) lyase (P27695)	DNA repair enzymes AP/ExoA family	*HMGB1,LRRK2*	*APP*	*ABCE1,AGR2,ANP32A,ARF6,ARIH2,ASCL2,ATM,BRCA1,BRCA2,BUB3,CAPNS1,CCDC124,CCNA1,CFAP74,CGGBP1,CITED1,CLVS2,DDB1,DOT1L,EED,EEF1A1,EEF2,EFTUD2,EGFR,EIF1B,EIF6,ELOC,EP300,ESR2,FBXO7,FCGR2A,FEN1,GADD45A,GZMA,GZMK,G3BP1,HDAC1,HDAC2,HDAC3,HIF1A,HLA-B,HLF,HMGA1,HMGA2,HNRNPL,HNRNPK,HNRNPR,HNRNPUL1,HOXC13,HSPA1A,HSPA1B,ILF2,ILF3,JUN,KDM1A,KLHL36,KPNA3,KRT8,LGALS1,LIG1,MAPT,MCC,MCL1,MCM2,MCM3,MCM4,MCM5,MCM6,MCM7,MDM2,METTL1,METTL14,Morf4L1,Morf4L2,MRE11,MUTYH,NAE1,NPM1,NPY2R,NTHL1,NTRK1,NUDT3,NXF1,OGG1,ORC2,OTUB1,OTUD6B,PABPC1,PAK2,PAPSS2,PARP1,PCNA,PDE12,PLCB1,POLB,POLR3D,PRDX6,PRKDC,PRKN,PRPF19,PSMG1,RAD50,RIC8A,RNF4,RPA1,RPA2,RPA3,RPSA,RUVBL1,RUVBL2,SDHA,SET,SFPQ,SIN3A,SIRT1,SIRT6,SNRPD1,SRPK1,SRPK2,STAT3,SYNCRIP,TCF21,TCP1,TDG,TERF1,TERF2,TERF2IP,TFAP4,TP53,TRAF2,TRAF6,TWF2,TXN,TXNRD1,KIAA1429,JUP,UBE2I,UBR3,UNG,WDR77,XPOT,XRCC1,XRCC5,YBX1,YWHAE,ZFR2*
***ASTN1***	Neuronal migration protein GC14 (O14525)	Astrotactin family	*GRM7,MYO10*	*CDKL5,GRM7,MAP6*	*AP4E1,BRINP1,BRINP2,CNTN2,HSPA9,LAMP1,NXPH1,PIANP,PKIA,YWHAB,YWHAE*
***ATP2B2***	Plasma membrane calcium-transporting ATPase 2 (Q01814)	Cation transport ATPase (P-type) family, Type IIB subfamily	*CABP4,CALM1,CALM2,CALM3,CDH23,DLG4,ITPR1,LRRK2,SNTA1*	*APP,ATP6V1A,C4A,DLG4,GPR89A,PRNP,SLC8A2*	*ABCB9,ABCC4,ABCC10,ABCD1,ABCD3,ABCD4,ADAM7,ADAMTS4,ALG8,ALG10,APOD,ARL6IP1,ATG9A,ATP2B1,ATP2B3,ATP2B4,ATP9A,AZGP1,BCAP31,BTBD9,CALCA,CCR6,CD79B,CD83,CERS6,CHSY1,CLU,CMKLR1,CPT1A,CYP2B6,C12orf49,C17orf78,DGAT1,DGUOK,DHRS7,DHRS9,DLG1,DLG2,DLG3,DNAAF2,DPH6,EDNRB,EFNB2,EMC8,ENTPD7,ERBIN,ERMP1,ESPN,ESYT2,FAM174A,FAR2,FPR2,FZD2,FZD7,FZD10,GALNT6,GGH,GHDC,GINM1,GLB1L2,GOLGA5,GOLIM4,GOLM1,GPAM,GPR161,HIDE1,HLA-DQA1,HLA-DRA,HSD17B1,HSD17B6,HTR2C,HTR3A,IFNL1,IFNLR1,IL1R2,ITM2B,KLRD1,LAGE3,LCLAT1,LEMD2,LGALS8,LGR4,LILRB4,LPAR4,LPCAT2,MAOB,MPC2,MUCL1,MYH15,NRM,NUFIP1,OC90,OPALIN,OSTC,PCDHGA7,PCDHGB1,PCNX3,PIBF1,PIGN,PIGO,PPP1R9B,PRKACA,PRKACB,PRKG1,PRKG2,PTGFR,PTGIR,PTPRQ,RAPGEF5,RHOT2,RXFP1,RXYLT1,SCARA3,SCRIB,SDF4,SLAMF1,SLAMF9,SLC2A5,SLC4A2,SLC4A7,SLC9A3R2,SLC15A4,SLC22A16,SLC26A6,SLC38A9,SLC45A2,SLC47A1,SPACA1,SQLE,SRPRB,STIM1,ST3GAL1,STEAP3,TAS2R7,TIMM23,TMEM63A,TMEM63B,TMEM134,TMEM168,TNPO3,TOR1A,TPST2,TRABD,TSPAN14,UBC,UBXN8,UXS1,VAC14,VAV1,VSIG4,YWHAB,YWHAE,YWHAZ*
***CABP4***	Calcium-binding protein 4 (P57796)	–	–	–	*AIPL1,ASPM,BCL6,CACNA1D,CACNA1E,CACNA1F,CACNA2D4,DCAF6,GPR179,GRM6,IKZF3,IQCB1,LNX2,LRIT3,MFHAS1,MYH10,NYX,OBSCN,RD3,SLC24A1,SPATA7,SPTB,TLE5*
***CACNA1A***	Voltage-gated calcium channel subunit alpha Ca_v2.1_ (O00555)	Calcium channel alpha-1 subunit family, CACNA1A subfamily	*CACNA1B,CALM1,CALM2,CALM3,CAMK2A,KCNMA1,PRKCG,SYT1*	*CACNA1C*	*ABCA2,ABI1,ACTN1,ADGRL1,AGRN,ALDOA,AMIGO2,AP2M1,ARHGAP22,ASNA1,BCL6,BTG3,CABP1,CACNA1E,CACNA1S,CACNA2D1,CACNA2D2,CACNA2D3,CACNB1,CACNB2,CACNB3,CACNB4,CACNG2,CATSPER1,CATSPER4,CKAP5,CRIM1,CRMP1,CSNK2B,CYSRT1,C1QTNF1,DNAJB5,EFEMP1,EFEMP2,EHMT2,EIF3A,FBLN1,FSCN1,GNAQ,GNB1,GNB2,GNG2,GOLGA6L9,GRN,HECW1,HHATL,HIVEP1,HSPG2,IP6K1,JAG2,KALRN,KDM5B,KHDRBS3,KIAA1191,KLHL1,LAMB1,LLGL1,LRP1,LTBP1,LTBP3,LTBP4,MANBAL,MAP1B,MATK,MATN2,MEGF6,MEGF8,MIA3,MOAP1,NDUFB8,NELFCD,NELL1,NELL2,NOTCH1,NOXA1,OLIG1,PCSK5,PCSK6,PMM1,PPIG,PPM1A,PPP1R12C,PTGDS,PUF60,RBM12B,REV3L,RHOA,RIMBP2,RIMS1,RNF138,RPL31,RPS17,SCP2,SPRY1,SRRM4,SRSF1,STAC3,SUMF2,TAF15,TCTN2,TCTN3,TELO2,TNIK,TSC22D1,TSPAN7,TSPOAP1,TUBB2B,UBE2L3,UQCRC2,VARS1,VPS52,VWF,WBP1,YLPM1,YWHAB,ZCCHC17,ZNF233*
***CACNA1B***	Voltage-gated calcium channel subunit alpha Ca_v2.2_ (Q00975)	Calcium channel alpha-1 subunit family, CACNA1B subfamily	*CACNA1A,KCNMA1,SYT1,SYT4*	–	*APBA1,APBA2,CACNA1E,CACNA2D1,CACNA2D2,CACNA2D3,CACNA2D4,CACNB1,CACNB2,CACNB3,CACNB4,CACNG2,CACNG3,CASK,CATSPERB,CATSPERD,DPYSL2,DYNC1I1,DYNLT1,GNAO1,GNB2,GNG5,MAP1B,MAP1LC3B,MRFAP1,MTNR1A,MTNR1B,PDLIM5,PPM1A,RGS12,RIMS1,RIMBP2,RXRB,SYT3,UBE2L3,YWHAB*
***CACYBP***	Calcyclin/S-100A6-binding protein/Siah-1-interacting protein (Q9HB71)	Target protein of S-100 family	*CTNNB1,PRKCA,RAC3,S100A1*	*APC,CTNNB1,ERBB2,HDAC4,RPTOR*	*AAR2,AARS1,ACO2,AGR2,AHSA1,AIM2,ANXA2,AP3D1,ARF6,ATG16L1,AXIN1,BAG2,BEX3,CARM1,CCR2,CD8A,CEP104,CEP192,CFLAR,CNOT8,COPG1,CPT1A,CRAT,CYLD,C20orf194,DDX11,DERL1,DET1,DLD,DNM3,DUSP13,EED,EFTUD2,EGLN3,EIF1B,EIF2AK4,EIF6,EPB41,ERCC2,EWSR1,EYA3,FBH1,FBXO7,FBXO24,FBXW2,FERMT1,FKBP4,FKBP5,FKBP8,FN1,FOS,FRMD5,FTSJ1,GH1,GLP1R,GNAZ,GNA13,GNA15,GSK3A,GSK3B,HDAC6,HIGD1A,HLA-B,HNRNPA0,HSD17B10,HSP90AA1,HSP90AB1,HSP90B1,IFI16,IKBKE,IPO11,IRS4,ITGA4,ITPRIP,JUN,KDM4A,KEAP1,KIAA1429,KLHL1,KLHL38,LRRC6,MAGI1,MAOB,MAPK1,MAPK13,MAPT,MCC,MCM5,MCM7,METTL1,MLF1,MOS,MPG,NAA20,NAA25,NAT10,NDN,NEK8,NFATC2,NLRP12,NME2,NOD1,NPHP1,NPHP4,NTRK1,NUDC,OFD1,OTUB1,OTUD5,PEA15,PGLS,PIH1D1,PINX1,PIWIL1,PIWIL4,PPEF2,PPP6R2,PRDX1,PRDX2,PRKAB1,PTPN3,RAF1,RAG1,RECQL4,REC8,RGS11,RNF41,RPAP3,RPL7A,SIAH1,SIAH2,SIM2,SKP1,SKP2,SLC9A3R2,SMARCD2,SNX4,STIP1,STK3,STXBP2,ST13,SUGT1,S100A6,S100B,S100P,TAGLN2,TBL1X,TET3,TFDP3,TFE3,TNIK,TP53BP1,TPD52,TRAF6,TRIM17,TRIM43,TSSK4,TUBG1,UBE2I,URI1,USP13,USP18,USP19,USP49,VCAM1,WSB2,WWOX,YWHAE,YWHAH,YWHAZ,ZMYND12,ZRANB1*
***CADPS***	Calcium-dependent secretion activator 1 (Q9ULU8)	–	*DNAJC5,LRRK2,NCS1,SNAP25,STX1A,SYT1,UNC13A,VAMP2*	*DISC1,PRNP,SESTD1*	*CLIP1,C17orf67,EXOC1,FAM9B,FAM161A,GABRA3,ITM2B,KIAA1549,LEMD2,LUC7L2,MSL1,NEK4,PIP5K1A,PI4KB,POM121,RASL10B,PRSS8,RASL10B,SDCBP,SHFL,TCL1A,TMEM63B,TTLL10,UNC13B,UNC13C,VSNL1,YWHAE,ZNF572,ZNF688*
***CADPS2***	Calcium-dependent secretion activator 2 (Q86UW7)	–	*UNC13A*	*AUTS2,DYRK1A*	*ANP32E,ARF4,ARF5,ASB6,ASB12,BACE2,BDNF,FAM180A,GOLGA2,INPPL1,MEGF10,NRG3,PIGP,PRDM16,PTPRZ1,RUNDC1,SPAG17,SRPK2,STX6,TEX45,TSPOAP1,UBE2D1,UBE2D2,UBE2D3,UBE2D4,UBE2E1,UBE2E3,UBE2N,UBE2U,UBE2W,VAMP4,WDR91*
***CALM1***	Calmodulin-1 (P0DP23)	Calmodulin family	*CAMK2A*	*CACNA1C*	*CAMKK1,CAMKK2,CAMK1,CAMK2B,CAMK2D,DAPK1,DAPK2,FGF12,KCNN2,MYLK,NOS1,NOS2,NOS3,PPP3CA,RYR2*
***CALM2***	Calmodulin-2 (P0DP24)	Calmodulin family	*CALM1,CAMK2A*	*GRIN1*	*CAMK1,CAMK2D,C11orf65,KCNN2,KCNQ2,MYLK,PPP3CA,RYR2*
***CALM3***	Calmodulin-3 (P0DP25)	Calmodulin family	–	–	*ADAMTS12,APPBP2,CAMTA2,C11orf65,IQCE,IQCN,IQGAP3*
***CAMK2A***	Calcium/calmodulin-dependent protein kinase type II subunit alpha (Q9UQM7)	Protein kinase superfamily, CAMK Ser/Thr protein kinase family, CaMK subfamily	*CACNA1A, CALM1, CALM2, CALM3,DLG4,GRM4,GRM7,PTK2B,SYN1*	*ATP2A2,CYFIP1,DLG4,FMR1,GRIN1,GRIN2A,GRIN2B,GRM2,GRM4,GRM7,HUWE1,PRNP,SHANK3,SYNGAP1*	*ABI1,ACTB,ACTN1,ACTN2,ACTN4,AGAP2,AKT1,AOC3,ARC,ARHGAP32,ARID5A,ARL3,ARMC1,ATF1,BAG6,BLNK,BSG,CAMK2B,CAMK2D,CAMK2G,CAMLG,CAPN1,CDCA8,CDC37,CDK5,CDK5R1,CDK5R2,CHMP5,CHRM3,CHRM4,CLTC,CNKSR2,CPSF6,CREB1,CYFIP2,CYLD,C1orf94,DAZAP2,DBNL,DCTN1,DCTN2,DGUOK,DLGAP1,DLG1,DLG3,DMPK,DNAAF2,EGFR,EHMT2,FAM168A,FAM168B,FKBP8,FXR1,GIT1,GJD2,GLB1L2,GNAO1,GPBP1,GRIA1,GRIA4,GRM3,GYS1,HAX1,HCN1,HDAC5,HES1,HNRNPK,HOMER1,HSP90AA1,HSP90AB1,HYAL3,IFIT3,IGF1R,IQGAP1,ITGA2B,ITM2B,KALRN,KCNC1,KCNJ2,KPNA1,KPNB1,KRTAP6-1,KRTAP6-2,KRTAP8-1,KRTAP15-1,KRTAP19-3,KRTAP19-5,KRTAP19-7,KRTAP23-1,KRT75,KRT76,LASP1,LBR,LENG8,LRRC7,MAPT,MPDZ,MRPL11,MYCBP2,NBEAL2,NCKAP1,NINL,NOS3,NR2F6,NUFIP1,OSTM1,PAIP1,PARP1,PATJ,PDCD6IP,PDE4DIP,PEX5L,PLCG1,PPM1F,PPP1R9B,PRKN,PSEN1,PSMC1,PSMC2,PSMC4,PSMC5,PTPRR,PTTG1,RAD23A,RAD23B,RALYL,RBFOX2,RBM47,RBPMS,RBPMS2,RCHY1,RHOXF2,RIMS1,RRP7A,SCN5A,SLC6A3,SMAD2,SMN1,SNCA,SOX5,SQSTM1,SSBP2,SUOX,TAB2,TANC1,TCAF1,TEX37,TFAP2D,TIAL1,TNIK,TRIM55,TRIM63,TSC1,TSR2,TTC5,UBC,VARS1,WDYHV1,YWHAB,YWHAE,YWHAQ,YWHAZ,ZBTB32,ZFP36L2,ZMYM1*
***CAMK4***	Calspermin (Q16566)	Protein kinase superfamily, CAMK Ser/Thr protein kinase family, CaMK subfamily	*CALM1, CALM2, CALM3*	*HDAC4*	*ANKRD28,ANKRD52,ARHGEF7,CABIN1,CALML4,CALML5,CAMKK1,CAMKK2,CREBBP,CREB1,GIT1,GIT2,HDAC5,HSP90AA1,HSP90AB1,MEF2A,MEF2D,NEDD4,NOS3,NOTCH1,PPM1E,PPP6R2,RBPJ,SRF,YWHAE*
***CAMSAP1***	Calmodulin-regulated spectrin-associated protein 1 (Q5T5Y3)	CAMSAP1 family	*CALM1*	*HERC2*	*AK8,ARHGEF2,CAMK2D,CANX,CENPJ,CEP44,CEP104,CEP120,CEP128,CEP135,CEP162,CEP170,CTAG1A,DCTN1,DIPK1B,EGLN3,ESR2,FAM69B,HMGA1,HRAS,KIAA0753,KIAA1429,LCA5,LNX1,MECOM,MED4,NINL,NTRK1,ODF2,PCM1,PDE4DIP,POC1A,PRKAR1B,RBPJ,SKIL,SMAD1,SMAD9,SNAPC4,SPICE1,SPTAN1,SPTBN1,SRPK2,SSX2IP,SURF4,SURF6,TNIP2,TUBA1A,TUBA1B,TUBA1C,TUBA3C,TUBA3D,TUBA3E,UBAC1,YWHAE*
***CAPN2***	Millimolar-calpain, M-calpain (P17655)	Peptidase C2 family	*PAFAH1B1*	*APP,PAFAH1B1,PTEN,XPO1*	*ALDH7A1,AMPH,ASNS,ATG5,ATIC,ATXN3,BAK1,BAX,BCL2,BID,B3GNT2,CAPNS1,CAPNS2,CAPN1,CASP12,CAST,CAV3,CDK5,CDK5R1,CEBPA,CFTR,CHORDC1,CPEB3,CTH,DCP2,DNASE2B,DUSP19,DUX4,ECT2,EED,EFTUD2,EGLN3,FAM9C,FBXL12,GMDS,GNS,HK1,HNRNPA1,IL1A,ITM2B,JAK3,KIAA1429,KSR1,LSP1,MAN2A2,MAPK1,MAPK3,MAPT,MECOM,MSN,MYC,NAE1,NFATC2,NMT1,NSFL1C,NTRK1,NUDCD1,PAPSS1,PCYT2,PDIA4,PGLS,PLPBP,PRDM16,PSG8,PTMA,PTPN1,PTPRN,PXN,SNRPE,SOX2,STK26,STUB1,SUMO1,TATDN1,TIRAP,TLN1,TLN2,TNS1,TP53,TUBB2A,TUFM,TWF2,UBA3,UBE2I,UBXN1,YWHAE,ZDHHC17*
***CCL3***	Macrophage inflammatory protein 1-alpha (P10147)	Intercrine β (chemokine CC) family	–	–	*ACKR2,CCL3,CCL4,CCR1,CCR2,CCR3,CCR4,CCR5,CXCL2,CXCL10,CXCR2,CXCR3,CXCR5,IDE,IL1B,IL6,IL10,STAT3,TNF,UBQLN1,UBQLN2,ZFP36*
***CDH1***	Epithelial cadherin, CD antigen CD324 (P12830)	Cadherin family	*CADPS,CDH2,CTNNB1,DAG1,DAG1,SNAP25,SRCIN1,SYT7*	*CDKL5,CTNNB1,ERBB2,SHANK2*	*AAK1,ABCF3,ABI1,ABLIM1,ABLIM3,ABL2,ACADVL,ACBD3,ACTG1,ACTN1,ACTN4,ADAM9,ADAM17,ADCY9,ADD1,ADD3,ADGRL2,AFDN,AGFG1,AHNAK,AHSA1,AJAP1,AKAP2,AKT1,ALDOA,ANAPC10,ANK3,ANLN,ANP32B,ANXA1,ANXA2,ANXA4,AOPEP,AP2M1,ARFGAP2,ARFIP1,ARFIP2,ARGLU1,ARHGAP1,ARHGAP5,ARHGAP18,ARHGAP21,ARHGAP23,ARHGAP29,ARHGAP32,ARHGDIB,ARHGEF12,ARHGEF16,ARPC2,ARPIN,ARVCF,ASAP1,ATIC,ATP2B1,ATP2B4,ATXN2L,BAG3,BAIAP2L1,BAIAP2,BCAR1,BCL2L2,BEX1,BLID,BMPR2,BRCA1,BSG,BZW1,BZW2,CA9,CALD1,CAMK1D,CANT1,CAPG,CAPZA1,CAPZB,CARMIL1,CASKIN2,CASP3,CAST,CAVIN3,CAV1,CBLL1,CBL,CC2D1A,CCDC43,CCDC85C,CCDC120,CCL5,CCNA1,CCNB2,CCND1,CCSER2,CCS,CCT6A,CCT8,CDC27,CDC42EP1,CDC42EP4,CDC27,CDC42,CDCA3,CDH3,CDH10,CDK2,CDK5R1,CDK8,CD2AP,CD99,CD151,CEMIP2,CFTR,CGN,CHAF1A,CHMP2B,CHMP4B,CHMP5,CIAPIN1,CIP2A,CKAP5,CKS1B,CLIC1,CLINT1,CLMN,CNN2,CNN3,COBLL1,COL17A1,COPG2,CORO1B,CREBBP,CRKL,CRK,CRYAB,CSE1L,CSNK1A1,CSNK1D,CSNK2A1,CTNNA1,CTNNA2,CTNNA3,CTNND1,CTSB,CTTN,CXADR,CYP17A1,C1orf198,C1QBP,C6orf132,DAAM1,DAB2IP,DBN1,DBNL,DDR1,DDR2,DDX3X,DDX6,DDX39B,DENND4C,DHX29,DIAPH3,DKK3,DLG1,DLG5,DNAJB1,DNMT1,DOCK9,DSC3,DSG2,DST,DVL3,EBNA1BP2,ECT2,EEF1A1,EEF1D,EEF1G,EEF2,EFHD2,EFR3A,EGFR,EHBP1L1,EHBP1,EHD1,EHD4,EIF2A,EIF2S3,EIF3E,EIF4B,EIF4G1,EIF4G2,EIF4H,EIF5A,EIF5,ELFN2,ELMOD3,ELP4,EMD,ENO1,EPB41L1,EPB41L3,EPB41L4B,EPB41,EPHA2,EPN2,EPN3,EPSTI1,EPS8L1,EPS8L2,EPS15L1,EPS15,EP300,ERBIN,ERBB3,ERBIN,ERC1,ESR2,ESYT1,ESYT2,EVPL,EXOC3,EZR,E2F1,FAM83B,FAM83H,FAM91A1,FAM110A,FAM110C,FAM126A,FAM126B,FAM171B,FASN,FERMT1,FER,FGFR1,FLNA,FLNB,FLOT1,FLOT2,FMN1,FMNL2,FNBP1L,FRMD5,FRS2,FSCN1,FTH1,FYN,F11R,GAB1,GALNT12,GAPVD1,GCN1,GGA3,GIGYF2,GIPC1,GLOD4,GM2A,GMNN,GNA12,GNA13,GOLGA2,GOLGA3,GOLGA5,GOLGA8B,GOLGB1,GORASP2,GPC1,GPRC5A,GPRIN1,GSK3B,GSPT1,HADHB,HCFC1,HDAC1,HDAC2,HDLBP,HECTD1,HEMGN,HLA-C,HMGB2,HN1L,HN1,HNRNPK,HRAS,HSD17B3,HSPA1A,HSPA1B,HSP90AA1,HSP90AB1,HSPA1A,HSPA5,HSPA8,HSPA9,HSPD1,H1FX,H3C1,IDH1,IGBP1,IGF2R,IGHA1,IL6ST,ILK,INAVA,INPPL1,IQGAP1,IRAK1,IST1,ISYNA1,ITGAE,ITGA3,ITGA6,ITGB1,ITGB7,ITPRID2,IVL,JCAD,JUP,KANK2,KCNN4,KDF1,KEAP1,KIAA1217,KIAA1522,KIAA1671,KIDINS220,KIFC3,KIF5B,KLC2,KLF4,KLHL7,KLRG1,KRAS,KRT1,KRT8,KRT9,KRT17,KRT18,KTN1,LAD1,LARP1,LASP1,LBR,LDHA,LGALS3BP,LIMA1,LIMCH1,LLGL1,LMNA,LMO7,LPP,LRATD2,LRPPRC,LRRC7,LRRC57,LRRC59,LRRFIP1,LSR,LUZP1,LYPLA2,LZTS2,MACC1,MACF1,MACROH2A1,MAGI1,MAGI3,MAPKAPK3,MAPRE1,MAP2K1,MAP4,MARCKS,MARK2,MARK3,MARVELD2,MAVS,MB21D2,MDM2,MET,MICALL1,MINK1,MMP9,MMTAG2,MPP7,MPRIP,MPZL1,MRE11,MROH1,MRTFB,MSANTD3,MTA3,MYF5,MYH9,MYO1B,MYO6,MYO7A,NANS,NAT2,NAV2,NCK1,NCK2,NDRG1,NDRG2,NEBL,NECTIN1,NECTIN2,NECTIN4,NEDD9,NF2,NFE2L2,NFKBIE,NGDN,NHSL1,NHS,NIBAN2,NIPSNAP1,NIPSNAP2,NKD2,NLRP2,NOP56,NOTCH1,NOTCH2,NOTCH3,NSFL1C,NUDC,NUDT5,NUMB,NUMBL,NUMB,NUP214,N4BP1,OCLN,OLA1,PACSIN2,PAG1,PAICS,PAK2,PAK4,PAK6,PALM3,PARD3,PARK7,PARVA,PATJ,PCBP1,PCBP2,PCDH1,PCDHGC3,PCMT1,PCNX3,PDAP1,PDLIM1,PDLIM5,PDLIM7,PDXDC1,PEAK1,PFKP,PFN1,PGRMC2,PHACTR2,PHACTR4,PHLDB2,PHLPP1,PICALM,PIK3R1,PIP5K1C,PI4KA,PKD1,PKM,PKN2,PKP2,PKP3,PKP4,PLCB3,PLEC,PLEKHA1,PLEKHA2,PLEKHA5,PLEKHA6,PLEKHA7,PLEKHO2,PLIN3,PLSCR1,PLSCR3,POF1B,POLR2E,POLR2M,POU5F1,PPFIBP1,PPL,PPME1,PPP1CA,PPP1R13B,PPP1R13L,PPP1R18,PPP1R37,PRDX1,PRDX6,PRKD1,PROM2,PRRC2B,PSEN1,PSMB6,PSMC3IP,PTK2,PTPN1,PTPRD,PTPRG,PTPRJ,PTPRM,PTPRQ,PTPRS,PUF60,PVR,PXN,RAB1A,RAB8B,RAB10,RAB11B,RACGAP1,RACK1,RAD17,RAI14,RALBP1,RALGDS,RANBP1,RANGAP1,RAN,RAPH1,RARS1,RB1CC1,RDX,RELL1,RNF2,RPL6,RPL7A,RPL14,RPL15,RPL23A,RPL24,RPL29,RPL34,RPL36AL,RPL36A,RPL37,RPS2,RPS26,RSL1D1,RTN4,RUVBL1,SARG,SBSN,SCARB1,SCEL,SCRIB,SCYL1,SCYL2,SDCBP,SEC24B,SEPTIN2,SEPTIN7,SEPTIN9,SERBP1,SET,SFN,SFRP2,SHB,SHROOM3,SHTN1,SH3D19,SH3GL1,SH3GLB1,SH3GLB2,SH3KBP1,SH3PXD2B,SH3RF1,SIPA1L3,SIRPA,SKP2,SLC1A5,SLC2A14,SLC3A2,SLC4A7SLC9A3R1,SLC9A3R2,SLC12A2,SLC16A3,SLC30A1,SLC38A1,SLC45A2,SLIRP,SLK,SMAD3,SMAD7,SNAI1,SNAI2,SNAP23,SNAP29,SND1,SNX1,SNX2,SNX5,SNX9,SORBS1,SPAG1,SPECC1,SPRED1,SPRR3,SPTAN1,SPTBN1,SPTBN2,SRC,SRGAP2,SSH3,STAT1,STAT2,STEAP3,STIM1,STK24,STK38,STXBP6,STX4,STX5,STX17,SVIL,SWAP70,SYAP1,SYNJ1,SYNJ2,S100A11,S100P,TAGLN2,TANC1,TAX1BP3,TBC1D10A,TBC1D10B,TBC1D22B,TBP,TENM3,TES,TGFB1,TGM2,THRSP,TJP1,TJP2,TLN1,TMEM51,TMEM87A,TMEM199,TMOD1,TMOD3,TMPO,TNKS1BP1,TNS3,TP53BP2,TPD52L2,TPD52,TRIM25,TRIM29,TRIP6,TRIP11,TTC7A,TTK,TTN,TUBB4B,TUBB,TWF1,TWF2,TXNDC9,UACA,UBAP2,UBC,UBFD1,UCHL3,UHRF1,UNC45A,USO1,USP6NL,USP8,USP9X,USP15,USP43,UTRN,VAMP3,VANGL2,VAPA,VAPB,VASN,VASP,VCL,VCPIP1,VEZT,VSIG10L,WASF2,WIPF2,WWOX,XRCC5,XRN1,YAP1,YES1,YKT6,YWHAB,YWHAE,YWHAZ,ZBTB48,ZC3H15,ZC3HAV1,ZDHHC5,ZEB1,ZFYVE16,ZG16B,ZNF185,ZNF510,ZYX*
***CDH2***	Neural cadherin, CD antigen CD325 (P19022)	Cadherin family	*CDH13,CTNNB1*	*CTNNB1,ERBB2*	*ACTA1,ADIPOQ,AGPAT5,APBB1,ARF5,AURKA,BOC,CASP8,CAV3,CCNA2,CCND1,CDH4,CDH5,CDH7,CDH8,CDH9,CDH10,CDH11,CDH15,CDH17,CDH18,CDON,CHEK2,CHPT1,CREBBP,CTNNA1,CTNNA3,CTNND1,DBN1,DDR1,DDR2,DGUOK,DNAAF2,DSTYK,EGFR,ERMP1,EXOC5,FBXO45,FGFR1,GAA,GHITM,GNA12,GNA13,HMMR,HTT,IARS2,INSIG1,JUP,KIAA1429,KTN1,LAMP1,LRRC7,MAPK6,MDC1,MRPL23,MRPL49,MRPL58,MTHFD1,MTNR1B,NACA2,NENF,NTRK1,NUFIP1,PC,PDIA4,PIGU,PKP4,PPT2,PRPH,PSEN1,PTBP3,PTPN1,RAB8B,RAD51,RNF4,RPL3,RPL8,RPL14,RPL21,RPL23,RPL32,RPS16,RPS26,RRAGB,RYK,SARS2,SEC14L1,SKP1,SLC9A3R1,SPTBN1,SSR4,SUPT5H,TAX1BP3,TMEM256,TMX4,TPM4,TUBB3,TUBB8,UBC,UQCC1,VMP1*
***CDH13***	Truncated cadherin (P55290)	Cadherin family	–	–	*ADIPOQ,AGPAT5,ARF5,AURKA,C16orf58,CASP8,CCND1,CHEK2,CHPT1,DGUOK,DNAAF2,DSTYK,ERMP1,GHITM,HAX1,HMMR,HTT,INSIG1,MAPK6,MDC1,MTNR1B,NUFIP1,PIGU,PTPN1,RAD51,RPL23,RRAGB,SEC14L1,SPCS2,TMX4,TUBB3,TUBB8,UQCC1,VMP1*
***CDH23***	Otocadherin (Q9H251)	Cadherin family	*DLG4,MYO1C,PCDH15*	*CYFIP1,DLG4*	*ABI1,ADGRV1,CD160,CLK1,CLRN1,C9orf72,GOPC,LYN,MYO7A,MYO15A,NCKAP1,NXN,PPME1,SLC9A3R2,SOD1,SPDL1,TARDBP,TMC1,USH1C,USH1G,USH2A,WASF1,WHRN*
***CDHR1***	Photoreceptor cadherin (Q96JP9)	Cadherin family	–	–	*ABCA4,ATP5PO,CERKL,CNGA1,DNAJC16,EYS,KCNV2,LRIT1,LRIT2,MINPP1,PCDHGB4,PROM1,PROM2,RGS9BP,ROM1,RYK,TCTA,TMEM30B,TMEM59L,TM4SF5*
***CHP1***	Calcineurin B homologous protein 1 (Q99653)	Calcineurin regulatory subunit family, CHP subfamily	*KIF1B,PPP5C,SLC9A1*	*SHMT2,XPO1*	*AAR2,BRF2,CANX,CD44,CHORDC1,CLN6,COG4,CTDP1,DUSP6,DUSP22,ELAVL1,FANCD2,GAPDH,HRAS,HYAL2,IFITM3,IFT27,KRAS,HMMR,LAMP1,LYVE1,MAD2L2,MOV10,MS4A4A,NXF1,PPT1,PRSS23,PTP4A3,P2RY6,RIPK4,SLC2A4,SLC9A2,SLC9A3,SLC9A4,SLC9A5,SLC15A3,SLC38A1,STAB2,STK17B,TMEM216,UBC,UNC93B1,WDTC1,YWHAE*
***CIB1***	Calcium and integrin-binding protein 1 (Q99828)	–	*PAK1,RAC3*	*RPTOR*	*ACTN4,AKT1S1,AURKB,CALCB,CDK4,CD27,CEACAM6,CRY2,DEPTOR,EGLN3,EIF4G1,EXOSC10,FKBP1A,FUCA1,GLIS3,ITGA2,ITGA2B,ITGA5,ITGB3,KIAA1324,LSS,MAPKAP1,MAP3K5,MLST8,NBR1,NME4,NRIP1,ONECUT3,PAX3,PAX7,PLK2,PLK3,PPP3CB,PRKDC,PRR5,PSEN1,PSEN2,PLK2,PLK3,PTK2,RICTOR,SCAF1,SSX7,STAB2,TERT,TMC5,TMC7,TMEM95,TSGA10IP,UBR5,UPK1A,WASL,ZBTB49,ZDHHC17*
***CLN3***	Batten disease protein (Q13286)	Battenin family	*DNAJC5,KCNIP3*	*ATP2A2,DBH,SIGMAR1,XPO1*	*ABCD3,ADGRL2,AFG3L2,ARF3,ARF4,ARF5,ARMC6,ATG7,ATP1A1,ATP5MG,AUP1,B3GALT2,CALU,CAND1,CCDC47,CDS2,CKAP4,CLGN,CLN5,CLN6,CLN8,COX15,CPT1A,CSE1L,CYC1,DDOST,DDX20,DNAH17,DPM1,ECPAS,ELAVL1,EMD,ERLIN2,ESYT2,FADS2,FANCI,FAR1,FKBP8,GANAB,GCN1,GEMIN4,GRPR,HACD3,HOOK1,HPF1,HSD17B12,ILVBL,IMMT,IRAK1,LPGAT1,MAPK14,MECOM,MFSD8,MFSD12,MICOS13,NPEPPS,NUP93,NUP205,PHB,PHGDH,PPT1,PRDM16,PRKDC,PTH1R,RAB2B,RARS2,RCN2,RIF1,RPN1,RPN2,SAMM50,SBDS,SCAMP3,SEC61A1,SEC61A2,SFXN1,SFXN3,SFXN4,SLA1,SLC5A5,SLC22A9,SLC25A1,SLC25A3,SLC25A4,SLC25A5,SLC25A6,SLC25A10,SLC25A11,SLC25A13,SLC25A22,SLC35B2,SSR4,STRA6,STX5,SYP,TECR,TELO2,TFRC,TMEM33,TMPO,TMPRSS11B,TPP1,TPRA1,TRIP13,UNC45A,UQCRC2,VDAC2,VTI1A,XPO5,XPOT*
***CPNE1***	Copine I (Q99829)	Copine family	*PPP5C*	*CCDC22,RBM12*	*ACP3,ACTB,ARRB2,ATP6V0C,ATP8A1,ATP11B,BAG3,BCOR,BST2,BUB3,CDA,CDC42BPB,CENPF,CFTR,COCH,COPS5,CORO1B,CPNE3,CPNE4,DNAJC13,FN1,HNRNPH1,HSPA2,KIAA1217,KLK5,LHX6,LIG4,MAGT1,MAP2K1,MYCBP2,NONO,NTRK1,PDCD6,PDLIM1,PITPNM2,PLEKHA7,PTX4,PYGB,RAB3D,RAP1B,RDX,SDHA,SKIL,SORBS3,SPAG4,SPTBN1,STOM,ST13,STK3,TELO2,TIMM13,TRIM25,TTLL12,UBE2M,UBE2O,UIMC1,VNN1,YWHAE,YWHAZ*
***CPNE5***	Copine V (Q9HCH3)	Copine family	*SNTB2*	–	*ANKRD52,ARHGAP8,BICD2,CHD4,DDN,DHRS7,DSCC1,DTNB,FAM114A2,GATAD2A,GATAD2B,GDA,GEMIN5,GNG7,GPR88,KCNJ4,LAMP5,ICAM5,LRRC10B,MBD3,MBNL3,MTA1,MTA2,MTMR3,NAPG,NCK1,NCK2,RABEP2,RABGEF1,RAB1B,RAD21,RAD54B,RGS9,RNF6,SATB2,SEC22A,SNX9,SPDL1,TAFA4,UBA2,UBR4,UROD,USO1,UTRN,WDHD1,YWHAE*
***CTNNB1***	Beta-catenin (P35222)	Beta-catenin family	*CACYBP,CALM1,CDH1,CDH2,DLG4,HMGB1,LIN7B,LRP2,LRP6,LRRK2,MYO1C,PRKCA,PRKCG,SMARCA4,TPM3*	*APC,APP,ATP2A2,CTNND2,CUL3,CYFIP1,DISC1,DLG4,DKK1,ERBB2,FMR1,GRIN1,GRIN2B,HDAC4,HERC2,HUWE1,PTEN,XPO1,SYNGAP1,TCF4,TCF7*	*ABCE1,ABCF2,ABCF3,ABL1,ABL2,ACLY,ACO2,ACP1,ACTB,ACTG1,ACTL6A,ACTN1,ACTN4,ADSL,AFDN,AHCTF1,AHCY,AHR,AJAP1,AKAP12,AKT1,AMER1,AMOT,ANKRD35,ANP32A,APC2,API5,APOE,APPL1,AP2M1,AP3D1,ARF1,ARFGEF1,ARFGEF2,ARHGAP21,ARHGAP32,ARHGEF4,ARID4B,ARL6,ARMC8,ARNT,ARVCF,ASH2L,ATAD3A,ATF2,ATIC,ATP1A1,ATP5F1A,ATP5F1B,ATXN2L,AURKA,AXIN1,AXIN2,BAIAP2,BAP1,BBS2,BCAR1,BCCIP,BCL2L1,BCL3,BCL6,BCL9,BCL9L,BMPR1A,BOC,BRCA1,BRCC3,BRIX1,BTF3,BTRC,CA9,CAD,CALCOCO1,CAND1,CAPRIN1,CAPZA1,CAPZA2,CARM1,CASP3,CAV1,CBFB,CBL,CBX3,CBX5,CBY1,CCDC8,CCDC120,CCNA1,CCNA2,CCND1,CCNE1,CCT2,CCT4,CDC27,CDC37,CDC42,CDC73,CDH3,CDH5,CDH6,CDH7,CDH8,CDH9,CDH10,CDH11,CDH15,CDH18,CDK2,CDK5R1,CDK6,CDK9,CDON,CEBPA,CEP128,CFAP298,CFL1,CFTR,CHD4,CHD8,CHUK,CITED1,CKAP5,CLTA,CNOT2,COPA,COPE,COPS2,COPS3,COPS5,COPS8,CORO1B,CPSF7,CREB1,CREBBP,CRYAB,CRYL1,CSNK1A1,CSNK1E,CTBP1,CTBP2,CTNNAL1,CTNNA1,CTNNA2,CTNNA3,CTNNBIP1,CTNND1,CUL1,CUL4A,CUX1,CXXC4,CYFIP2,CYLD,DACT1,DACT2,DACT3,DAD1,DARS1,DAZAP2,DCX,DDB1,DDX1,DDX3X,DDX5,DDX17,DDX39B,DHX9,DIMT1,DLGAP1,DLG5,DLGAP1,DLL1,DNAJC8,DNMT1,DOT1L,DSC3,DVL1,DVL2,DVL3,DYNC1H1,EED,EEF1D,EEF2,EFTUD2,EGFR,EGLN3,EGR1,EIF2S1,EIF3J,EIF4A3,EIF6,ELAVL1,EMD,EPAS1,EPCAM,EPHA2,EP300,EP400,ERBIN,ERBIN,ERLIN1,ERMN,ESR1,ESR2,ETS1,EWSR1,EZH2,EZR,E2F3,FAF1,FANCA,FANCC,FANCD2,FANCE,FANCF,FANCG,FARSA,FAS,FBXO45,FBXW7,FBXW11,FERMT2,FER,FGFR1,FHIT,FHL2,FKBP4,FLII,FLOT2,FLT1,FOXB1,FOXM1,FOXO3,FOXO4,FRMD5,FUS,FXR1,FYN,GANAB,GAPDH,GEMIN5,GFPT1,GID8,GJA1,GLIS2,GPC1,GRIA1,GRIA2,GRIA3,GRIA4,GRIP1,GSK3A,GSK3B,G3BP1,HDAC1,HDAC2,HDAC6,HDAC7,HDGF,HDLBP,HECTD1,HERC5,HIC1,HIF1A,HINT1,HMBOX1,HMGB2,HMMR,HNF1A,HNF1B,HNF4A,HNRNPA1,HNRNPA2B1,HNRNPC,HNRNPK,HNRNPM,HNRNPU,HRAS,HSP90AA1,HSP90AB1,HSP90B1,HSPA1A,HSPA1B,HSPA5,HSPA8,HSPA9,HSPB1,HSPD1,HSPE1,HSPH1,HTT,H1-2,H3C1,H4C1,IDH3B,IFRD1,IGF1R,IKBKB,ILF3,ILK,IMMT,IMPDH2,INSR,IQGAP1,IRF2BPL,IRS1,ISG15,ITGA3,ITM2B,JADE1,JOSD1,JOSD2,JRK,JUN,JUP,KANK1,KAT2A,KAT2B,KCTD1,KDM1A,KDR,KHDRBS1,KIAA1109,KIAA1429,KIFAP3,KIF3A,KIF3B,KIF5B,KIF22,KLC2,KLF4,KLF5,KMT2A,KMT2D,KRAS,KSR2,LAD1,LAMP1,LARS1,LATS2,LDHB,LEF1,LEO1,LGALS3,LGALS9,LIG3,LIMA1,LIMCH1,LMNA,LMNB1,LMO7,LNX1,LRP5,LRRFIP1,LRRFIP2,LSM12,LUC7L2,LZTFL1,LZTS2,L1TD1,L3MBTL3,MACF1,MAGI1,MAGI2,MAGOHB,MAPK6,MAPK8,MAPK9,MAPRE1,MAP1LC3B,MAP2K5,MAP3K2,MARCKSL1,MATR3,MCM2,MCM5,MCM7,ME1,MED9,MEN1,MET,MIB1,MIS12,MKI67,MLLT10,MOV10,MPRIP,MSN,MST1R,MTHFD1,MUC1,MYBBP1A,MYC,MYH9,MYH10,MYH14,MYL6,MYL12B,MYO1B,MYO5B,MYO7A,NANOG,NCL,NCOA2,NDRG1,NDRG2,NDUFS4,NEK2,NEURL2,NFKB1,NF2,NHSL1,NLK,NONO,NOS3,NOTCH1,NPM1,NPRL2,NR4A1,NR5A1,NR5A2,NRAS,NSD2,NT5E,NTRK1,NUDC,NUDCD1,NUDT5,NUMA1,NUMB,NUP93,NUP98,NUP214,NXF1,OBSL1,OTUB1,OTUB2,OTUD1,OTUD7B,OVOL2,OXA1L,PABPC1,PABPC4,PAN2,PARD3,PARK7,PARP1,PARP2,PARP11,PAX3,PA2G4,PBDC1,PCBP1,PCBP2,PCP2,PCNA,PDCD6IP,PDE4DIP,PDIA3,PDLIM1,PDLIM5,PDPK1,PECAM1,PEX5L,PGK1,PHB2,PHF19,PHGDH,PHLDB2,PIK3CA,PIK3R1,PIN1,PITX2,PKD1,PKM,PKP2,PKP3,PKP4,PLEKHA6,PLEKHA7,PLK1,PML,PNO1,POLDIP3,POLR1C,POLR2A,POU5F1,PPARG,PPFIA3,PPIE,PPM1A,PPP1CB,PPP1R9B,PPP1R12A,PPP1R12B,PPP2R1A,PPP2R5A,PRC1,PRDX5,PRKAA1,PRKDC,PRKN,PROM1,PROP1,PRPF6,PRPF19,PRPF38B,PSEN1,PSMA5,PSMB6,PSMC3,PSMC5,PSMC6,PSMD1,PSMD4,PSMD7,PTBP3,PTGS2,PTH1R,PTK2,PTK7,PTMA,PTN,PTPA,PTPN1,PTPN11,PTPN12,PTPN13,PTPN14,PTPRB,PTPRC,PTPRF,PTPRG,PTPRJ,PTPRK,PTPRM,PTPRO,PTPRQ,PTPRU,PTPRZ1,PXN,PYGO2,P4HB,RABAC1,RAB8A,RAB8B,RAB11A,RACK1,RAC1,RAD18,RAE1,RALGDS,RALY,RANBP2,RAPGEF2,RARA,RARS1,RB1CC1,RBBP5,RBM17,RBM39,RBMX,RBX1,RELA,RET,RNF4,RNF6,RNF14,RNF138,RNF220,RNPS1,RPA1,RPA2,RPLP0,RPLP2,RPL3,RPL4,RPL7A,RPL8,RPL9,RPL10A,RPL10,RPL11,RPL12,RPL13,RPL18A,RPL18,RPL21,RPL23A,RPL23,RPL28,RPL30,RPL31,RPL35A,RPL41,RPS2,RPS3A,RPS3,RPS4X,RPS5,RPS6,RPS8,RPS10,RPS11,RPS14,RPS15A,RPS16,RPS17,RPS20,RPS24,RPS27A,RPS27,RPS28,RPSA,RTN4,RUNX1,RUNX2,RUNX3,RUVBL1,RUVBL2,RXRA,RYK,SAPCD1,SATB1,SCRIB,SEC13,SEC16A,SEC24B,SEC24C,SERBP1,SET,SETD1A,SETD7,SFN,SFPQ,SH3KBP1,SIAH1,SIPA1L1,SIPA1L3,SIRT1,SKP1,SLAMF7,SLC2A4,SLC9A3R1,SLC9A3R2,SLC25A1,SLC25A3,SLC25A5,SLC25A11,SLC25A13,SMAD2,SMAD3,SMAD4,SMAD7,SMARCA5,SMURF1,SNCA,SNRPD1,SNRPD2,SOCS2,SORBS3,SOX1,SOX2,SOX4,SOX6,SOX17,SPATA13,SPECC1L,SPECC1,SP1,SRC,SRP9,SRRM2,SSRP1,STAT1,STAU1,STIP1,STK4,STK39,STRN3,STUB1,SUCLG1,SUMO1,SUV39H1,SVIL,SYNCRIP,TADA2A,TADA3,TARDBP,TAX1BP1,TAX1BP3,TBL1XR1,TBL1X,TBX3,TBX5,TCF3,TCF7L1,TCF7L2,TCP1,TERT,TES,TFAP2A,TGFBR2,TGIF1,THRAP3,TJP1,TLE1,TLE3,TLN1,TMOD3,TMPO,TNFAIP3,TNIK,TNRC6B,TOB1,TOP2A,TPM1,TP53BP2,TRAF4,TRIB2,TRIM25,TRIM28,TRIM33,TRIM37,TRPM7,TRRAP,TSC1,TUBA1A,TUBB4B,TUBB,TXN,UACA,UBA1,UBA52,UBB,UBC,UBE2B,UBE2D1,UBE2D2,UBE2L6,UBE2N,UBE2R2,UBE2S,UBE3A,UBR5,UCHL1,UCHL3,UHRF2,UNK,UQCRB,USO1,USP2,USP4,USP7,USP9X,USP10,USP11,USP13,USP15,USP17L2,USP18,USP20,USP21,USP29,USP30,USP33,USP36,USP37,USP43,USP49,USP53,UTP14A,VASP,VCL,VEZT,VHL,VRK3,WBP2,WNT4,WNT9A,WWP2,WWTR1,XRCC5,XRCC6,YAP1,YBX1,YES1,YOD1,YWHAB,YWHAE,YWHAQ,YWHAZ,ZBTB33,ZFYVE9,ZIC3,ZMIZ1,ZNF326*
***C1QA***	Complement C1q subcomponent subunit A (P02745)	C1q protein family	–	*C4A,C4B,NLGN3*	*APOA1,BAG6,CALR,CD33,CRP,CTSS,C1QB,C1QBP,C1QC,C1R,C1S,DCN,FANCD2,FN1,FZD1,FZD2,FZD4,FZD7,FZD8,GNPNAT1,HIPK3,HSPB2,ITM2B,LRP1,PIAS2,PPP1CA,PPP1CC,PRKCE,RAC1,SERPING1,SGTA,SGTB,SLC25A47,TYROBP,UBQLN1,UBQLN2,VSIG4,YWHAZ*
***DAG1***	Dystroglycan (Q14118)	Dystroglycan family	*CDH1,LAMA1,SNTA1,SNTB2*	–	*ABCC4,ACTN1,AGR2,AGRN,AGR3,ANK3,AZGP1,B3GALNT2,CAV1,CAV3,CDH5,CD9,CEACAM21,CHRNB1,CLIC4,CORO7,CSK,DMD,DNAJB1,DRP2,DTNB,EGFLAM,ELAVL1,FKBP9,FYN,GINM1,GOT1,GOT2,GPC1,GRB2,HARS1,HFE,HLA-DPA1,HRAS,HSPA5,HSPG2,HTR3C,IL13RA2,IQSEC1,ITGB1,KCNJ10,KIAA1429,KIDINS220,KRAS,LAMA2,LAMA5,LAMB1,LAMP1,LATS2,LCP1,LGALS7,LGALS8,LGALS9,LGALS9C,MAP2,MDH1,NCK1,NCSTN,NOS3,NRAS,PCDHA8,PCDHAC2,PCDHB11,PCDHGA5,PCDHGB2,PCDHGB4,PDIA4,PIK3R1,PMT4,POF1B,POMGNT1,POMGNT2,POMK,POMT1,POMT2,PPP1CB,PRX,PSEN1,PSMA2,PSMB2,PSMB4,PTK2,RAPSN,RBBP4,RNF4,RPIA,SGCA,SGCB,SGCD,SGCE,SGCG,SH3KBP1,SHC1,SLC3A2,SLIT2,SRC,SSPN,TCTN2,TLN1,TMEM106A,TNF,TNFSF8,TNIP2,TPI1,TRIM25,TSPAN15,TUBA1A,UBR7,UTRN,VCL,VPS45,WWC1,WWP1,YAP1,YWHAE*
***DAXX***	Fas death domain-associated protein (Q9UER7)	DAXX family	*SLC9A1*	*CUL3,DISC1,PTEN,TCF4,TOP2B,XPO1*	*ABCF1,ACVR2A,ADNP,AFF4,AIRE,AMOTL2,ARHGAP22,ATF7IP,ATM,ATRX,AXIN1,BAZ1B,BRMS1L,BTBD6,BUB1B,CARD9,CARM1,CASQ2,CA12,CBS,CBX1,CBX5,CCDC86,CDC20,CDCA7L,CDKN2A,CEBPB,CENPB,CENPC,CEP63,CEP70,CFLAR,CHD4,CHD9,CHMP2A,CHMP3,CHMP4A,CHMP4B,CLK2,CLK3,CMSS1,COIL,CREB1,CREBBP,CREB3L1,CSNK2A1,CXXC1,DAPK3,DBF4,DDX27,DEK,DKC1,DMAP1,DNMT1,DNMT3A,DOT1L,ERVK-9,ESR1,ETS1,FADD,FAM9B,FANCA,FAS,FGF8,FTH1,FTSJ3,FZR1,GAR1,GAS8,GNL2,GOLGA2,GOLGA6L9,GPC3,GRIPAP1,GRK5,HABP4,HBA1,HDAC1,HDAC2,HDAC3,HIPK1,HIPK2,HIPK3,HIST1H2BB,HIST2H2BE,HIST2H3A,HIST2H3D,HIST3H3,HIST4H4,HNRNPCL1,HP1BP3,HSF1,HSF4,HSPB1,HSPB2,H1-2,H2AC4,H2BC13,H3C1,H3C3,H3F3C,H3-3A,H3-3B,H4C1,H4C6,H4C11,H4C14,ING1,JUN,KAT7,KCTD4,KIF5B,KPNA4,KRI1,KRR1,LARP7,MACF1,MAD2L1,MAP3K5,MAPK8,MCRS1,MDM2,MDM4,MEN1,MIPOL1,MKI67,MKRN1,MLLT3,MLLT6,MLLT10,MPHOSPH8,MPHOSPH10,MRE11,MSANTD4,MX1,MYH11,NAF1,NBN,NCBP3,NCL,NECAB2,NGDN,NHP2,NOL3,NOLC1,NR3C1,NR3C2,NSA2,NSD3,PABPC4L,PARK7,PARP1,PAX3,PAX5,PBXIP1,PDCD4,PHF14,PIBF1,PIN1,PLAGL1,PML,PNMA1,PNN,POGZ,PPAN,PPHLN1,PRDM10,PRMT1,PYHIN1,P2RY11,RAD50,RAI1,RASSF1,RELA,RELB,RFC1,RFC2,RFC3,RIPK1,RIPK3,RNF4,RNPS1,RPL8,RPL10A,RPL13,RPL23,RPL26L1,RPS16,RSL1D1,RS1,SAP130,SDC2,SENP2,SENP3,SENP7,SERBP1,SETD1A,SETD1B,SETDB1,SETDB2,SF3B2,SIN3A,SIN3B,SLC2A4SMAD4,SMARCA5,SNAI2,SNW1,SPECC1,SPEN,SPIN2B,SPN,SPOP,SP100,SRRM2,SRSF12,SSX2IP,STAT3,STK4,STUB1,SUDS3,SUMO1,SUMO1P1,SUMO2,SUMO3,SUPT16H,SURF6,SUV39H1,TASOR,TASOR2,TAX1BP1,TCF3,TCF7L2,TERT,TFIP11,TGFB1,TGFBR2,TNFRSF1A,TNIP2,TNRC18,TONSL,TOP2A,TP53,TP63,TP73,TRAF3,TRIM21,TRIM27,TRIM28,TRIM54,TSG101,UBB,UBC,UBE2I,UBTF,USH1G,USP7,UTP3,VPS25,WDFY3,WHAMMP3,XPO7,ZBTB2,ZBTB11,ZBTB26,ZC3H18,ZDHHC17,ZNF280C,ZNF354A,ZNF777*
***DLG4***	Postsynaptic density protein 95 (P78352)	–	*ATP2B2,CALM1,CALM3,CAMK2A,CDH23,CTNNB1,KIF1B,LIN7A,LIN7B,LRP2,NLGN1,NRXN1,PCDH15,PCLO,PRKCA,PRKCG,PRRT2,PTK2B,SNAP25,SNTB2,SYN1,SYN2,SYT1,TNR,TPM3*	*APC,APP,ATP1B1,ATP6V1A,CDKL5,CNIH2,CNIH3,CTNNB1,CTNND2,CUL3,CUX2,CYFIP1,DRD2,ERBB2,FMR1,FOLH1,GRIK1,GRIN1,GRIN2A,GRIN2B,GRIN2C,GRIN2D,HTR2A,KCND2,LRRTM1,NLGN2,NLGN3,NLGN4X,NRXN1,PHF8,PTEN,SCN2A,SHANK2,SHANK3,SLC12A5,SYNGAP1,PRNP,SYN2,TRAF3IP2,YWHAG*	*AASS,ABCG5,ABHD5,ABI1,ABLIM1,ABL2,ABR,ACAT1,ACOT7,ACO2,ACTB,ACTG1,ACTN1,ACTN2,ACTN4,ADAM20,ADAM22,ADCK5,ADCY3,ADGRA1,ADGRB1,ADGRB3,ADRB1,ADRB2,AFARP1,AGAP2,AGBL5,AJAP1,AKAP5,AKR1A1,ALDH2,ALDH5A1,ALDOA,ALDOC,ALS2,ANKRD24,ANKS1A,ANKS1B,ANK2,ANK3,ANXA5,APPL1,AP2A1,AP2B1,AP3B2,ARAP2,ARC,ARF1,ARF3,ARF5,ARGLU1,ARHGAP32,ARHGEF26,ARPC4,ASAP1,ASIC2,ASIC3,ASRGL1,ATN1,ATP1A1,ATP1B2,ATP2B1,ATP2B4,ATP4A,ATP5F1A,ATP5F1B,ATP5F1C,ATP5F1D,ATP5PO,ATP6V0D1,ATP6V1B2,ATXN2,A4GALT,BAIAP2,BASP1,BCL9L,BEGAIN,BEST4,BMPR1B,BMP8A,BTBD11,BTN2A3P,B4GALT1,CACHD1,CACNA2D1,CACNG2,CACNG3,CACNG4,CACNG7,CACNG8,CAMK2B,CAMK2D,CAND1,CAPZA2,CAP1,CASK,CASP10,CBLB,CCM2L,CC2D1A,CDC5L,CDC27,CDH4,CDK5,CDK7,CDK11A,CDK15,CDK17,CDRT15,CD46,CEBPD,CELSR1,CFAP61,CFL1,CHERP,CHRM1,CIDEA,CIT,CKMT1A,CLCNKB,CLN5,CLTC,CLU,CNGB1,CNKSR2,CNKSR3,CNTN1,CNTN3,CNTNAP1,COL27A1,COQ10A,CPNE7,CPSF7,CPT1C,CPXCR1,CRHR1,CRIPT,CRYM,CS,CSHL1,CSMD2,CTAGE1,CYCS,CYFIP2,CYLD,CYP24A1,CYSLTR2,C1orf52,C1S,DAPK1,DBI,DBN1,DCAF7,DCX,DDAH1,DENND2A,DERL2,DGKI,DGKZ,DIAPH2,DIAPH3,DIDO1,DIP2B,DLD,DLGAP1,DLGAP2,DLGAP3,DLGAP4,DLGAP5,DLG1,DLG2,DLG3,DMRT1,DNAH7,DNMT1,DNM1,DOC2B,DOK1,DPYSL2,DQX1,DSTN,DUSP10,DUX3,DYNC1H1,DYNLL1,DYNLL2,EEF1A1,EEF1G,EFNB2,EIF2S1,EIF4A1,EIF4A2,EIF4E2,EIF4G2,ELAVL1,ENO1,ENO3,ENPP1,ENPP2,ENPP3,EPB41L3,EP400,ERBB4,ESX1,EXOC4,EXOC7,EZH2,FABP3,FAH,FAM81A,FBXL17,FBXO2,FBXO6,FCHSD1,FGD5,FH,FKBP1A,FLNB,FMNL3,FMOD,FOXQ1,FRAS1,FRMPD3,FRMPD4,FRRS1L,FSCN1,FYN,FZD1,FZD2,FZD4,FZD7,GABBR2,GAB1,GAB3,GALNT7,GALNT10,GAPDH,GAS2L2,GDA,GDI1,GDI2,GIT1,GIT2,GLOD4,GLUD1,GLUL,GNAI1,GNAI2,GNAO1,GNAQ,GNAT2,GNA13,GNB1,GNB4,GOT1,GPD1,GPD2,GPI,GPM6A,GPR63,GPSM1,GPSM2,GPX4,GRASP,GRB2,GRIA1,GRIA2,GRIA3,GRIA4,GRID2,GRIK2,GRIK5,GRIP1,GRM1,GRM3,GSG1L,GSTP1,GTF3C1,GUCY1A2,GUCY1B1,GUCY2F,HCLS1,HDAC2,HEATR1,HGD,HGS,HNRNPK,HOMER1,HOMER2,HPCAL1,HSPA1L,HSPA2,HSPA5,HSPA8,HSPA9,HSPA12A,HSPD1,HSPE1,HSP90AA1,HSP90AB1,HTR2C,HUNK,IDH3A,IDS,IGSF22,IMMT,IMPA1,INA,IQGAP2,IQSEC1,IQSEC2,IRF7,ITM2B,ITM2C,JAK1,JAK3,KALRN,KCNAB1,KCNAB2,KCNA1,KCNA2,KCNA3,KCNA4,KCNA5,KCNJ1,KCNJ2,KCNJ4,KCNJ10,KCNJ12,KCNJ16,KERA,KHDRBS1,KIFC2,KIF1A,KIF2A,KIF3A,KIF5A,KIF13B,KLF4,KLF8,KLHDC8B,KLHL17,KMT5C,KRBA1,LANCL1,LANCL2,LAP3,LDHA,LDHB,LGI1,LRFN1,LRFN2,LRPPRC,LRP1,LRP8,LRRC1,LRRC7,LRRTM4,LSM11,MACF1,MAGI2,MAOB,MAPK1,MAPK3,MAPK14,MAP1A,MAP2,MAP3K10,MAP3K11,MARCH9,MARCH10,MASP2,MBP,MBTPS1,MDM2,MED12L,MED24,MED28,MEF2A,MIA2,MNT,MN1,MOG,MPDZ,MPP2,MSRB2,MTPN,MYCBPAP,MYL6,MYO5B,MYPN,MYRIP,NAPA,NAPB,NAPG,NBEA,NCALD,NCAM1,NCDN,NCKAP1,NCKAP5,NCKIPSD,NEDD4L,NEFH,NEFL,NEFM,NELFB,NET1,NETO1,NEUROG2,NEU4,NFATC4,NFE2,NFRKB,NKX2-1,NKX2-2,NLRP12,NMB,NME1,NOS1,NOTCH4,NOXA1,NPEPPS,NPPC,NPTN,NRCAM,NRN1,NRXN2,NSF,NSFL1C,NSUN4,NTNG2,NTRK2,NXPH3,OBSCN,OLFML3,OLFM1,OLFM2,OLFM3,OTUB1,OXCT1,PACSIN1,PARK7,PATJ,PAX6,PAX7,PCBP1,PCBP2,PCDHA13,PCDHGA7,PCDH10,PDE2A,PDE4A,PDHA1,PDHB,PDIA3,PDIA6,PDXK,PDZK1,PDZRN3,PDX1,PEBP1,PFKM,PFKP,PFN1,PGAM5,PGK1,PGK2,PGM2L1,PGR,PHACTR1,PHB,PHB2,PHF20,PHYHIP,PIKFYVE,PIK3AP1,PIK3C2A,PKM,PKP4,PLCG1,PLEKHH3,PLPP3,PLP1,PMEPA1,PMPCA,PNMA2,POLG,POU3F3,PPIA,PPIB,PPP1CA,PPP1CB,PPP1R9B,PPP1R15A,PPP2CB,PPP2R1A,PPP2R5D,PPP3CA,PPP3CB,PRAG1,PRAMEF4,PRC1,PRDM14,PRDX1,PRDX2,PRDX3,PRELP,PRG4,PRKACB,PRKAR1B,PRKAR2B,PRKCB,PRKCE,PRKN,PRRT1,PSAT1,PSMB5,PTBP1,PTBP3,PTGS1,PTK2,PTPN1,PTPN5,PTPRA,PTPRF,PTPRG,PTPRT,PTPRZ1,PYGO1,QPCTL,RAB3B,RAB3C,RAB6A,RAB10,RAB14,RAC1,RAD23A,RAD23B,RALBP1,RALGPS1,RAPGEF2,RAP1GAP,RAP2B,RARA,RASAL1,RASGRP1,RBM26,RC3H1,REPIN1,RGS9,RIDA,RIMS2,RIN1,RNPS1,ROBO2,ROCK2,RPSA,RPS3,RPS14,RPS6KA1,RPS23RG1,RSF1,RTF1,RTKN2,RUNX3,RYBP,SAP25,SCRN1,SCYL2,SDHA,SDK2,SEMA4B,SEMA4C,SEMA4G,SEMA4F,SEPTIN2,SEPTIN5,SEPTIN11,SFXN3,SF3B2,SF3B3,SHANK1,SHARPIN,SHC1,SHISA9,SH3GLB2,SH3GL2,SH3KBP1,SIM2,SIPA1L1,SIRT2,SLC1A2,SLC1A3,SLC2A1,SLC4A4,SLC6A9,SLC9A3R1,SLC17A7,SLC25A3,SLC25A4,SLC25A5,SLC25A12,SLC25A13,SLC25A22,SLC25A31,SMARCC1,SNCA,SNRPN,SNW1,SNX8,SNX22,SNX24,SOD1,SORBS1,SORBS2,SOS1,SOS2,SOX7,SPATA16,SPATA31E1,SPRR2A,SPTAN1,SPTBN1,SQSTM1,SRC,SRCAP,SRGAP3,SRRM2,SSTR1,SSTR2,SSTR4,STAP2,STIP1,STRADB,STRIP1,STXBP1,STX1B,ST13,SUCLA2,SUSD2,SYNE1,SYNE2,SYNJ1,SYNPO2L,SYP,SYTL3,TACC2,TAF1L,TAGLN3,TANC1,TBX3,TCERG1,THY1,TIE1,TJAP1,TKT,TMEM54,TMEM237,TMOD2,TNIK,TOMM40,TOX3,TP53BP2,TRAF3,TRAF6,TRIM46,TSC1,TTLL11,TTN,TTYH2,TUBA1B,TUBA1C,TUBA4A,TUBB,TUBB2A,TUBB2B,TUBB4B,TUBB6,TUFM,TXN,TXNL1,TYRO3,UBA1,UBA52,UBC,UBE2N,UBE2V1,UBE2V2,UBTF,UQCRC1,UQCRC2,USP21,USP32,USP42,UST,VAC14,VAMP3,VANGL1,VANGL2,VDAC1,VDAC2,VDAC3,VMAC,VSNL1,VWC2,VWC2L,WFDC3,WIPF1,XPO5,YTHDF1,YWHAB,YWHAE,YWHAZ,ZBTB40,ZCCHC7,ZDHHC12,ZDHHC17,ZFR,ZIC5,ZMIZ1,ZMYM3,ZNF200,ZNF207,ZNF217,ZNF622*
***DNAJC5***	Ceroid-lipofuscinosis neuronal protein 4 (Q9H3Z4)	–	*CADPS,LIN7A,LIN7C,SNAP25,STX1A,SYT1,SYT5,VAMP2*	*ATP6V1A,CADM1,CPLX1*	*AGFG1,ALG11,AP2A1,AP2B1,AP2S1,AP3D1,AP3S1,ARHGEF15,ATP6V1B1,BEST1,BTC,CASK,CD2,CFTR,CGB3,CSNK1G3,CTDSP1,DNAJC13,ECE1,EFNB1,ERC1,FANCC,FAS,FCHO1,GDI1,GRM8,HBA1,HGS,HSPA4,HSPA8,HSP90AA1,KHDC4,LCLAT1,LNPK,L3MBTL2,NECAP1,NECTIN2,NRXN3,NUFIP2,NUMB,PPFIA1,PPFIA4,PRRC2B,PSMD12,PSTPIP2,PTGFR,PTPRG,RABGEF1,RAB3A,RBSN,SCAMP1,SCAMP5,SGTA,SH3GL3,SKP2,SLC6A1,SLC17A7,SLC17A8,SLC18A3,SLC30A1,SLC30A7,SLC32A1,SNAP23,SNAP29,SNAP91,SNCA,STUB1,STXBP1,STX3,STX6,STX7,STX12,SYP,TMEM214,TRAF6,TRIM25,TTPAL,UBC,USP22,VAMP1,VAMP3,VAMP4,VAMP8,VCL,YWHAE,ZDHHC13,ZDHHC17*
***DOC2A***	Double C2-like domain-containing protein alpha (Q14183)	–	*PRRT2,SNAP25,UNC13A*	–	*ASPHD1,DOC2B,FER1L6,GDPD3,GPR137B,HIRIP3,INO80E,KCTD21,QPRT,RAB27A,RAB27B,RALYL,RAP1GDS1,SCGN,SETMAR,SEZ6L2,SNAP23,TLCD3B,TTC21B,UBASH3B,UNC13B,VDAC1,YPEL3*
***ENO2***	Neuron-specific enolase (P09104)	Enolase family	*KCNMA1*	*CUL3,PTEN*	*A2M,ACD,AKR1B1,AKR1B15,AKT1,ALDOC,ALX3,ANXA11,ARHGDIA,ASB5,ASB9,BMERB1,BPGM,CALR,CAND1,CDK2,CENPJ,COASY,CTH,CUL1,CUL2,CUL4B,C6orf47,DUT,EEF1A1,EFHD2,ENO1,ENO3,ENSA,EXOC5,EZR,FABP5,FKBP1A,FKBP1B,FKBP2,FKBP7,GABARAP,GABARAPL1,GABARAPL2,GAPDH,GMPS,GPI,GPX4,HABP4,HEXIM1,HINT1,HK1,HSD17B10,HSF1,HSPE1,H6PD,IL17B,LARP7,MAP1LC3A,MAP4,MINPP1,MSN,MTNR1A,NAT9,NDUFS7,NME2,NTRK1,PCK1,PCK2,PCMT1,PGAM1,PGAM2,PGK1,PKLR,PKM,PLS3,PNP,POC1A,POT1,PPIB,PPP1CC,PPP1R9B,PPT1,PUS7,RAB1A,RCN1,SEC31A,SIGIRR,SNUPN,SOD1,ST3GAL2,STIP1,SUMO2,SUMO3,TAGLN2,TAGLN3,TAGLN,TARDBP,TERF1,TINF2,TKT,TPD52,TPI1,TUBA4A,TXN,UBE2C,UCHL3,WDR1,YWHAE,YWHAZ*
***GRM4***	Metabotropic glutamate receptor 4 (Q14833)	–	*CAMK2A,GRM7*	*DRD2,DRD3,GRIN2A,GRIN2C,GRM2,GRM7,HTR1A*	*ACP4,ADORA1,ADRA2A,ADRA2B,AVP,CBX4,CD99L2,CHRM4,CNKSR2,CNR1,EGFR,GABBR1,GNAS,GNA12,GNA13,GRIK4,GRIP1,GRM1,GRM3,GRM8,HRH3,HTR5A,PIAS1,PIAS3,RANBP9,SDCBP,STXBP1,TAS2R4,TAS2R40,UBE2I*
***GRM7***	Metabotropic glutamate receptor 7 (Q14831)	G-protein coupled receptor 3 family	*CALM1,CAMK2A,GRM4,PDYN,PICK1,PRKCA*	*DRD2,DRD3,DRD4,KCNH2,GRM4,HTR1A*	*ADORA1,ADRA2A,CBX4,CNR1,FLNA,GABBR1,GNAQ,GNB3,GPR37L1,GRIP1,GRM3,GRM8,HRH3,HTR1B,NPY5R,OPRD1,PIAS1,PIAS3,PPP1CC,RANBP9,SDCBP,SSTR5,STXBP1,TUBA1A,UBE2I,YWHAB*
***HMGB1***	Heparin-binding protein p30 (P09429)	HMGB family	*APEX1,CTNNB1,PRKCA,PRKCG,SMARCA4*	*CTNNB1,GFAP,HDAC4,HUWE1,SMARCA2*	*ACTB,ACTC1,ACTL6A,AGER,AGR2,AGTRAP,AIFM1,ARNT,ASB1,ASB2,ATP2A1,BCAR1,BCL2,BECN1,CCDC15,CCNDBP1,CDC14B,CDK1,CDK2,CDX2,CHD4,CREBBP,CRMP1,CSNK1A1,CSNK2A1,CSNK2A2,CSNK2B,CTDP1,CXCL12,CXCR4,DDX5,DNM2,DUX4,EGFR,EP300,ERG28,ETS1,EWSR1,FAF1,FEN1,FN1,GAPDH,GTF2A1,HAVCR2,HDAC2,HEXIM1,HMGA1,HMGB1P1,HMGB2,HMGB3,HNF1A,HNRNPK,HOXB1,HOXB3,HOXC6,HOXD3,HOXD8,HOXD9,HOXD10,HOXD11,HPF1,HSD3B7,HSPA8,HTT,H1-0,H3C1,H3-3A,ICAM1,IFITM3,IKZF1,IL1B,IL6,ITGA4,KCNJ2,KIAA1429,KPNA6,LARP7,LRIF1,LY96,MAPK1,MAP1LC3B,MAP2K1,MDM2,MECP2,MLH1,MSH2,MYD88,NCAN,NFKB1,NRIP3,NR3C1,NTRK1,PARK7,PARP1,PCNA,PDIA3,PGLS,PGR,PLAT,PLCB1,PLG,POLB,POTEE,POU5F1,PPTC7,PRKCB,PRKCD,PSEN1,PTBP3,PTPRZ1,RAD23B,RAG1,RAG2,RBBP7,RB1,RBFOX3,RELA,RELB,RIF1,RNF4,SMARCB1,SMARCC1,SMARCC2,SMARCD1,SMARCD3,SMARCE1,SNCA,SOCS1,SOD1,SP100,SSRP1,SUPT16H,S100B,TBP,TCF7L2,TERF2IP,TERF2,TFAP4,TLE1,TLE5,TLR1,TLR2,TLR4,TNIP2,TP53,TP73,TUBA3C,TXN,UBA3,UBE2N,UFD1,UNC119,U2AF2,VCAM1,WDR76,XPA,XPC,XRCC5,XRCC6,YAF2,YWHAE,ZFP36,ZNF24*
***HPCA***	Neuron-specific calcium-binding protein hippocalcin (P84074)	Recoverin family	*KCNMA1,SYN2*	*SYN2*	*AP2B1,ARSA,ATP5F1B,CABP1,CREM,C1QTNF2,DGUOK,DNAAF2,DTX2,GRIA2,GRK1,HAX1,IDH3A,KCNC1,KCND3,KCNF1,MAP3K10,MBP,MLLT10,MYDGF,NAIP,DNM1,NUFIP1,PDE1A,PI4KB,PRR35,SLC16A3,SYNDIG1L,S100B,TEPSIN,TP53,TUBB4A,UQCRB,VWC2,YWHAE*
***ITPR1***	Inositol 1,4,5-trisphosphate receptor type 1 (Q14643)	InsP3 receptor family	*CALM1,CALM2,CALM3,MYO1C,NCS1*	*DISC1,TOP2B,TRPC5,TRPC6*	*ACO2,AHCYL1,AKT1,AMFR,ANK2,ATG16L1,ATP1A1,ATP1A2,ATP1A3,BANK1,BCL2,BECN1,BRCA1,CABP1,CALML3,CAMK2D,CAPZA2,CA8,CCNB1,CDK1,CRTC2,CTDNEP1,CTDP1,DCPS,EPB41L1,ERLIN1,ERLIN2,ERP44,FBXL14,FKBP1A,FUS,GRID2,GRM1,GTF2H5,HAP1,HOMER1,HOMER2,HSPA9,HTT,ITM2B,ITPR3,KCTD5,KIAA1429,LIMA1,MAD2L1,MRVI1,MYH9,MYH10,NPLOC4,ORAI1,ORAI2,PASK,PDE5A,PLCB1,PLCB4,PLCG1,PLCG2,PLCZ1,PLEC,PLK2,PPIF,PPP1R9B,PPP3CA,PRKACA,PRKACB,PRKCD,PRKG1,PRKG2,PSMD2,RAB29,RASEF,RHOA,RNF170,SCARB2,SDC2,SLC8A1,SPTAN1,SPTBN1,STARD13,STIM1,SYNPO,TAFA4,TESPA1,TRIM25,TRPC1,TRPC3,TRPC4,UBC,UBE4A,UBE4B,UFD1,VDAC1,VMP1,VCP,YWHAB,YWHAE,YWHAZ*
***KCNB1***	Potassium voltage-gated channel subfamily B member 1 (Q14721)	Potassium channel family, B subfamily, Kv2.1/KCNB1 sub-subfamily	*CALM1,SNAP25,STX1A*	*CYFIP1,KCND2,KCNH2,SCN2A*	*AMIGO1,AKAP5,FAU,HSPA1B,KCHIP2,KCNAB1,KCNAB2,KCNAB3,KCNA2,KCNB2,KCNE1,KCNE2,KCNE5,KCNG1,KCNG2,KCNG3,KCNG4,KCNH1,KCNIP2,KCNS3,KCNV1,KCNV2,NEDD4L,PRKACB,PRKACG,PRKAR1A,PRKAR1B,PRKAR2A,PRKAR2B,PTPRE,RAB40B,SRC,SUMO1,SUMO2,SUMO3,UBE2I,ZNF579*
***KCNIP3***	Calsenilin (Q9Y2W7)	Recoverin family	*CALM2,CLN3*	*CACNA1C,GRIN1,KCND2*	*ADRA2B,ASPH,CD177CREB1,CREM,CTBP1,CTBP2,DPP6,DPP10,FYN,GRK2,GRK6,HNRNPL,IGF1R,IGLV3-25,IL6ST,KCNA4,KCNC1,KCND1,KCND3,KCNK1,MAVS,OCIAD2,PCBD2,PPP3CA,PSEN1,PSEN2,SOD3,SRL,SUMO1,TARBP2,TEAD3,UBE2I,VAMP5,ZG16*
***KCNMA1***	Calcium-activated potassium channel subunit alpha-1 (Q12791)	–	*CACNA1A, CACNA1B,CALM1,CALM2,CALM3,ENO2,HPCA,LIN7C,STX1A,SYN1,SYN2*	*ATP1B1,CACNA1C,FMR1,GFAP,MAP6,SYN2,YWHAG*	*ABCE1,ACAA2,ACO2,ACTA1,ACTA2,ACTB,ACTG1,ACTG2,ACTR1A,ACTR1B,ACTR3,ADRB2,AHSG,AIF1L,AKAP5,AK1,ALAD,ALB,ALDH6A1,ALDOA,ALDOC,AMPD2,ANP32A,ANXA3,ANXA5,ANXA7,APOA1,APOE,APOH,APRT,ARHGDIB,ASIC1,ATP1A1,ATP1A2,ATP1A3,ATP5F1A,ATP5F1B,ATP5F1A,ATP5F1B,ATP6V0D1,CADM3,CALB2,CALR,CAPG,CAV1,CAV2,CAV3,CA2,CBX1,CCSAP,CCT2,CEP290,CFL1,CIRBP,CKB,CKM,CLTA,CMPK1,COL1A1,COL1A2,CRBN,CRKL,CRYAB,CTBP1,CTTN,CUL1,CYFIP2,DDAH1,DDB1,DDX1,DFFA,DLD,DNAJA2,DNM1,DPYSL2,ECPAS,EED,EEF1A1,EIF3F,EIF4A2,ENO1,ENO3,EPB41L3,ERLIN2,ETFB,FADD,FBXO7,FGB,FH,GAPDH,GC,GDI1,GLO1,GLUD1,GPD1,GOT1,GPX1,GRAP2,GSTM1,HBB,HEBP1,HNRNPA1,HNRNPH2,HNRNPK,HNRNPL,HPCAL1,HPX,HSPA1B,HSPA1L,HSPA2,HSPA5,HSPA8,HSPA9,HSPD1,IDH3A,IDI1,ID1,INA,INMT,INPP1,ISYNA1,KCNMB1,KCNMB2,KCNMB3,KCNMB4,KCNN1,KCNN2,KCNN3,KCNN4,KHDRBS1,KIAA1107,KIF5C,KLC2,KNG1,LCP1,LDHA,LDHB,LGALS1,LMNA,LRPAP1,LRRC26,MAP1A,MBP,MDH2,METTL7B,MGST1,MPZ,MTAP,MYH1,MYLPF,NAP1L4,NDRG1,NEFH,NEFL,NEFM,NUCB1,NUDC,NUDT4,OAT,PARK7,PA2G4,PCNA,PDHB,PDIA3,PDXK,PEBP1,PFKP,PGAM1,PGK1,PGRMC2,PHGDH,PHYHIP,PIK3C3,PIK3R4,PLA2G4A,PPIA,PRDX2,PRKACB,PRKG1,PRKG2,PRPH,PRX,PSMA7,PSMD11,PTPRA,PURA,PZP,RAB1A,RAB11B,RAP1A,RBM4B,RCN3,RHOA,RNPEP,RPL12,RPL22,RPS7,RPSA,RPS12,RPS14,RPS19,RPS20,RPS21,RUVBL1,SDHB,SERBP1,SERPINA1,SERPINB6,SH3GL1,SKP1,SLC34A3,SOD1,SOD2,SPARC,SPR,STIP1,STK26,STMN1,STXBP1,SUCLA2,SUCLG2,ST8SIA2,TAGLN,TALDO1,TBRG4,TBXA2R,TF,TKT,TNIK,TNK2,TPI1,TPM1,TPM2,TPT1,TP53,TUBA1A,TUBA1B,TUBA4A,TUBB,TUBG1,TUBGCP2,TUBGCP3,TUBGCP4,UBC,UCHL1,UQCRC1,VAT1,VCP,VDAC1,VIM,YWHAB,YWHAE,YWHAH,YWHAQ,YWHAZ*
***KIF1B***	Kinesin-like protein KIF1B (O60333)	TRAFAC class myosin-kinesin ATPase superfamily, Kinesin family, Unc-104 subfamily	*DLG4*	*DLG4,FMR1*	*APP,BACH1,BCAR1,BICD2,BIN2,BRCA1,BVLF1,CANX,CAV3,CBY1,CERS2,CETN1,CGN,CLASP1,CLIP4,CLK1,CPEB1,CSNK1A1,CSNK2A2,C18orf25,DENND1A,DLG1,DLG2,DYSF,EEF1A1,EIF4A1,EIF4E2,ELAVL1,FANCG,GABBR1,GIGYF1,GIPC1,GOPC,HNRNPL,HSPA8,JAKMIP1,KCTD3,KIFAP3,KIFBP,KIF1A,KIF4A,KIF5A,KIF5B,KIF13B,KIF21A,KIF21B,KLC1,KLC2,KLC3,KLC4,KSR1,LAMTOR5,LRFN1,MAGEA1,MAGI1,MAGI2,MAST3,MUS81,NAV1,NCOA4,NINL,PARD6A,PARK7,PCBP1,PDF,PGM1,PIK3C3,PLOD1,POMP,PPP4R1,PSMC3,PTPRE,RAD50,RBM7,RTKN,SEC24A,SH3PXD2A,SIAH1,SMC1A,SMC6,SRGAP2,STARD13,TFCP2,THOC2,TMED2,TRAK2,TRIM55,TUBG1,TULP2,UBE2I,VIPAS39,VIRMA,VPS26B,VPS33B,YPEL5,YWHAB,YWHAE,YWHAH,YWHAQ,YWHAZ,ZBTB21,ZNF217,ZSCAN26*
***LAMA1***	S-laminin subunit alpha (P25391)	–	*CALM1,CHP1,DAG1*	–	*ACHE,ANOS1,AP4E1,ATL2,BAG2,BAG3,CBX4,COL18A1,CRP,DCTN4,DNAJC7,EED,FBLN2,GET4,HGF,HIST1H2BH,HSPA5,HSPG2,IMMT,ITGA1,ITGA2,ITGA3,ITGB1,KCTD5,KEAP1,KNG1,KSR2,LAMB1,LAMB2,LAMB3,LAMC1,LGALS3,LGALS9,LOXL3,L1TD1,MAPK9,MEP1A,MYH3,NES,NID1,NID2,OS9,PLAT,PLG,POU5F1,PRKCI,PRMT6,PYHIN1,RNF123,STRN3,SUCO,TMPO,TOMM40,TRIM32,TUBE1,TUBG1,TUBG1,UBC,VPS50,YWHAE,ZNF408*
***LIN7A***	Protein lin-7 homolog A (O14910)	Lin-7 family	*DLG4,DNAJC5,LIN7B,LIN7C,NRXN1,SNAP25,SNTB2,STX1A,SYT1*	*APP,CADM1,DLG4,GRIN2B,NRXN1,SYN3*	*ABCA1,ADGRB1,ADRA1D,AMOT,APBA1,CASK,CASKIN1,CDA,CHRD,DLG1,DLG3,ECM1,EPB41L2,FAM9B,GABARAPL2,GLYR1,GOLGA2,GUCY1A2,GUCY1B1,KCNJ4,KCNJ12,KIF17,MARCH2,MARCH11,MDFI,MINDY3,MPDZ,MPP2,MPP3,MPP5,MPP6,MPP7,NOTCH2NLA,NRXN2,NSUN2,PATJ,PIP4K2B,PPFIA1,PPFIA2,PPFIA3,PPFIA4,PXDC1,SLC6A12,SLC20A1,TAX1BP3,UBC,YWHAB,YWHAE,ZYX*
***LIN7B***	Protein lin-7 homolog B (Q9HAP6)	Lin-7 family	*CALM1,CALM2,CALM3,CTNNB1,DLG4,LIN7A,LIN7C,SNAP25,STX1A,SYT1*	*APC,CPLX1,CTNNB1,DLG4,GRIN1,GRIN2B*	*ABCA1,APBA1,ARHGEF26,ASIC3,BAIAP2,CASK,CASKIN1,DLG1,ENKD1,FOPNL,KCNJ4,KCNJ12,KIF26B,MOV10,MPDZ,MPP2,MPP3,MPP5,MPP6,MPP7,NRXN3,PATJ,POLE4,PPFIA2,PPFIA3,PPFIA4,PSMB1,PXDC1,RHOA,RTKN,TCEANC,TRAPPC12,TSPOAP1,WDYHV1,YWHAB,YWHAE,YWHAZ*
***LIN7C***	Protein lin-7 homolog C (Q9NUP9)	Lin-7 family	*CALM1,CALM2,CALM3,DNAJC5,KCNMA1,LIN7A,LIN7B,NRXN1,SNTB2*	*APC,CPLX1,GRIN2B,NRXN1,XPO1*	*ABCA1,ABLIM1,ABLIM2,AMOT,AMOTL1,AMOTL2,APBA1,ARHGEF26,CAPZA2,CASK,CASKIN1,CCDC32,CFTR,COPS5,CYSLTR2,DLG1,DPCD,ELAVL1,ESR1,ESR2,EZR,FLNB,FOXRED2,GNAI2,HERC4,HTR2C,KCNJ2,KCNJ4,KCNJ12,KIF26B,LAMP1,LATS2,LIMA1,MARCH11,MGST3,MPDZ,MPP2,MPP3,MPP5,MPP6,MPP7,NET1,NF2,NONO,NSUN2,NUP37,PATJ,PLEKHG5,PPFIA1,PPFIA2,PPFIA3,PPFIA4,PPP1CC,PXDC1,PXDC1,RAD18,SFPQ,SLC2A4,SLC20A1,SLC25A41,SPATA2,TGOLN2,TMEM17,TSPOAP1,TUBA1A,UIMC1,UNC13B,WDTC1,WWC1,WWTR1,YAP1,YES1,ZMYM1*
***LRP2***	Low-density lipoprotein receptor-related protein 2 (P98164)	LDLR family	*CTNNB1,DLG4,SHH,SYT1*	*APP,CTNNB1,DLG4,NOS1AP,SCN3A*	*ABL1,ADAMTS1,AGRN,ALB,AMBN,AMN,ANAPC10,ANG,ANKRA2,ANKRD36B,ANKS1B,APBA2,APOA1,APOB,APOE,APOH,APBB1,APPL1,ARHGAP19,ATN1,CALCA,CBX5,CCDC59,CCN2,CDC42,CLCN5,CLU,CTSB,CTSD,CUBN,CXCL16,DAB1,DAB2,DEFB123,DKK2,DLG2,DLG3DLK2,DYNC1I2,EEA1,EGFL6,FEZF1,FGF17,FGFRL1,GAS1,GC,GCG,GIPC1,GREM2,HSD17B10,IL10,IL20,INSL6,ITGB1BP1,LDLRAP1,LPA,LPL,LRPAP1,LRP2BP,MAGI1,MANSC1,MAP7,MAPK8IP1,MAPK8IP2,MEPE,M6PR,OBP2A,OCRL,PDGFD,PLA2G5,PLAU,PMCH,PPIB,PRSS23,PTCH1,RAC1,RBP1,RHOA,RIBC2,RNASE13,RNF123,RPGRIP1L,SCGB1A1,SLC9A3,SNW1,SNX17,SOST,SYNJ2BP,SYT2,TAFA2,TAFA3,TAFA4,TG,THBS1,TMEM17,TNFAIP6,UBC,UTP14C,VDAC2,VIP,YWHAZ,ZBTB48,ZFP41,ZNF224,ZNF264,ZNF408,ZNF517,ZSCAN29*
***LRP6***	Low-density lipoprotein receptor-related protein 6 (O75581)	LDLR family	*CDH2,CTNNB1,LRRK2,MDK*	*CTNNB1,DKK1,GRIN2A,KREMEN1,WNT1*	*ADAM33,AMMECR1,ANGPTL4,ANTXR1,ANTXR2,APOB,ATP13A2,AXIN1,BAMBI,CAPRIN2,CAV1,CHST9,CKLF,CMA1,COPS2,CSNK1A1,DAB2,DKKL1,DKK2,DVL1,DVL2,DVL3,EEA1,EPN1,ETV6,FANCD2,FCGRT,FZD1,FZD2,FZD5,FZD8,GAS8,GPATCH2,GSK3A,GSK3B,IGFBP1,IGFBP2,IGFBP4,IGFBP6,IQGAP1,ITCH,KLK5,KREMEN2,LGR4,LGR5,LRP5,MESD,MGAT4C,NPC1,OVOL2,PARP16,PDGFRB,POMK,PRSS37,PTH,PTH1R,PTPRH,RAB11A,RASD2,RASSF1,RIPK4,SCGB1D1,SDC1,SDC4,SERPINF1,SOST,ST8SIA4,SYT9,TAFA2,TENT4A,TGIF2LY,TMEM17,VIRMA,WNT3A,WNT5A,WNT9B,WNT10B,ZKSCAN8,ZNRF3*
***LRRK2***	Leucine-rich repeat serine/threonine-protein kinase 2 (Q5S007)	Protein kinase superfamily, TKL Ser/Thr protein kinase family	*APEX1,ATP2B2,CACYBP,CADPS,CALM1,CALM2,CALM3,CTNNB1,LRP6,MYO1C,MYO1D,MYO5A,PAFAH1B1,PAK1,PRKN,SMARCA4,SNAPIN,SNAP25,STAC,STX1A,SV2A,SYN1,TPM3,VAMP2*	*ATP2A2,ATP6V1A,CPLX1,CTNNB1,CUL3,CYFIP1,HERC2,HUWE1,PAFAH1B1,FMR1,RPTOR,SHMT2,XPO1,YWHAG*	*AAMP,AARS1,AARS2,AASDHPPT,ABCB7,ABCE1,ABCF1,ABCF2,ABCF3,ABHD10,ABLIM1,ABL1,ABL2,ACACA,ACADM,ACADSB,ACAD9,ACIN1,ACLY,ACOX1,ACOT7,ACOT9,ACO1,ACO2,ACSL3,ACSL4,ACSL5,ACTA1,ACTA2,ACTB,ACTBL2,ACTC1,ACTG1,ACTG2,ACTL6A,ACTN1,ACTN4,ACTR1A,ACTR1B,ACTR2,ACTR3,ADRM1,ADSS2,AFDN,AFF4,AFG3L2,AGAP1,AGK,AGL,AGO1,AGO2,AGPS,AHCY,AHCYL1,AHDC1,AHNAK,AHSA1,AIFM1,AIMP1,AIMP2,AKAP8,AKR1B1,AKR7A2,AKT1,AKT2,AK1,AK2,AK4,AK6,ALB,ALDH1B1,ALDH2,ALDH5A1,ALDH7A1,ALDH8A1,ALDH9A1,ALDH18A1,ALDOA,ALOX12B,ALYREF,AMOT,ANAPC1,ANKFY1,ANKRD17,ANKS4B,ANP32B,ANP32E,ANXA2,APEH,APEX2,API5,APOA1,APOA2,APOA4,APOC3,APRT,AP1B1,AP1G2,AP2A1,AP2A2,AP2B1,AP2M1,AP3B1,AP3D1,AP3M1,ARCN1,ARFGAP1,ARF1,ARF3,ARF4,ARF5,ARF6,ARG1,ARHGAP15,ARHGDIA,ARHGEF7,ARL1,ARMT1,ARPC1A,ARPC1B,ARPC2,ARPC4,ARPC5,ASCC3,ASNS,ATAD1,ATAD3A,ATAD3B,ATG3,ATP1A1,ATP1A2,ATP1A3,ATP5F1A,ATP5F1B,ATP5F1C,ATP5ME,ATP5MG,ATP5PO,ATP6V0A1,ATP6V1B2,ATP6V1E1,ATP13A1,ATRX,ATXN2,ATXN2L,ATXN7L3B,ATXN10,AURKB,AURKC,AXIN1,AZGP1,BAG1,BAG2,BAG3,BAG5,BAG6,BAX,BCAS2,BCCIP,BCKDK,BCLAF1,BCL2,BCR,BICD2,BLMH,BOLA2,BOP1,BPNT1,BRCA2,BRD2,BRIX1,BUB3,BYSL,BZW1,CAD,CALB2,CALML5,CALR,CAMK1D,CAMK2G,CANX,CAPN1,CAPRIN1,CAPZA1,CAPZA2,CAPZB,CAP1,CARM1,CASP3,CASP8,CASP14,CA2,CBFB,CBLB,CBR1,CBSL,CBX3,CCAR1,CCDC6,CCDC43,CCDC47,CCL21,CCNA2,CCT2,CCT3,CCT4,CCT5,CCT8,CDC23,CDC25A,CDC27,CDC37,CDC42,CDC42EP3,CDC45,CDC73,CDIPT,CDKL3,CDKN2A,CDK1,CDK2,CDK4,CDK5,CDK6,CDK9,CDK11B,CDK12,CDSN,CD2BP2,CELF1,CENPF,CEP72,CEP170,CETN3,CFAP20,CFAP65,CFL1,CHCHD3,CHD1,CHD1L,CHD4,CHERP,CHGB,CHORDC1,CHTOP,CIAO1,CIAO2B,CIP2A,CKAP2,CKAP4,CKAP5,CKB,CKM,CKMT1A,CLASP2,CLIC1,CLIC4,CLNS1A,CLN6,CLPB,CLPP,CLPX,CLTA,CLTB,CLTC,CLU,CLUH,CMAS,CMPK1,CMTR1,CNBP,CNN3,CNOT1,COASY,COIL,COLGALT1,COMMD2,COPA,COPB1,COPB2,COPE,COPG1,COPG2,COPS3,COPS6,COPZ1,COQ8A,COX6C,CPNE3,CPOX,CPSF6,CPSF7,CPTP,CRABP2,CRKL,CS,CSDE1,CSE1L,CSNK1A1,CSNK1D,CSNK1E,CSNK1G1,CSNK1G2,CSNK1G3,CSNK2A1,CSNK2A2,CSNK2B,CSRP2,CSTB,CSTF1,CSTF3,CTBP1,CTNNA1,CTNNBL1,CTNND1,CTPS1,CTSD,CTTN,CUEDC1,CUL1,CUL2,CUL4B,CUL5,CXCL11,CXorf56,CWC15,CYB5R3,CYCS,CYC1,CYFIP2,CYP51A1,CYREN,C1orf87,C1QBP,C12orf29,C17orf85,C18orf25,DAD1,DAPK1,DAPP1,DAP3,DAZAP1,DBF4B,DBN1,DBR1,DCAF7,DCAKD,DCD,DCTN1,DCTN4,DCXR,DDAH2,DDB1,DDIT4,DDOST,DDX1,DDX3X,DDX5,DDX6,DDX19B,DDX20,DDX21,DDX23,DDX39A,DDX39B,DDX42,DDX46,DDX47,DDX50,DDX54,DECR1,DEK,DFFA,DFFB,DHCR7,DHCR24,DHFR,DHRS7B,DHX9,DHX15,DHX16,DHX29,DHX30,DHX36,DHX40,DHX57,DIAPH1,DIDO1,DIS3,DKC1,DLAT,DLD,DLL1,DLST,DNAAF5,DNAJA1,DNAJA2,DNAJA3,DNAJB1,DNAJB6,DNAJB8,DNAJC7,DNAJC9,DNMT1,DNM1,DNM1L,DNM2,DNM3,DOCK7,DPH5,DPM1,DPM3,DPP3,DPP9,DPYSL2,DPYSL3,DRG1,DRG2,DSC1,DSC3,DSG1,DSP,DSTN,DTYMK,DUX3,DVL1,DVL2,DVL3,DYNC1H1,DYNC1LI1,DYNLL1,DYRK2,EARS2,EBP,ECHDC1,ECHS1,ECI1,ECI2,ECPAS,EDC3,EDC4,EEF1AKNMT,EEF1A1,EEF1A2,EEF1B2,EEF1D,EEF1E1,EEF1G,EEF2,EFHD2,EFTUD2,EGFR,EHD4,EIF1AX,EIF2A,EIF2AK2,EIF2B1,EIF2B2,EIF2B3,EIF2B4,EIF2B5,EIF2S1,EIF2S2,EIF2S3,EIF3A,EIF3B,EIF3CL,EIF3D,EIF3E,EIF3F,EIF3G,EIF3H,EIF3I,EIF3J,EIF3K,EIF3L,EIF3M,EIF4A2,EIF4A3,EIF4B,EIF4E,EIF4EBP1,EIF4G1,EIF5,EIF5A,EIF5B,ELAC2,ELAVL1,ELOC,ELP1,ELP2,EMC1,EMD,EML4,ENAH,ENKUR,ENO1,EOGT,EPB41,EPB41L2,EPHX1,EPRS1,EPS8L2,ERG,ERLIN1,ERLIN2,ERO1A,ERP29,ERP44,ESD,ESRRG,ESYT1,ETFA,ETFB,ETF1,ETV5,EWSR1,EXOC2,EXOSC4,EXOSC9,EXOSC10,EYA3,E2F4,FAAP24,FADD,FADS1,FAF2,FAM27E3,FAM47B,FAM50A,FAM90A1,FAM98A,FAM98B,FAM107A,FAM184A,FANCI,FANCM,FARSA,FARSB,FASN,FASTKD1,FASTKD2,FAU,FBL,FCHSD1,FDFT1,FDPS,FEN1,FERMT2,FH,FHOD1,FIS1,FKBP4,FLG,FLG2,FLII,FLNA,FLNB,FLNC,FLOT2,FRG1,FSCN1,FTSJ3,FUBP3,FUTSCH,FXR1,FXR2,FZD5,GAK,GALK1,GAPDH,GAPVD1,GARS1,GATAD2A,GCAT,GCDH,GCLM,GCN1,GDI1,GDI2,GEMIN4,GEMIN5,GEMIN8,GFM1,GFPT1,GGCT,GIGYF2,GIMAP8,GLMN,GLUD1,GMPPA,GMPS,GNAI2,GNAI3,GNAO1,GNAS,GNA12,GNA13,GNB2,GNE,GNL2,GNL3,GNL3L,GOLGA2,GOLGB1,GORASP2,GOT2,GPBP1L1,GPNMB,GPS1,GRHPR,GRPEL1,GSDMA,GSK3A,GSK3B,GSTM2,GSTM3,GSTP1,GTF2B,GTF2H2,GTF2I,GTF3C1,GTF3C3,GTF3C4,GTF3C5,GTPBP1,GTPBP4,GTSF1,G3BP1,G3BP2,HACD3,HADH,HADHB,HAL,HARS1,HAT1,HCFC1,HDAC1,HDAC2,HDAC6,HDGFL2,HDHD5,HDLBP,HEATR3,HECTD1,HELLS,HIBCH,HIC1,HIF1A,HINT1,HIST1H2BB,HIST2H2AA3,HK1,HK2,HLA-C,HMGCS1,HMGN2,HMMR,HM13,HNRNPA2B1,HNRNPA0,HNRNPA3,HNRNPD,HNRNPF,HNRNPH1,HNRNPH2,HNRNPH3,HNRNPL,HNRNPM,HNRNPR,HNRNPU,HNRPA1,HOGA1,HPRT1,HPX,HRNR,HSDL2,HSD17B4,HSD17B10,HSD17B11,HSPA1A,HSPA1B,HSPA4,HSPA4L,HSPA5,HSPA8,HSPA9,HSPBP1,HSPB1,HSPD1,HSPE1,HSPH1,HSP90AA1,HSP90AB1,HSP90B1,HTATSF1,HTT,HYOU1,H1-3,H3C1,H3-3A,H3-3B,H4C1,IARS2,IDE,IDH1,IDH2,IDH3A,IDH3B,IDH3G,IDI1,IGF2BP1,IGF2BP2,IGF2BP3,IGHA1,IGHG1,IGKC,IGLC6,IMMT,IMPDH2,INTS13,IPO5,IPO7,IPO8,IPO9,IPO11,ITIH4,IQGAP1,IQGAP3,IRAK1BP1,IRS1,IRS4,ITCH,ITGB3BP,ITIH2,ITPA,IVD,JHY,JUP,KARS1,KCTD12,KCTD18,KDELR1,KDM4D,KHDRBS1,KHSRP,KIFBP,KIF2A,KIF5B,KIF21A,KLC2,KNG1,KNTC1,KPNA3,KPNA4,KPNB1,KPRP,KRT1,KRT2,KRT5,KRT6A,KRT6B,KRT8,KRT9,KRT10,KRT14,KRT16,KRT17,KRT23,KRT73,KRT77,KRT78,KRT80,KTN1,LAMP2,LARP1,LARP4,LARP7,LARS2,LAS1L,LATS1,LBR,LDHA,LDHB,LDHAL6A,LETM1,LGALS8,LIG3,LIMA1,LIMS2,LMAN1,LMF2,LMNA,LMNB1,LMNB2,LOC51064,LONP1,LORICRIN,LPCAT1,LRBA,LRPPRC,LRRC40,LRRC47,LRRC59,LRRK1,LRSAM1,LSM12,LTV1,LUC7L2,LUC7L3,LYPLAL1,LYPLA2,LYZ,L3MBTL3,MACROD1,MAD1L1,MAD2L1,MAGED2,MAGT1,MAIP1,MAPK1,MAPK3,MAPK8IP1,MAPK8IP2,MAPK8IP3,MAPRE1,MAPRE2,MAPT,MAP1A,MAP1B,MAP2,MAP2K1,MAP2K2,MAP2K3,MAP2K4,MAP2K6,MAP2K7,MAP4,MAP4K4,MARK2,MATK,MATR3,MAT2A,MAZ,MBP,MCCC2,MCMBP,MCM2,MCM3,MCM4,MCM5,MCM6,MCM7,MDH1,MDH2,MDN1,MEF2D,METAP1,METAP2,METTL2B,ME2,MFN1,MFN2,MGST1,MICOS13,MID1,MIF,MIPEP,MKI67,MKNK2,MLLT3,MMS19,MMTAG2,MOB1B,MOGS,MOV10,MPC2,MPP6,MPRIP,MRGBP,MRI1,MRNIP,MRPL2,MRPL4,MRPL9,MRPL10,MRPL13,MRPL14,MRPL16,MRPL19,MRPL20,MRPL21,MRPL22,MRPL23,MRPL27,MRPL28,MRPL37,MRPL38,MRPL39,MRPL43,MRPL44,MRPL47,MRPL58,MRPS2,MRPS5,MRPS6,MRPS7,MRPS9,MRPS10,MRPS12,MRPS16,MRPS17,MRPS18A,MRPS18B,MRPS21,MRPS22,MRPS25,MRPS26,MRPS27,MRPS28,MRPS31,MRPS34,MRPS35,MRTO4,MSH2,MSH5,MSH6,MSI1,MSN,MSTO1,MTA1,MTA2,MTCH2,MTDH,MTHFD1,MTHFD1L,MTHFD2,MTOR,MTREX,MTX3,MT-ATP6,MYBBP1A,MYDGF,MYH2,MYH9,MYH10,MYH14,MYL1,MYL6,MYL9,MYL12B,MYO1B,MYO1F,MYO6,NAA10,NAA15,NAA50,NACA,NAE1,NAF1,NAMPT,NAPB,NAP1L1,NAP1L4,NARS1,NASP,NAT1,NAT10,NCAPD3,NCAPG,NCBP1,NCBP3,NCCRP1,NCDN,NCKAP1,NCL,NCLN,NDUFAF3,NDUFAF7,NDUFA4,NDUFA5,NDUFA6,NDUFA9,NDUFA10,NDUFA13,NDUFB4,NDUFB9,NDUFS1,NDUFS2,NDUFS3,NDUFS7,NDUFS8,NDUFV1,NEFL,NEFM,NEK1,NEK4,NEK6,NEK9,NELFB,NELFCD,NEMF,NEURL4,NFATC2,NHLRC2,NIBAN2,NIPSNAP1,NIPSNAP2,NKAP,NKRF,NMD3,NME1,NME2,NME4,NME7,NMT1,NNT,NOC4L,NOLC1,NOMO1,NOMO2,NONO,NOP58,NOSIP,NPEPPS,NPLOC4,NPM1,NPPC,NSDHL,NSF,NSL1,NSUN2,NTMT1,NTPCR,NT5DC1,NT5DC2,NUB1,NUDC,NUDCD1,NUDT2,NUDT19,NUDT21,NUMA1,NUP50,NUP62,NUP88,NUP93,NUP98,NUP107,NUP133,NUP153,NUP155,NUP160,NUP188,NUP205,NUTF2,NXF1,NXN,OAT,OBI1,OCRL,OGA,OGDH,OGT,OLA1,OPA1,ORC3,OSBP,OSBPL9,OSTC,OTUB2,OTUD4,OXA1L,OXSR1,PABPC1,PABPC4,PADI4,PAFAH1B2,PAFAH1B3,PAF1,PAICS,PAK6,PAM16,PAPSS1,PARG,PARK7,PARP1,PA2G4,PBDC1,PBK,PCBP1,PCBP2,PCK2,PCMT1,PCM1,PCNA,PCNP,PCSK1N,PDCD4,PDCD6IP,PDCL,PDE12,PDHA1,PDHB,PDIA3,PDIA4,PDIA6,PDS5A,PDXP,PEBP1,PELO,PES1,PFAS,PFKL,PFKM,PFKP,PFN1,PFN2,PGAM5,PGD,PGK1,PGLS,PGM1,PGM2,PGM3,PGP,PHB,PHB2,PHF6,PHF20L1,PHGDH,PHP14,PIAS1,PIGT,PIK3R1,PINK1,PIP5K1A,PISD,PITPNB,PITRM1,PKD1,PKM,PKN2,PKP1,PLEC,PLK1,PLOD1,PLP1,PLS1,PLS3,PML,PMPCA,PMVK,POFUT1,POF1B,POLA1,POLDIP3,POLD1,POLD2,POLE,POLRMT,POLR1C,POLR2B,POLR2H,POLR2L,POLR2M,POLR3A,POP1,POU5F1,PPAN,PPAT,PPA1,PPA2,PPFIA1,PPIB,PPIF,PPIH,PPIL1,PPIL4,PPME1,PPP1CA,PPP1CB,PPP1CC,PPP1R7,PPP1R8,PPP1R12A,PPP2R1A,PPP2R2A,PPP3CA,PPP4C,PPP4R3A,PPP6C,PPP6R3,PRDX1,PRDX2,PRDX3,PRDX4,PRDX6,PREPL,PRKACA,PRKAR1A,PRKAR2A,PRKAR2B,PRKCI,PRKCZ,PRKDC,PRKN,PRKRA,PRMT1,PRMT3,PRMT5,PRPF6,PRPF8,PRPF19,PRPF31,PRPF40A,PRPSAP2,PRPS1,PRRC2C,PRTFDC1,PSMA1,PSMA2,PSMA3,PSMA4,PSMA6,PSMA7,PSMB1,PSMB2,PSMB3,PSMB4,PSMB5,PSMB6,PSMC3,PSMC4,PSMC5,PSMC6,PSMD1,PSMD2,PSMD3,PSMD4,PSMD5,PSMD6,PSMD8,PSMD10,PSMD11,PSMD12,PSMD13,PSMD14,PSME2,PSME3,PSME3IP1,PSMG1,PSPC1,PTBP1,PTCD3,PTGES3,PTGS2,PTK2,PTK6,PTPN1,PTPN11,PTPN23,PTP4A1,PTRH2,PUF60,PUM1,PYCR1,PYCR2,PYCR3,PYGB,PYGL,PYM1,P2RY11,P4HB,QARS,QPCTL,QRICH1,QRSL1,RABGGTB,RAB1A,RAB1B,RAB3A,RAB3C,RAB3GAP2,RAB5A,RAB5B,RAB5C,RAB7A,RAB8A,RAB10,RAB11A,RAB11B,RAB11FIP2,RAB12,RAB13,RAB14,RAB21,RAB29,RAB32,RAB35,RAB38,RACK1,RAC1,RAD21,RAD50,RAD51AP1,RAI14,RALYL,RAN,RANBP1,RANBP2,RANGAP1,RAPGEF4,RAP1B,RARS1,RASL11B,RAVER1,RBBP4,RBBP6,RBBP7,RBBP8,RBIS,RBMS1,RBM12,RBM14,RBM17,RBM22,RBM25,RBM26,RBM39,RBM42,RCC1,RCC2,RCN2,RDX,RECQL,REPS1,RFC1,RFC2,RFC3,RFC4,RGS1,RGS2,RHBDD1,RHNO1,RHOA,RHOT1,RIC8A,RIF1,RING1,RIOK1,RIOX2,RIPK1,RIPK2,RIT1,RNF20,RNF40,RNPEP,RNPS1,ROCK2,RO60,RPAP3,RPA1,RPA3,RPLP0,RPLP1,RPLP2,RPL3,RPL5,RPL7,RPL7A,RPL8,RPL9,RPL10,RPL10A,RPL11,RPL12,RPL13,RPL13A,RPL14,RPL15,RPL17,RPL18,RPL18A,RPL19,RPL21,RPL22,RPL22L1,RPL23,RPL23A,RPL24,RPL26,RPL27,RPL27A,RPL28,RPL30,RPL31,RPL32,RPL34,RPL35,RPL35A,RPL36,RPL36A,RPL36AL,RPL37A,RPL38,RPL39,RPN1,RPP30,RPP38,RPSA,RPS2,RPS3,RPS3A,RPS4X,RPS4Y1,RPS5,RPS6,RPS6KA3,RPS6KB2,RPS7,RPS8,RPS9,RPS10,RPS11,RPS12,RPS13,RPS14,RPS15,RPS15A,RPS16,RPS17,RPS18,RPS19,RPS20,RPS21,RPS23,RPS24,RPS25,RPS26,RPS27,RPS27A,RPS28,RPS29,RRBP1,RRM1,RRP1,RTCB,RTRAF,RUFY1,RUVBL1,RUVBL2,SAAL1,SACM1L,SAE1,SAFB,SAFB2,SAMHD1,SART3,SBDS,SBSN,SCAMP3,SCCPDH,SCEL,SCFD1,SCO2,SCRIB,SCYL2,SDAD1,SDHA,SDHB,SDHC,SEC11A,SEC16A,SEC22B,SEC23B,SEC24A,SEC24C,SEC31A,SEC61A1,SENP3,SEPTIN2,SEPTIN3,SEPTIN5,SEPTIN7,SEPTIN8,SEPTIN9,SEPTIN11,SERBP1,SERF1A,SERPINB6,SERPINB12,SERPINH1,SESN2,SETSIP,SFN,SFPQ,SFRS2,SFRS3,SFRS4,SFRS5,SFXN1,SFXN4,SF1,SF3A1,SF3B1,SF3B2,SF3B3,SF3B5,SF3B6,SGPL1,SH3GLB2,SH3GL1,SH3GL2,SH3GL3,SKA3,SLC1A5,SLC3A2,SLC7A5,SLC12A2,SLC16A1,SLC25A1,SLC25A3,SLC25A4,SLC25A5,SLC25A6,SLC25A10,SLC25A11,SLC25A22,SLC30A7,SLC37A4,SLFN5,SLIRP,SLK,SMAP1,SMARCA5,SMARCB1,SMARCC1,SMC1A,SMC2,SMC4,SMN1,SMPD4,SMS,SMTNL2,SMU1,SNAP91,SNCA,SNCB,SNRNP40,SNRNP70,SNRNP200,SNRPA1,SNRPB2,SNRPD1,SNRPD2,SNRPD3,SNRPE,SNX3,SNX9,SNX27,SON,SORD,SPAG9,SPART,SPATA24,SPATS2,SPCS2,SPCS3,SPRR1B,SPTAN1,SPTBN1,SPTLC1,SPIN1,SP100,SQSTM1,SRM,SRPK1,SRPRA,SRPRB,SRP9,SRP14,SRP54,SRP68,SRRM1,SRSF1,SRSF7,SRSF11,SSB,SSBP1,SSRP1,SSR1,SSR4,STAG2,STAT1,STIP1,STK3,STK24,STK25,STK33,STK40,STMN1,STN1,STOML2,STON2,STRAP,STRBP,STT3A,STT3B,STUB1,STXBP1,STXBP3,STX1B,SUCLA2,SUCLG1,SUCLG2,SUDS3,SUMO2,SUPT4H1,SUPT6H,SUPT16H,SUPV3L1,SURF4,SYNJ1,S100A8,TACO1,TADA2B,TAF4,TAF7L,TAGLN2,TAOK1,TAOK3,TARDBP,TARS1,TARS2,TAS2R60,TBCB,TBCD,TBCE,TBC1D4,TBC1D22B,TBL3,TCAF1,TCEA2,TCERG1,TCF25,TCOF1,TCP1,TCP10L,TECR,TELO2,TES,TEX10,TEX33,TFB2M,TFG,TGIF2LX,TGM3,TGOLN2,THOC1,THOC2,THOC3,THOP1,THRAP3,THUMPD3,TIGAR,TIMM13,TIMM21,TIMM23B,TIMM29,TIMM44,TIMM50,TIPRL,TKT,TK1,TLE4,TLN1,TMCO1,TMEM11,TMEM33,TMEM97,TMEM189,TMEM263,TMOD3,TMPO,TMPRSS13,TMX1,TM9SF2,TM9SF3,TNPO1,TNPO3,TOE1,TOMM22,TOMM40,TOMM70,TOP1,TOR1AIP1,TOR1AIP2,TPI1,TPM1,TPM2,TPP2,TPR,TPT1,TP53,TP53BP1,TP53TG3,TRADD,TRAF2,TRAPPC3,TRAP1,TRIB2,TRIM27,TRIP13,TRMT1,TRMT10C,TRMT112,TSFM,TSG101,TSN,TSNAX,TSPO,TSR1,TTC4,TTC27,TTI1,TTK,TTLL1,TTLL3,TTLL12,TUBAL3,TUBA1A,TUBA1B,TUBA1C,TUBA4A,TUBB,TUBB1,TUBB2A,TUBB2B,TUBB3,TUBB4A,TUBB4B,TUBB6,TUBGCP2,TUBG1,TUFM,TWF2,TXN,TXNDC5,TXNDC17,UAP1,UBAP2,UBAP2L,UBA1,UBA5,UBA6,UBA52,UBB,UBC,UBE2D3,UBE2E1,UBE2I,UBE2K,UBE2L3,UBE2M,UBE2N,UBE2O,UBE2Q1,UBE2S,UBE2T,UBE2V1,UBE2Z,UBE3C,UBE4A,UBR4,UBXN10,UCHL1,UCHL5,UCK2,UFD1,UFL1,UGDH,UGGT1,UHRF1,UMPS,UNC45A,UPF1,UQCC1,UQCRB,UQCRC1,UQCRC2,UQCRFS1,UQCRQ,UQCR10,USO1,USP7,USP9X,USP10,USP15,USP24,USP39,U2AF1,U2AF2,U2SURP,VARS1,VASH2,VCL,VDAC1,VDAC2,VDAC3,VGF,VGLL4,VIM,VMP1,VN1R1,VPS4A,VPS26A,VPS35,VPS45,VTA1,WARS1,WASF1,WASF2,WDR5,WDR6,WDR12,WDR18,WDR26,WDR41,WDR77,WDR91,WIPF1,WRNIP1,WSB1,WT1,XIRP2,XPNPEP1,XPO7,XP32,XRCC5,XRCC6,YARS1,YARS2,YBX1,YKT6,YME1L1,YTHDC2,YTHDF2,YTHDF3,YWHAB,YWHAE,YWHAH,YWHAQ,YWHAZ,YY1,ZAP70,ZC3HAV1L,ZC3H11A,ZC3H15,ZFAND5,ZMPSTE24,ZMYM5,ZNF598,ZNF622,ZNF638,ZRANB2,ZSCAN26,ZZZ3*
***MDK***	Neurite outgrowth-promoting factor 2 (P21741)	Pleiotrophin family	*LRP6*	*APP,SHANK3,NOS1AP*	*ACE,ACTG1,AFF4,AKT1,ARAF,BRAF,BOD1L1,BRD2,CCL3,CHD9,CLK2,CWC22,CXXC1,DCAF5,EGFR,EPB41L5,ESR2,FBXO7,FXR1,GPC2,HCK,HDGF,HIF1A,HRAS,H1-1,ITGA4,ITGA6,ITGB1,ITPKB,JOSD2,KBTBD7,KRAS,KSR1,LRP1,LRP8,MAPK1,MAPK3,MAPK6,MLLT3,MYOCD,NAF1,NCAM1,NCL,NFU1,NID1,NID2,NKAP,NOTCH2,PBX3,PDE4DIP,PNN,PPIG,PTPN9,PTPRA,PTPRZ1,RAF1,RBFA,REM1,RPL18A,RPL36AL,RPP25,RPS27A,SDC4,SETD1A,SIN3A,SORL1,SOST,SRPK2,SRRM2,TMED8,TNRC18,TUBA1A,TUBB2B,UBE2O,UBQLN1,UBQLN4,UBTF,ZCCHC17,ZHX2*
***MYOC***	Myocilin (Q99972)	–	–	–	*A2M,ACTA2,ACTB,ACTG1,ALDOA,ANXA2,ASB10,C1QB,CAP1,CDKN2B,CD81,CKM,CLIC1,COL1A2,COL3A1,CRYBB1,CYP1B1,ECE1,EEF1A1,ENO1,FBN1,FLRT3,FN1,FTL,FUBP1,GAPDH,HAGH,HSPA4,IGKC,IGLL1,ITGA7,LAMA5,LGALS3,LTBP2,MAEA,MYH11,MYL2,NOTCH2,OLFML1,OLFM3,OLFML3,OPTN,PEX5,PKM,PLPP6,PRRC2C,RFC1,SERPINF1,SGTA,SOST,SPARC,SPARCL1,SUCO,TGFBR1,TIMP1,TKT,TMCO1,TMTC1,TNFRSF1A,TPM1,WDR36,VAMP4,VIM*
***MYO1C***	Myosin heavy chain myr 2 (O00159)	TRAFAC class myosin-kinesin ATPase superfamily, Myosin family	*CALM1,CALM2,CALM3,CDH23,CTNNB1,ITPR1,LRRK2,MYO5A,SPHK1*	*CTNNB1,PTEN*	*AAK1,ABLIM1,ACTA1,ACTA2,ACTB,ACTC1,ACTG1,ACTN1,ACTN4,ACTR2,ACTR3,ADD1,AFAP1,AGGF1,AGR2,ANLN,AP2A1,AP2M1,AP3M1,ARHGAP11A,ARHGAP21,ARNT,ARPC1B,ARPC2,ARPC3,ARPC4,ARPC5L,ARRB1,ARRB2,BAP18,BASP1,BAZ1B,BCAR1,BCL2L1,BICD2,BMPR1A,BMP2K,CALML3,CAMK2G,CAPZA1,CAPZA2,CAPZB,CARD,CARD10,CBL,CCAR1,CCDC127,CDC25C,CDC73,CDKN2A,CDK2,CDK9,CDKN2A,CD2AP,CD109,CEP162,CFL1,CFL2,CFTR,CGAS,CHCHD3,CHERP,CIAO1,CLASP1,CLINT1,CLTA,CLTB,CLTC,CLTCL1,COBL,COPE,CORO1B,CORO1C,CORO2A,COX15,CRIP2,CSF1,CSTF2T,CTTN,DAB2,DAPK3,DBN1,DCTN1,DCTN2,DCTN3,DCTN5,DDX21,DDX39B,DEK,DENND1A,DENND2B,DIXDC1,DLST,DNAJA3,DOCK7,DST,DSTN,DUSP7,DUSP21,EED,EGFR,EGLN3,EIF3B,EIF3I,EIF4A2,EMD,EPS15,ERCC6,ESR1,ESR2,EXOC2,EXOC4,EXOC7,EXOC8,E2F3,FANCD2,FARP1,FBL,FBXO25,FBXO30,FBXO46,FCHO2,FLII,FLNA,FLNB,GAK,GAS2L1,GAS2L3,GIPC1,GOLGA2,GRB2,GRK5,GSN,GTF2F1,GTSE1,HDGFL2,HIP1R,HMGB2,HNRNPR,HSPA8,HSP90AA1,IKBKG,ILF3,ILKAP,IMPDH1,IMPDH2,INF2,IQCB1,IQGAP1,ITPRID2,ITPR2,ITPR3,JMY,JPH1,JUN,KAT2B,KAT6A,KCTD10,KDR,KHDRBS1,KIF11,KSR1,LARP4,LASP1,LIMA1,LIMCH1,LMNA,LMO7,LMX1B,LRCH3,LRRFIP2,LUZP1,LZTS2,L1TD1,MAP3K3,MCM5,MCM7,MECOM,MED12,MED15,MED16,MEX3C,MISP,MIS12,MPRIP,MRM1,MRPL55,MTUS1,MYBBP1A,MYC,MYH9,MYH10,MYLK,MYL6,MYL6B,MYL12A,MYO1B,MYO1E,MYO5B,MYO5C,MYO6,MYO9B,MYO18A,MYO19,NAP1L1,NCBP3,NCKIPSD,NEK6,NEXN,NPHP4,NSRP1,NTRK1,OCRL,OXR1,PALLD,PAN2,PCNP,PDGFRA,PDLIM7,PHLDA3,PHLDB2,PICALM,PIK3C2A,PLEKHB1,PLEKHG3,PLEKHG5,PNPO,POLR1B,POLR1E,PPFIBP1,PPP1CA,PPP1CB,PPP1CC,PPP1R3C,PPP1R9B,PPP1R12A,PPP1R12B,PPP1R15A,PPTC7,PRDM16,PRKAA1,PRKAB1,PSMA1,PTPN2,PTPRCAP,PTP4A3,PYCARD,RAB8A,RAB10,RAD18,RAI14,RALA,RBM5,RBM10,RBM17,RBM33,RIOK3,RIPK3,RPA1,RPA2,RPA3,RPS6KB2,RRAS2,RRN3,RRP1B,SAFB2,SAP18,SCAF8,SEC16A,SEPTIN7,SF3B1,SF3B2,SHC1,SIPA1,SIPA1L1,SIPA1L3,SIRT7,SLC2A4,SLX4,SMARCA5,SORBS2,SPECC1,SPECC1L,SPEF2,SPTAN1,SPTBN1,SPTBN2,SP1,SQOR,SRSF2,SSH1,SSH2,STON2,STRN3,STYXL1,SULT2B1,SUN2,SUZ12,SVIL,SYNPO,S100A10,TADA3,TES,TIMM8B,TIMM13,TIMM29,TJP1,TJP2,TLR9,TMED10,TMOD1,TMOD3,TMPO,TNF,TOM1,TPM1,TPM2,TPM4,TPRN,TP53,TRIM63,TRIOBP,TRIR,TRPS1,TUBA1A,TUBB3,TWF1,TWF2,UBE2Q2,UNC45A,USP20,UVRAG,U2SURP,VCL,VIRMA,WDFY4,WDR1,WRAP73,WWC1,YWHAE,YWHAZ,ZDHHC5,ZNF746,ZSCAN26*
***MYO1D***	Myosin heavy chain myr 4 (O94832)	TRAFAC class myosin-kinesin ATPase superfamily, Myosin family	*SPHK1,LRRK2*	*ATP6V1A,PTEN*	*ACTC1,ACTRT2,ASRGL1,ATG16L1,ATP6V1B1,BBS7,BTD,CANX,CCDC8,CDH5,CDK9,CFTR,CHUK,CIAO1,COX4I1,COX15,CUL1,DAPK1,DKK3,DLST,DNAI2,ECT2,EED,EGFR,EID1,ESR2,FANCD2,FBXO25,FBXO40,GOLGA2,HAVCR2,IKBKG,ITM2B,ILK,INVS,KIAA1429,KIAA2013,LSP1,L1TD1,MAP3K1,MAP3K3,MDC1,MGRN1,MYC,MYO1H,NEXN,OTUD4,PAN2,PARP2,PARP11,PAXIP1,PDHA1,PDXDC1,PELO,PEX19,PHB,PHLDA3,PLEKHA7,PPP1CA,PPP4C,PPP6R2,PRKAA1,PSMA1,PSMC4,RAB11FIP4,RALA,RCC2,RECQL4,RIPK2,RIPK3,SCN3B,SHC1,SIRT7,SLC2A4,SNAI1,SSH1,SSH2,SYNE1,TBC1D9B,TMPO,TNF,TOR1AIP2,TRMT61B,TSC22D1,TUBA1A,UBASH3B,UBB,UBC,UNC119,VCP,VIRMA,VSIG4,WDFY4,YWHAB,YWHAE,ZNF746,ZNRD2*
***MYO5A***	Dilute myosin heavy chain, non-muscle (Q9Y4I1)	TRAFAC class myosin-kinesin ATPase superfamily, Myosin family	*CALM1,CALM2,CALM3,LRRK2,MYO1C*	*CYFIP1,DISC1,GFAP,GRIN1,GRIN2B,SHANK2,SHANK3,SYNGAP1*	*ACE,ACTA1,ACTB,ACTR2,AGAP2,AKAP9,ANLN,AVIL,BCL2L1,BMF,CALML3,CAPZA2,CCDC8,CCNG2,CDC73,CDH5,CDK2,CDK9,CGAS,CORO1C,CREB3,CRYL1,CSF1,CYFIP2,DAO,DBN1,DCP1A,DCP1B,DKK3,DLC1,DLGAP1,DTNBP1,DYNLL1,DYNLL2,EID1,ELF5,ESR1,ESR2,EXOC3,EXOC4,EXOC6,EXOC7,FAM9B,FAM160B2,FANCD2,FBXO25,FBXW7,FEZ1,FLNA,FLNB,FLOT1,GPR156,GRIA1,GRIA2,HGS,HMGA2,HNRNPL,IL20RA,IQGAP1,ITGA5,ITM2B,KALRN,KBTBD7,LIMA1,LRRIQ1,MAPK3,MLPH,MYC,MYH9,MYH10,MYH11,MYL2,MYL12B,MYO1E,MYO5C,MYO18A,MYO19,MYRIP,NDEL1,NEFL,NEK2,NEURL4,NOS1,NPHP4,NTRK1,OS9,PAN2,PBXIP1,PDLIM7,PEX5L,PEX19,PLS3,PPP1CB,PPP1R9B,PPP6R2,PRKAA1,PRPH,RAB8A,RAB10,RAB11A,RAB27A,SCP2,SIRT7,SLC17A9,SMAD2,SNCA,SPIRE2,SSH1,SYNPO,TMOD1,TMOD3,TMOD4,TNF,TNIK,TNNC1,TPM1,TRAF3IP1,TRIM2,TRIM3,TRIM25,UBC,UBE2O,USP14,VIRMA,WDFY4,WRAP73,YWHAB,YWHAE,YWHAZ*
***MYO10***	Unconventional myosin-X (Q9HD67)	TRAFAC class myosin-kinesin ATPase superfamily, Myosin family	*CALM1,CALM2,CALM3*	*XPO1*	*ACTA1,ACTB,ACTG1,ACTR2,ACTR3,ARPC1A,ARPC1B,ARPC2,ARPC3,ARPC4,ARPC5,BTRC,CALML3,CAND1,CCDC77,CDK5RAP2,CEBPB,DCC,DYNLL1,ECPAS,ENAH,EVPL,FBXW11,FSCN1,HSP90B1,ITGB3,ITGB5,JPH4,MYO19,NEO1,NTN1,PAN2,PARD6B,PLA2G10,PRKCI,PRKD2,TAPT1,TERF2,TES,TRIM25,TUBA1A,TUBB2B,VASP,VIRMA,WIPI2*
***NCS1***	Neuronal calcium sensor 1, NCS-1 (P62166)	Recoverin family	*CADPS,ITPR1*	*DRD2,DRD3,GFAP,IL1RAPL1,KCND2,XPO1*	*ABHD6,ACO2,ARF1,ATP5F1A,ATP5F1B,BSPRY,CD69,CEP89,CNOT3,CTAG1A,C1QTNF2,DTX2,ELAVL1,FCGR3A,FSD1,FZD7,GRK1,GRK2,HNRNPL,HSD17B3,HSPA8,ICAM1,KCNC1,KCND1,KCND3,KDM3B,KIF21A,LINC00311,MEOX2,MIEF2,NAF1,NBL1,NMT1,NMT2,NUFIP1,OXER1,PDE1A,PI4KB,RBCK1,PRDM4,RNASEH1,RNF19B,SHARPIN,SIGLEC9,SLC1A6,SLC25A41,SNRPB,SPP1,SPRED2,SRP68,SSX3,TANK,TK1,TMEM185A,TNIP2,TRAF2,USP24,YWHAE,YWHAH,ZC3H10*
***NEUROD2***	Neurogenic differentiation factor 2 (Q15784)	–	–	*CPLX2,FMR1,MYT1L,TBR1,TCF4*	*ACTL6B,ANAPC1,ASCL1,CDC20,CKS1B,MT-ND2,KRT8,PGAP3,POU3F2,PKN1,RNF123,TK1*
***NLGN1***	Neuroligin-1 (Q8N2Q7)	Type-B carboxylesterase/lipase family	*DLG4,NRXN1,PICK1*	*DLG4,NLGN3,NRXN1,SHANK2,SHANK3*	*ABI2,ADCY9,AGAP2,BRK1,B3GAT3,CASK,CD247,DLGAP1,DLG2,DLG3,EDDM3A,GOPC,GRM5,MAGI1,MAGI2,MAGI3,NRXN2,NRXN3,PDZRN3,PTPRT,SHANK1,SIPA1L1,TAFA5,TRADD,TNFSF18*
***NPTX2***	Neuronal pentraxin-2 (P47972)	–	–	–	*AGR3,ARL10,AVP,BATF3,BDNF,CHURC1,CLEC4F,CYB5D2,EDARADD,GRIA1,GRIA4,LYPD4,MT-ATP6,MT-ATP8,MT-ND4,MYC,NPAS4,NPTX1,NPTXR,OCA2,RCN2,TOM1L1*
***NRXN1***	Neurexin-1 (Q9ULB1,P58400)	Neurexin family	*DLG4,LIN7C,NLGN1,SYT1*	*CNTNAP2,DLG4,LRRTM1,MYO16,NLGN2,NLGN3,NLGN4X,SHANK2,SHANK3*	*AFDN,APBA1,APBA2,ARHGAP10,ARHGAP26,ATP13A2,CASK,CBLN1,DLGAP1,DLGAP2,ELOA,GABRA1,GRID2,LNX2,LRRTM2,LRRTM3,MACF1,MIB1,NRXN2,NRXN3,PDZD2,PPFIA1,PRKDC,PTPRT,SHANK1,SIPA1L1,SYT13,SYTL1,SYTL2,SYTL3,TULP1*
***NSMF***	NMDA receptor synaptonuclear signaling and neuronal migration factor (Q6X4W1)	NSMF family	–	*TRO*	*ANOS1,CABP1,CCDC125,CCNDBP1,CHD7,EMILIN1,GFI1B,GNRHR,GNRH1,GOLGA2,GOLGA6L9,HSF2BP,HSPB3,HS6ST1,INA,ITM2B,KCTD15,KIFC3,KISS1R,KPNA1,LMO2,LXN,MAPK3,MID2,MPZ,MTNR1A,MTNR1B,MYL12B,NDUFAF5,POLR2A,PROKR2,PROK2,RBPJ,RNF123,PNMA1,PTPN21,SOCS7,SUPT5H,TACR3,TAC1,TAC3,TFIP11,TIMM9,TRIP6,UBASH3A*
***PAFAH1B1***	Platelet-activating factor acetylhydrolase IB subunit alpha (P43034)	WD repeat LIS1/nudF family	*CAPN2,LRRK2*	*DISC1,YWHAG*	*ACTR10,AHI1,ANAPC13,AP1G1,ARHGAP1,ARID4B,ARL2,ARMC8,ASNS,ATIC,ATXN3,BET1,BICD2,CALD1,CAPNS1,CAPZA2,CAPZB,CCSER2,CDC42,CDK2,CENPF,CENPK,CENPM,CEP128,CEP135,CEP152,CEP295,CHID1,CHMP2B,CLIP1,CSNK2A1,CSTF1,DCDC1,DCTN1,DCTN2,DCX,DIXDC1,DYNC1H1,DYNC1I1,DYNC1I2,DYNC1LI1,DYNC1LI2,DYNLL1,DYNLRB1,DYNLRB2,DYNLT1,DYNLT3,EDIL3,EIF5,FBXO7,FH,FSCN1,GAK,GID8,GNL3L,HECTD4,HMGB3,HSPA5,HSPA8,HSP90AA1,HSP90AA4P,HSP90AB1,HS6ST2,IMPACT,IPO4,IQCB1,IQGAP1,ISG15,ISOC1,KARS1,KATNA1,KATNB1,KCTD10,KIF11,KIF20B,LNX1,MAPRE1,MAPRE2,MAP1B,MPHOSPH6,MSN,MYC,MYO1E,MZT1,NDEL1,NDE1,NELFCD,NEMF,NTRK1,NUDCD2,NUDCD3,NUDC,PAFAH1B2,PAFAH1B3,PAK2,PAPSS1,PDE4B,PDE4D,PDIA4,PLS3,PPP1CA,PPP1R18,PPP2R1A,PRSS21,PSMA5,PSMD12,RNF4,RPL28,RPL36A,SBDS,SCLY,SDHC,SGO1,SKA3,SLC2A4,SLC25A12,SNRPD2,SNRPF,SNRPG,STUB1,TANK,TATDN1,TCOF1,TCTEX1D2,TMOD3,TNIK,TPM2,TRIM25,TUBA1A,UBA2,UBC,UBXN1,VPS72,WDR5,YWHAE,YWHAH,ZNF574,ZNF616*
***PAK1***	Protein p21-activated kinase 1 (Q13153)	Protein kinase superfamily, STE Ser/Thr protein kinase family, STE20 subfamily	*CIB1,RAC3*	*APP,CYFIP1,ERBB2,HDAC4,YWHAG*	*ABI3,ACVR2B,AKT1,ARHGDIA,ARHGEF2,ARHGEF6,ARHGEF7,ARPC1B,ATG5,BAD,BAIAP2,BMX,BRSK1,CAV1,CDC42,CDK5,CDK11A,CDK11B,CD47,CHORDC1,CPLANE1,CRIPAK,CTTN,DYNLL1,DYNLL2,DYRK1B,EGFR,EIF3E,ELF3,ESR1,FBXO28,FGB,FLNA,FSD1,FXR1,GIT1,GIT2,GNB1,GRB2,HACE1,HCN1,HGS,HNRNPAB,HRAS,HSP90AA1,H2AFV,H2AX,H3C1,H4C1,ICAM1,IKBKE,ILKAP,LIMK1,LPXN,LYN,MAP3K1,MAPK4,MAPK14,MYH10,MYLK,MYNN,MYO6,NCK1,NCK2,NF2,OR2K2,OXSR1,PAK1IP1,PAK2,PARP3,PCBP1,PGM1,PIK3CB,PKLR,PLCG1,PLK1,PPAT,PPM1A,PPP1CA,PPP2CA,PRKCZ,PXN,PYCARD,RACGAP1,RAC1,RAC2,RAF1,RELA,RHOA,RHOJ,RHOQ,RHOU,RPL7A,RPL27A,RPS6KB1,SCRIB,SETX,SH3KBP1,SLC9A3R2,SLC25A41,SNW1,SOX2,SRGAP2,SRSF2,SRSF5,TAS2R41,TAX1BP3,TBCB,TGFBR1,TGFBR2,TGM2,TNFRSF10B,TNFSF10,VIM,WDR26,YWHAE,YWHAZ,ZBTB18,ZC3H7A,ZNF83,ZNF418,ZNF768,ZNF823*
***PCDHA4***	Cadherin-related neuronal receptor 4 (Q9UN74)	–	*PCDHA7*	–	*APLP1,CCDC90B,CISD2,CLDN7,CLDN18,CLSTN1,CMA1,CNNM1,COG5,C1orf43,DDX11,EEF1A1,EIF2AK3,ERG28,FLT3,FYN,GAPDH,GDF9,HGS,IGSF21,IL25,KIAA0922,LONP2,LRIF1,LTBP1,MAP3K6,MAP7,NBEAL1,NMU,PCDHA1,PCDHA2,PCDHA6,PCDHA8,PCDHA9,PCDHA10,PCDHA11,PCDHA12,PCDHGA1,PCDHGA3,PCDHGB2,PCDHGB4,PCDHGC3,POMT1,RBM48,RBSN,RIF1,SETDB1,SGTA,SLC10A7,TEX13B,TLE1,TMEM39A,TMEM131,TMEM223,TP53,UFSP2,UBQLN1,UNC119,ZFP36L2,ZNF142*
***PCDHA7***	Cadherin-related neuronal receptor 7 (Q9UN72)	–	*CALM2,PCDHA4*	*APP*	*ABL1,ADRA1A,AQP11,CALM2,CLDN18,CMA1,CRK,FLT3,KCNJ5,PCDHA8,PCDHA9,PCDHA10,PCDHGA1,PCDHGA3,PCDHGB2,PCDHGB4,PNOC,RNF220,SLC6A15,TCHHL1,TEX13B,UHRF1BP1L,UQCRB,VPS13C,ZGPAT,ZSCAN23*
***PCDHB6***	Protocadherin beta-6 (Q9Y5E3)	–	–	–	*BPIFB3,CNEP1R1,CNTNAP5,KLHL5,LYPD6B,PCDHB16,SNN,TEX261,TMEM14B,TTC16,ZNF3,ZNF131,ZNF174,ZNF263*
***PCDHB8***	Protocadherin beta-8 (Q9UN66)	–	–	–	*CFAP54,CFTR,CNTNAP5,FBXL2,KIAA1671,LRRIQ4,OLFML1,PNCK,RNF180,SHC4,SLC25A2,STK32C,TAS2R60,TRIM45,VWA3B,ZNF605,ZNF706*
***PCDHGA4***	Protocadherin gamma-A4 (Q9Y5G9)	–	*-*	–	*CHD6,COL13A1,CSRP1,HOXD9,KIAA0513,KNDC1,LRRC71,NKPD1,OR52R1,PCYT2,PTPRK,PLCH2,PPP1R18,RHOU,SGTA,SLC25A2,VPS13C,XKR4,ZNF304*
***PCDH8***	Activity-regulated cadherin-like protein (O95206)	–	*NPTX2*	*DIPK2A*	*BRK1,BTRC,GSX1,HIVEP1,IREB2,KLHL1,MAPK14,MESP2,NAA16,PAPOLG,RNF112,STX2,TDRD3,TNIK,TROAP,UBQLN4*
***PCDH15***	Protocadherin-15 (Q96QU1)	–	*CDH23,DLG4*	*DLG4*	*ADGRV1,CFTR,LHFPL5,MYO7A,MYO15A,NANOG,POU5F1,TMC1,TMIE,USH1C,USH1G,USH2A,WHRN*
***PCLO***	Multidomain presynaptic cytomatrix protein (Q9Y6V0)	–	*DLG4,SNAP25,UNC13A*	*DAO,DLG4*	*AGAP2,AGR2,BSN,CEP72,DPPA4,ERC1,ERC2,ESR2,EWSR1,E2F3,FLOT1,GIT1,GIT2,GRIA1,HMGA1,HMGN2,H1-1,ITM2B,PEAR1,PFN1,PFN2,RABAC1,RAB3A,RAPGEF4,RARS2,RBCK1,RIMS2,RNF123,SEC22B,SEMA3E,SIAH1,SPACA6,TNIK,TSEN54,UNC13B,VIRMA,YWHAB,YWHAE*
***PDYN***	Proenkephalin-B (P01213)	Dynorphin channel-forming neuropeptide (dynorphin) family	–	*APP,DRD2,DRD3,POMC*	*ACAD11,GAL,GNPTAB,GNPTG,LCN1,NPY,OPRD1,OPRL1,OPRM1,OPRK1,PENK,PNOC,PPARG,SST,S100A10,TIMM8A,TIMM8B,TIMM13*
***PICK1***	Protein kinase C-alpha-binding protein (Q9NRD5)	–	*GRM7,NLGN1,PRKCA,PRKCG*	*ERBB2,FMR1,GRIK1,GRM2,GRM7,HDAC4,NLGN2*	*ABT1,AEBP2,AGO2,AKAP9,AKT1,AKT2,ALKBH8,AMIGO1,APTX,AP1M1,AP1S1,ARFIP1,ARHGEF3,ARHGEF5,ARL6IP1,ARMCX1,ARPC2,ASIC1,ASIC2,ATP5IF1,ATP6AP1,ATP6AP2,ATP11A,ATXN1L,ATXN3,ATXN7L3,ATXN7,AVPI1,BAHD1,BCL2L14,BEX1,BIN3,BLOC1S2,BLK,BNC1,BOLA3,BRD1,BTG2,BUD31,BYSL,CARD9,CBX8,CCDC36,CCDC102B,CCDC103,CCDC187,CCNH,CDCA7L,CDC42EP2,CDC73,CDKL3,CDKN2B,CDKN2D,CDK2AP1,CEP19,CEP57L1,CEP89,CEP95,CEP290,CGGBP1,CHMP1B,CIC,COIL,CPNE2,CPNE7,CSNK2A2,CTSG,CUTC,CWF19L2,CYP21A2,C2CD5,C4orf46,C8orf33,DCTD,DCUN1D5,DDX6,DDX55,DMC1,DMD,DNAJB13,DNTTIP1,DNTTIP2,DPEP1,DPF2,DSE,DRAP1,DTNB,DUPD1,EAF1,EEF2KMT,EFHC2,EFNB1,EFNB2,EHD2,EHHADH,EIF1AD,EIF3CL,EIF3D,EIF4A3,EIF4EBP1,EIF4H,EIF5A,ENKD1,EPHA7,EPHB2,EPM2AIP1,ERBIN,ESCO2,EXOSC5,FAM9A,FAM90A1,FAM117A,FAM161B,FAM207A,FAM214B,FAM219B,FBXL3,FBXL8,FGF16,FHOD1,FKBP6,FLJ34048,FLYWCH1,FXN,F11R,GADD45GIP1,GAS2L2,GFI1,GFI1B,GLYCTK,GLYR1,GMCL2,GNPTAB,GPATCH2,GPATCH11,GPC4,GPKOW,GPR37,GRB7,GRB10,GRIA1,GRIA2,GRIA3,GRIA4,GRIK2,GRIP1,GRIP2,GRM3,GRM7,GRXCR1,GTF2E2,GTPBP2,HEXIM2,HMBOX1,HMBS,HMG20A,HNRNPC,HOPX,HOXA5,HSD17B14,HSF2,HSF2BP,ICA1,ICA1L,ID2,ILF2,ILVBL,IL16,INO80B,INO80E,INPP5J,IP6K1,ISCU,ITGA6,JAM2,JAM3,JRK,KAT5,KCTD1,KCTD6,KCTD9,KIAA1328,KLHL11,KLHL24,LCLAT1,LCN2,LDB1,LGALS14,LMO1,LMO3,LONRF1,LRRC73,LZTFL1,LZTS1,L3MBTL2,MAGEA1,MAGEA4,MAGEB4,MAPK9,MAPRE3,MAP2K6,MAP3K4,MAZ,MBD3,MCM10,MEOX2,MGME1,MID2,MMTAG2,MNS1,MOB3C,MORF4L1,MORF4L2,MORN3,MOS,MRI1,MRNIP,MSRB3,MSS51,MTA1,MTG1,NATD1,NDEL1,NDRG3,NECAB2,NECTIN1,NECTIN2,NECTIN3,NECTIN4,NEFL,NEK6,NFS1,NME7,NMNAT1,NOC4L,NSF,OARD1,OPTN,OSBP2,OSGIN1,PAFAH1B3,PAX6,PBX4,PCBD1,PDCD5,PDS5A,PEBP1,PHF19,PIBF1,PKD2,PKN1,PLEKHA7,PNKP,PNO1,POLL,PPL,PRKCB,PRKN,PRLHR,PRPF18,PRPF31,PRPF40A,PRXL2C,PSMA1,PSME3,PTRH1,QARS,QSOX2,RAD51D,RASAL3,RASSF7,REL,REXO1L1P,RFC3,RIMS3,RIN1,RNF8,RNF181,RNPS1,ROBO3,ROPN1,RPIA,RPP25,RRP8,RXRB,RXRG,SACS,SCAND1,SCNM1,SEMA3B,SERBP1,SERTAD1,SERTAD3,SHFL,SH2D4A,SH3GLB2,SLC6A2,SLC6A3,SLC6A4,SLIRP,SMARCB1,SMARCD1,SNRNP25,SNRNP70,SNRPA1,SNRPB2,SNW1,SPANXN2,SPATC1L,SPEG,SPTBN1,SSNA1,STK4,STK19,SYT17,TANK,TBC1D7,TBC1D22B,TCEANC,TCEANC2,TCEA2,TCP10L,TDO2,TEX101,TFIP11,THAP6,THAP7,TLE5,TLNRD1,TMF1,TPM4,TRAF3IP1,TRAF4,TRAF5,TRMT2A,TRIM44,TRIML2,TRIM54,TSGA10IP,TSN,TSPAN7,TSTD2,TTC23,TTC23L,TXNDC9,TXNL4B,TYW3,UACA,UBE2E3,UBE2K,UBQLN4,UGCG,USHBP1,USP2,USP7,UTP3,VAX1,VEZF1,VPS25,WT1,XPA,YES1,YPEL2,YTHDC1,ZBED1,ZBTB2,ZBTB24,ZBTB49,ZFHX3,ZFP2,ZFP91,ZMAT2,ZMYND12,ZNF17,ZNF24,ZNF35,ZNF71,ZNF165,ZNF205,ZNF250,ZNF264,ZNF276,ZNF286A,ZNF329,ZNF330,ZNF408,ZNF410,ZNF414,ZNF417,ZNF438,ZNF497,ZNF524,ZNF575,ZNF576,ZNF593,ZNF624,ZNF691,ZNF764,ZNF774,ZSCAN21,ZSCAN23,ZZZ3*
***PINK1***	Serine/threonine-protein kinase PINK1, mitochondrial (Q9BXM7)	Protein kinase superfamily, Ser/Thr protein kinase family	*LRRK2,PRKN*	*MAP1B,PTEN*	*AKT1,APPL2,BAG2,BAG5,BCL2L1,BCL2L2,BECN1,BNIP3,CDC37,CLU,COPS8,CRLS1,DLD,DNM1L,ERLIN1,FBXO7,HSD17B10,HSH2D,HSP90AA1,HSP90AB1,HSPA1A,HSPA1B,HSPA4,HTRA2,IMMT,IRAK1,IRS4,KIF11,KRAS,MAP1LC3A,MAP1LC3B,MAP3K7,MAPKAP1,MARK2,MCL1,MFN1,MFN2,NAE1,NFKBIE,PARK7,PARL,PGAM5,PRKDC,PRKN,PSMC6,PSMD1,PSMD2,RB1CC1,RHOT1,RHOT2,RICTOR,SAMM50,SARM1,SNCAIP,SNCA,SOCS4,SQSTM1,STAT3,TGM2,TIMM23,TOLLIP,TOMM20,TOMM22,TOMM40,TOMM70A,TRAF6,TRAK1,TRAP1,TUBA1A,TUBB,UBC,UBE2M,VDAC1,VIM,YWHAZ*
***PPP5C***	Serine/threonine-protein phosphatase 5 (P53041)	PPP phosphatase family, PP-T subfamily	*CPNE1*	*APP,DISC1,GRIN1,GRIN2B,HTR2A,HUWE1,SHMT2,XPO1*	*ABCF2,ABHD14A,ACTB,ACTG1,AGO1,AGO3,AHSA1,AHSG,BAG2,BPHL,BZW2,CAD,CCT4,CDC16,CDC27,CDC37,CDC37L1,CDC42BPB,CDK9,CDK18,CDK20,CHORDC1,CLU,CNTLN,CPNE2,CPNE4,CRY1,CRY2,CSE1L,CSNK1D,CSNK1E,CTPS1,DCPS,DDB1,DFFA,DIS3,DUSP8,DUT,EGFR,EGLN3,EIF6,ELP1,ELP3,ENO1,ESR1,ESR2,FKBP4,FKBP5,FLCN,FLII,FSCN3,FUBP1,GADL1,GAPDH,GINS3,GNA12,GNA13,GNAI1,GRB2,GSS,HACD3,HNRNPA1,HRAS,HSPA1A,HSPA1B,HSPA4,HSPA6,HSPA8,HSP90AA1,HSP90AA5P,HSP90AB1,HSP90AB4P,IRS4,ITGB1BP2,KSR2,LRRC1,MAPK1,MAPK3,MAPT,MAP3K5,MAP3K6,MAP3K7,MINPP1,MOV10,NAPRT,NOL9,NOL10,NR3C1,NR3C2,NTRK1,NUDC,NXF1,PAFAH1B2,PAPSS1,PGR,PHLPP1,PLD3,POLD2,PPIB,PPID,PPP2CA,PPP2R1A,PPP2R3A,PRPF8,PSEN2,PSPC1,PTGES3,RAB41,RACK1,RAP1GDS1,RBBP5,RBBP6,RECQL4,RIPK4,RPS6KB1,SNRNP200,STIP1,SUGT1,TATDN1,TNIP2,TP53BP1,TPTE,TRIM25,TUFM,ULK2,USP45,USP49,VCL,VHL,VIM,WIPI2,WWOX,YAF2,YWHAB,YWHAE,YWHAQ*
***PRKCA***	Protein kinase C alpha type (P17252)	Protein kinase superfamily, AGC Ser/Thr protein kinase family, PKC subfamily	*CACYBP,CALM1,CTNNB1,DLG4,GRM7,PICK1,PRKCG*	*APC,AVPR1A,CTNNB1,DLG4,GABRB3,GRIN1,GRIN2B,GRM7,MTOR,SHANK3,SLC1A1,TRPC6,YWHAG*	*ACIN1,AFAP1,AGAP2,AKAP5,AKAP12,AKAP13,ANXA6,ARFGEF3,ATM,ATP1A1,AVPR1B,BAP1,BCAS2,BRAF,BTG2,CASR,CAV1,CBL,CCDC8,CCNL2,CD9,CD53,CD151,CDKN2A,CHRM1,CHUK,CISH,CLOCK,CSNK1D,CTNNBL1,CYP2S1,CYP3A4,C1QBP,DDX58,DLG3,DSP,DUSP11,EGFR,EGLN2,EIF2S1,EIF4EBP1,ELAVL1,EP300,EZR,FANCD2,FAS,FBXO7,FBXO21,FBXO25,FBXW7,FCGR2B,FCGR3A,FGD4,FKBP1A,FN3K,FSCN1,GCLM,GIPC1,GJA1,GLI3,GNRHR,GRB2,GRIA1,GRIA2,GRIA4,GRM5,GSK3A,GSK3B,HABP4,HAND1,HAND2,HDAC6,HIST1H1T,HIST3H3,HMGA1,HMGA2,HMGN1,HMGN2,HOMER1,HR,HSP90AA1,HSP90AB1,H1-1,H1-3,H1-4,H1-5,H3C1,IBTK,IKBKB,ITGB1,ITGB2,ITGB3,JAK1,JAK2,JSRP1,KDR,KIF2A,KIF11,KLF5,KSR1,LDB3,LIMK1,LMNA,LMNB1,LNX1,LNX2,MAB21L2,MAPK1,MAPK3,MAPK7,MAPKAP1,MARCKS,MBP,MGMT,MOV10,MST1R,MYC,NCF1,NF2,NFE2L2,NFKBIA,NFKB1,NOXA1,NPM1,NR1H2,NTRK1,NUMB,NUMB,NXF1,OGG1,OR2T10,PATJ,PDLIM7,PFKFB1,PHB2,PIK3CA,PIM1,PIP5K1A,PLA2G4A,PLCB1,PLCG1,PLCG2,PLD1,PLD2,PPARG,PPM1A,PPP1R14A,PPP3CA,PRKCB,PRKCD,PRKCE,PRKCH,PSMB4,PTGIR,PTK2,PXN,RACK1,RAC1,RALBP1,RBCK1,RGS2,RICTOR,RNF31,RPS6KA2,RRAD,SACM1L,SCRIB,SDC2,SDC4,SELL,SHCBP1,SLC9A3R1,SLC9A3R2,SLC25A11,SLC25A41,SMURF1,SNX16,SPAG1,SRC,STARD7,STXBP1,S1PR1,S1PR2,S1PR3,S1PR5,TAS2R7,TBXA2R,TES,TIAM1,TMEM185A,TNP1,TNP2,TOP2A,TRIM41,TRPC6,TRPV6,TUBA1A,TUBA1B,TYK2,UBC,UQCRB,VCL,VIRMA,VPS4B,VTN,WWC1,WWC2,WWC3,YWHAE,YWHAZ*
***PRKCG***	Protein kinase C gamma type (P05129)	Protein kinase superfamily, AGC Ser/Thr protein kinase family, PKC subfamily	*DLG4,HMGB1,PICK1,PRKCA*	*CYFIP1,DAO,DLG4,FMR1,GABRB1,GRIN1,GRIN2B,HUWE1,SHANK3,SYNGAP1*	*,ADRB2,AGAP2,ANXA7,BCL2,CCHCR1,CD5,CLOCK,CNKSR2,DACT1,DACT2,DACT3,DLGAP1,DMXL2,DVL2,EXOC5,GABRG2,GABRR1,GJA1,GLRB,GNG3,GNG13,GRIA1,GRIA2,GRIA3,GRIA4,GRK2,HSP90AB1,H1-1,H1-2,ITM2B,LDB3,LIMK1,LNX1,MAPT,MTNR1B,NOXA1,PDE4DIP,PEBP1,PLCG1,PLCG2,PPP1R9B,PTPN1,SNCA,STXBP1,STXBP3,TNIK,TRAF6,TRPC3,TSC1,UBC,YWHAB,YWHAE,YWHAZ*
***PRKN***	E3 ubiquitin-protein ligase parkin (O60260)	RBR family, Parkin subfamily	*APEX1, CACNA1B,CALM1,CAMK2A,CTNNB1,DLG4,LRRK2,PICK1,PINK1,PRKCA,SYN1,SYT4,TPM3*	*APP,CTNNB1,DLG4,DYRK1A,GRIN1,GRIN2B,HDAC4,RPTOR,TCF4*	*ABCE1,ABL1,ACSL1,ACSL4,ACTA1,ACTA2,ACTB,ACTBL2,ACTB,ACTC1,ADRM1,ADRM1,AFDN,AGK,AGPS,AHCY,AIFM1,AIMP2,ALB,ALDOA,AMBRA1,AMOT,AMY2B,ANXA2,ANXA11,ARIH1,ARRB1,ARRB2,ATAD1,ATAD3A,ATAD3B,ATG7,ATP2B1,ATP2B3,ATP5F1A,ATP5F1C,ATP5MF,ATP5PO,ATXN2,ATXN3,AURKA,AURKB,BACH1,BAG2,BAG5,BAX,BBOX1,BCL2,BCL2L1,BCL2L2,BECN1,BIRC5,BNIP3,B9D1,CALCOCO2,CALM1P1,CALML3,CALR,CALU,CASK,CASP14,CAV1,CBX1,CBX3,CCNA1,CCNB1,CCND2,CCND3,CCNE1,CCT2,CCT3,CCT4,CCT5,CCT6A,CCT7,CCT8,CDC20,CDC34,CDK5,CEP55,CFL1,CHCHD3,CHGA,CHGB,CHPF,CIRBP,CISD1,CKAP4,CLPX,COMMD1,CPT1A,CRX,CRYAB,CSE1L,CSNK1D,CUL1,CYB5R1,CYB5R3,C1QBP,DAP3,DBT,DDIT3,DDIT4,DDX3X,DDX5,DDX17,DDX47,DLD,DLG1,DLX2,DNAJA1,DNAJB1,DNM1L,EEF1A1P5,EEF1A1,EEF1A2,EEF2,EGFR,EIF4B,EIF5A,ELAVL1,ENO1,EPS15,ESRRA,ESRRB,ESRRG,EZR,FAF1,FASN,FBP1,FBXO4,FBXO7,FBXW7,FIS1,FKBP4,FUBP1,FUS,FZR1,GAPDH,GBA,GCNT2,GPD2,GPR37,GRIK2,GRIK2,GSDMA,GSTP1,HADHB,HDAC6,HDGF,HDLBP,HERC5,HEXD,HIF1A,HIST1H2AA,HK1,HK2,HNRNPAB,HNRNPA1,HNRNPA2B1,HNRNPA3,HNRNPCL1,HNRNPC,HNRNPDL,HNRNPD,HNRNPF,HNRNPH1,HNRNPH3,HNRNPK,HNRNPL,HNRNPM,HNRNPR,HNRNPU,HOMER1,HSD17B10,HSDL2,HSPA1A,HSPA1L,HSPA2,HSPA4,HSPA5,HSPA7,HSPA8,HSPA9,HSPD1,HSP90AA1,HSP90AB1,HSP90B1,HTRA2,H1-0,H1-2,H4C11,IGF2BP1,IGHA1,IGHG1,IGKV2-30,IKBKG,IMPDH2,INA,LIMK1,LDHA,LDHB,LMNA,LMNB1,LRPPRC,MAPK1,MAPT,MAP1B,MAP1LC3B,MARCH5,MARCKSL1,MARCKS,MATR3,MAT2A,MAVS,MCCC2,MCL1,MCM7,MDH1,MDH2,MEOX1,MEOX2,MFN1,MFN2,MICU1,MRPL12,MRPS7,MRPS15,MRPS18B,MRPS22,MRPS23,MRPS27,MRPS28,MRPS31,MTARC1,MTOR,MYL12A,MYO6,NACA,NACC1,NBN,NCL,NDUFA4L2,NDUFA10,NDUFS2,NDUFS3,NDUFS4,NEFL,NEFM,NEK2,NFKBIE,NIPSNAP1,NME2,NOD2,NPM1,NTPCR,OPA1,OPTN,OSGIN1,PAAF1,PABPC1L,PABPC3,PACRG,PAFAH1B2,PARK7,PASK,PA2G4,PCBP1,PCCA,PCCB,PCNA,PCYT2,PDCD2,PDIA3,PDIA4,PDIA6,PFN1,PGAM1,PGAM5,PIK3C3,PIP,PKM,PLCG1,PLK1,PPIA,PRAME,PRDX1,PRDX2,PRKACA,PRKCSH,PRPF6,PRPF19,PRPS1,PRRC2C,PSMA1,PSMA2,PSMA3,PSMA4,PSMA5,PSMA6,PSMA7,PSMB1,PSMB2,PSMB3,PSMB4,PSMB5,PSMB6,PSMB7,PSMC1,PSMC2,PSMC3,PSMC4,PSMC5,PSMC6,PSMD1,PSMD2,PSMD3,PSMD4,PSMD5,PSMD6,PSMD7,PSMD8,PSMD9,PSMD10,PSMD11,PSMD12,PSMD13,PSMD14,PSME1,PSME2,PSME3,PTCD3,PTPN5,PTTG1,P4HB,RAB7A,RAC1,RAD1,RAD23A,RAD23B,RALY,RANBP2,RBCK1,RBM8A,RCN2,REL,RFC4,RGS2,RHOT1,RHOT2,RIPK1,RNF31,RNF41,RPLP0,RPLP2,RPL4,RPL6,RPL7A,RPL7,RPL8,RPL11,RPL12,RPL13,RPL22,RPL23A,RPL26L1,RPS2,RPS6,RPS8,RPS13,RPS14,RPS16,RPS19,RPS20,RPS25,RPS27A,RUVBL1,RUVBL2,SART1,SEPTIN5,SERBP1,SET,SFPQ,SH3GL1,SH3GL2,SH3GL3,SIM2,SLC6A3,SLC11A2,SLC25A1,SLC25A3,SLC25A4,SLC25A5,SLC25A13,SLC25A24,SNCA,SNCAIP,SNRNP200,SQSTM1,SRSF1,SRSF2,SRSF3,SRSF9,SSBP1,STOML2,STUB1,SUMO1,SYNCRIP,SYNJ1,SYT11,TARDBP,TAX1BP1,TBC1D15,TBK1,TCHP,TCP1,TENT5C,TFRC,TGM3,TKT,TNFRSF17,TOM1,TOMM22,TOMM40,TOMM70A,TPI1,TPM1,TPM4,TP53,TRAF2,TRAF3,TRAF6,TRIB3,TRIP13,TUBA1A,TUBA1C,TUBA3D,TUBA4A,TUBB,TUBB1,TUBB4B,TUBB4Q,TUBB6,TUBG1,TUFM,TXN,UBASH3A,UBASH3B,UBA52,UBB,UBC,UBE2A,UBE2B,UBE2C,UBE2D1,UBE2D2,UBE2D3,UBE2D4,UBE2E1,UBE2E2,UBE2E3,UBE2G1,UBE2G2,UBE2H,UBE2J1,UBE2J2,UBE2K,UBE2L3,UBE2L6,UBE2M,UBE2N,UBE2O,UBE2R2,UBE2S,UBE2T,UBE2V1,UBE2Z,UBL4A,UCHL1,USP8,USP30,VAT1,VCP,VDAC1,VDAC2,VDAC3,VGF,VIM,VPS35,XRCC5,XRCC6,YBX3,YWHAE,YWHAH,YWHAZ,ZC3H15,ZNF746*
***PRRT2***	Dispanin subfamily B member 3 (Q7Z6L0)	CD225/Dispanin family	*DLG4,SNAP25,SYT1*	*CNIH2,CNIH3,DLG4*	*ABHD6,ADRA1B,AQP6,ASPHD1,CACNG2,CACNG3,CACNG4,CACNG7,CACNG8,CALML5,CDIPT,CPT1C,C2CD2L,DDI1,DIRAS2,ELK1,FRRS1L,F2RL1,GPRC5D,GRIA1,GRIA2,GRIA3,GRIA4,GSG1L,HIRIP3,HRAS,INO80E,KIF22,KIT,KRAS,LCLAT1,LRRTM4,MAPK1,MAPK3,MFSD14B,NDUFB8,NDUFB9,NDUFS4,NDUFV3,NRN1,OLFM1,OLFM2,OLFM3,PNKD,PRKD1,PRRT1,SDC3,SEZ6L2,SHISA9,SLC10A6,SMIM5,TBRG4,TLCD4,VEGFC,VWC2,VWC2L,YPEL3*
***PTK2B***	Focal adhesion kinase 2 (Q14289)	Protein kinase superfamily, Tyr protein kinase family, FAK subfamily	*CAMK2A,DLG4*	*DLG4,ERBB2,GRIN1,GRIN2A,GRIN2B,PTEN,SYNGAP1*	*AGAP2,ALG2,ANXA6,ARFGAP1,ARHGAP10,ARHGAP35,ASAP1,ASAP2,BCAR1,CASS4,CBLB,CBL,CD47,CLTC,CRK,DLG3,DNM1,DOCK8,DOK1,EED,EFS,EGFR,EPHA1,ERBB3,ERBB4,EWSR1,EXOC6B,FANCD2,FGR,FYN,GIT1,GNA13,GP1BB,GP6,GRB2,GSN,HSP90AA1,HSP90AB1,IL7R,ITGAV,ITGB1,ITGB2,ITGB3,JAK1,JAK2,JAK3,KCNA2,KDR,LCK,LPXN,LYN,MAPT,MAP3K4,MATK,MBD2,MCAM,MYD88,NEDD4L,NEDD4,NEDD9,NPHP1,NPHP4,PAX6,PDCD6IP,PHLPP1,PIK3CB,PIK3R1,PITPNM1,PITPNM2,PITPNM3,PLD2,PPP2R2B,PRKCD,PRKCE,PTPN6,PTPN11,PTPN12,PTPRO,PXN,RASA1,RB1CC1,RHOA,RHOU,ROCK2,SHC1,SH3KBP1,SIRPA,SKAP2,SLC2A1,SOCS1,SOCS2,SOCS3,SORBS2,SOS1,SRC,SYK,TGFB1I1,TLN1,TNS2,TP53,TRAF4,VAV1,VEGFA,VIRMA,XPO7,YWHAE*
***RAC3***	Ras-related C3 botulinum toxin substrate 3 (P60763)	Small GTPase superfamily, Rho family	*CACYBP,CIB1,PAK1*	–	*ARFIP2,ARHGDIA,ARHGDIB,ARHGDIG,CALML3,CWC15,DDX46,DUSP9,ESR1,FBXL19,GHRH,GOLGA2,HDAC7,HIVEP2,HNRNPK,IQGAP3,ITSN1,KALRN,KHDRBS1,LDHB,LY86,NAP1L1,NCL,NEFM,NRBP1,PAK4,PPP1R9A,PREX1,PTBP3,RAC1,RAC2,RAP1GDS1,RBM41,RHOC,RSRC1,RWDD1,RXRA,SLC7A1,SLC9A6,TGFA,TRIO,VAV1,VAV2,VAV3,WAS,XIAP,YWHAE,ZNF706*
***RASGRF1***	Ras-specific nucleotide exchange factor CDC25 (Q13972)	–	*CAMK2A,DLG4*	*DLG4,GRIN1,GRIN2A,GRIN2B*	*CAMK2G,CDC42,CEBPA,CTNND1,EHD4,FKBP5,GRK2,HRAS,KRAS,MARK3,MDM2,MYC,NEFL,NGF,NRAS,NTRK1,NTRK2,NTRK3,PALM,PLK1,PPP1R9B,PTK2,RAC1,RASA1,RRAS,RHOA,SNRNP70,SRC,SRPK2,TP53,UBC,USP8,YWHAB*
***RORA***	Retinoid-related orphan receptor-alpha (P35398)	Nuclear hormone receptor family, NR1 subfamily	–	*GFAP*	*ARNTL,ATXN1,BHLHE41,CEBPB,CLOCK,COPS5,CRY1,CSNK1E,DCAF1,DDB1,EP300,EZH2,FOXP3,HIF1A,LMO3,LRIF1,MAGED1,MED1,MYOD1,NCOA1,NCOR1,NME2,NPAS2,NRIP1,NR0B1,NR1D1,NSD1,PIAS2,PIAS3,PIAS4,PNRC1,PNRC2,PPARGC1A,PROX1,PSD4,PSMC5,PTBP1,RORB,RORC,RUVBL1,SMARCD3,SP1,SREBF1,STAT3,SUMO1,UBE2I,YAP1,ZXDC*
***RPH3A***	Exophilin-1 (Q9Y2J0)	–	*CALM1,CALM2,CALM3,SNAP25,STX1A*	*PRNP*	*ADD2,ALOX15B,APPL1,CAND1,CASK,DOC2B,GCH1,HCN1,ITM2B,MPP5,NTRK1,PSEN1,RAB3A,RAB3B,RAB3C,RAB3D,RAB8A,RAB8B,RAB15,RAB27A,RAB27B,SNAP23,TP63,UBE3A,UNC13B,VAMP1,YWHAB,YWHAE,YWHAH*
***S100A1***	S100 calcium-binding protein A1 (P23297)	S-100 family	*CACYBP,S100A4*	*ATP2A2*	*AGER,ANXA6,BAG3,BEX3,BIK,CAPZA2,DES,FKBP4,FKBP5,FOPNL,GJA1,HSPA1,HSPA1A,HSPA1B,HSP90AA1,HSP90AB1,LY96,MDM2,NIF3L1,PGM1,PLB1,PLEKHF2,PPID,PYGM,REL,RYR1,SMAD2,S100A2,S100A3,S100B,S100P,S100Z,TLR4,TP53*
***S100A4***	Calvasculin (P26447)	S-100 family	*CDH1,S100A1*	–	*AGER,ANXA1,ANXA2,ANXA5,ANXA6,ANXA10,AREG,BAG6,BBX,BCKDHA,BCKDHB,BEX2,BRD1,BTC,CCDC14,CCDC28A,CCDC28B,CCN3,CDK2,CEP44,CEP76,CEP128,CEP170,CNTRL,CORO7,CRBN,DIAPH3,DNAJA3,DNAJB6,DPYSL4,DYNLL1,EGF,EGFR,ESCO2,ESS2,FAM24B,FCGR3A,FOPNL,GAK,GAN,GFER,GOLGA2,HAUS1,HAUS4,HBEGF,HSBP1,HSPB1,H2AX,IGBP1,IGHG3,KAT7,KIF18B,MATR3,MCM10,MDM2,METAP2,MMP9,MMP13,MOV10,MRPS34,MYH9,MYH14,MYL12A,NEDD1,NME7,NPAT,NPHP1,NR2C2,NXF1,OIP5,OSGEP,PATE2,PHF12,PKN3,POP4,POTEI,PPFIBP1,PSMA1,PTGR2,PXDN,RCBTB1,RCBTB2,RHOA,RHOU,RPRD2,SAMM50,SATB1,SATB2,SELENBP1,SLAIN1,SMAD2,SMAD3,SMC1A,SNRPF,SPATA5,SPC24,SPC25,SRPK2,SSNA1,STING1,STRN3,STXBP2,SUGT1,SUV39H1,SYNE2,S100A6,S100B,TCHHL1,TNF,TOM1L2,TP53,TRIP13,TTC21B,TXNIP,UBE3A,UMPS,UNC119,UNK,UQCR10,VCPIP1,VIM,VPS11,VPS39,VWA8,WDFY3,WDFY4,XIAP,XRCC4,YWHAE,ZBED1,ZFHX2,ZFHX4,ZZEF1*
***S100A9***	Migration inhibitory factor-related protein 14 (P06702)	S-100 family	–	*CPLX1,PTEN,TOP2B*	*AAR2,ACIN1,AIRE,ARRB1,ARRB2,ASB3,CAMP,CCNYL1,CDC34,CDC42,CDK15,CD2BP2,CD33,CD163,CFTR,CHD3,CHD4,COPS5,CSF3R,CUL2,CUL5,CYLD,DAB2,DDX19B,DDX21,DEK,DHX15,DSTN,EGFR,EIF4A3,EWSR1,FLT1,GABARAPL1,GABARAPL2,GAPDH,GNPAT,GPATCH2L,GRB2,GRK4,GTF3C1,G3BP1,HNRNPU,IFI16,ILK,IRAK1,KIAA1429,KPNA2,KRT17,LCN2,LTF,LUC7L3,LY96,MAGEA1,MAGEA3,MAGEA6,MAPK14,MAPT,MAP1LC3A,MATR3,MCM2,MCM5,METTL23,MRPL38,MYC,MYD88,NCF2,NCSTN,NF2,NIFK,NME2P1,NOS2,NPPA,NTRK1,NUAK1,NUBP2,PAK5,PCYOX1,PDE4DIP,PI4KA,PPP2R1A,PPP2R2A,PPP2R2B,PRDM16,PRPF8,PSMA5,PSMB7,PSRC1,RAD21,RBM14,RBM39,RNF4,RNPS1,SAP18,SART1,SF3B6,SHC1,SINHCAF,SIRT6,SMARCA5,SON,SPDL1,SRRT,SRSF3,SRSF5,SRSF7,SRSF10,SRSF11,SSRP1,SUPT16H,SURF4,SUSD3,S100A8,S100A12,S100B,TAGLN,TBC1D22B,TEFM,THRAP3,TIRAP,TLE3,TLR4,TOP1,TOP1MT,TOPORS,TPBG,TPR,TRA2A,TRA2B,TRIM25,TRIM55,TTC4,TTC39A,TUBA1A,TUBB,U2AF1,U2AF2,UNK,USF2,UTP14A,YWHAE,ZC3H18,ZIC1,ZNF207,ZNF326,ZNF598,ZNRD2,ZSCAN20*
***SHH***	Sonic hedgehog protein (Q15465)	Hedgehog family	–	–	*BOC,CDON,DERL1,DERL2,DISP1,DISP2,EDEM1,EDEM2,EDEM3,GAS1,GLI1,GLI2,GLI3,GPC3,GPC5,HHAT,HHIP,PTCH1,PTCH2,SAP18,SEL1L,SIN3A,SMO,SUFU,SYVN1*
***SLC9A1***	Sodium/hydrogen exchanger 1 (P19634)	Monovalent cation:proton antiporter 1, transporter family	*CALM1,CALM2,CALM3,CDH1,CHP1,DAXX*	–	*AKT1,AOC3,AP4S1,ARRB1,CA2,CCDC107,CCR6,CD44,CHP2,COA3,EGF,EGFR,ENO1,EZR,FAM189A2,FNDC5,FUT1,GALNT6,GALNT7,GBF1,HMMR,HRAS,HSP90AA1,HSPA4,HYAL2,IPPK,KRAS,LPAR1,LRRC55,LYPD3,LYVE1,MAD2L1,MAPK1,MAPK3,MAPK14,MAP3K7,MAP3K14,MAP4K4,MDFI,MSN,NEDD4,NRAS,PTGER3,PTGIR,RAB5C,RDX,RHOV,ROCK1,RPS6KA6,SEMA7A,SLCO6A1,SLC20A1,SLC22A2,STAB2,TAF15,TCTN2,TCTN3,TESC,TMEM17,TMEM106B,TNFRSF1A,UBE2I,WNK1,YWHAB,YWHAZ*
***SMARCA4***	SWI/SNF-related matrix-associated actin-dependent regulator of chromatin subfamily A member 4 (P51532)	SNF2/RAD54 helicase family	*CTNNB1,HMGB1,SS18L1*	*ARID1B,CTNNB1,CUL3,FMR1,SMARCA2,TOP2B*	*ACTB,ACTC1,ACTG1,ACTL6A,ACTL6B,ADD1,ADNP,AHR,ANGPTL4,ARID1A,ARID2,ASF1A,ATF3,ATL2,BACH1,BAZ1B,BCL6,BCL7A,BCL7B,BCL7C,BICRA,BICRAL,BMI1,BRCA1,BRD4,BRD7,BRD9,BRWD1,BYSL,CABIN1,CAD,CAND1,CARM1,CBX1,CBX3,CBX5,CCNC,CCNE1,CDC5L,CDK8,CDK19,CDKN2A,CDX2,CDYL,CEBPA,CEBPB,CHAF1A,CHD3,CHD4,CHD7,CHEK1,CHFR,CHMP5,CIITA,COPS5,CPSF2,CREB1,CREBBP,CSNK1A1,CSNK1D,CSNK1E,CSNK2A1,CSRP2,CUL1,CUL4B,DBP,DDIT3,DDX5,DNMT3A,DPF1,DPF2,DPF3,EED,EFTUD2,EPAS1,EP300,EP400,ERCC3,ESRRB,ESR1,ESR2,ETS2,EWSR1,EYA1,EZH2,E2F1,E2F4,E2F6,FANCA,FANCD2,FBXO6,FBXW7,FLI1,FOXA2,FOXE1,FOXO1,FOXP3,FUS,FXR1,GABARAPL2,GATAD2B,GATA1,GATA4,GLI1,GMNN,GTF2B,GTF2F1,GTF2H3,GTF3C3,HDAC1,HDAC2,HDAC3,HDAC9,HIF1A,HIF1AN,HIRA,HIST2H2BE,HIST2H3C,HIST3H3,HNRNPC,HNRNPU,HSF1,HSF4,HSP90B1,HSPB1,H2AX,H2AZ1,H3C1,H3C6,H4C6,IKBKE,IKZF1,IKZF2,IKZF3,ING1,IRF1,JUN,JUNB,KAT2B,KDM1A,KDM4A,KDM4B,KDM4C,KDM5C,KDM6A,KDM6B,KIAA1429,KLF1,KLF4,KPNA1,KPNA2,LMNB1,L3MBTL2,MACROH2A1,MAML1,MARK4,MBD2,MBD3,MCPH1,MDC1,MDM2,MECP2,MED6,MED17,MED21,MEF2A,MLLT1,MPHOSPH6,MPP6,MRTFA,MTA2,MYBBP1A,MYC,MYEF2,MYO18A,MYOCD,MYOD1,MYOG,MYSM1,NANOG,NCL,NCOA1,NCOA3,NCOR1,NCOR2,NEIL3,NEUROD1,NEUROG2,NF1,NFATC1,NFATC2,NFE2L2,NFKB2,NKX2-1,NKX2-5,NONO,NOP56,NOTCH1,NPM1,NR1H2,NR1H4,NR2C1,NR2C2,NR2E1,NR3C1,NR4A2,NSD2,NSD3,NTRK1,NUDCD1,NUMA1,OBSL1,OLIG2,PABPN1,PAG1,PARD6A,PARP1,PAX5,PAX6,PBRM1,PBX1,PCDH1,PELO,PHB,PHF10,PHIP,PIK3R1,PLCB1,PML,PMS2,POLR2A,POLR3A,POLR3C,POLR3F,POU5F1,PPARG,PRMT5,PRMT7,PSMB4,PSME3,PTGDR,PYHIN1,RAB5C,RASSF1,RBBP4,RBBP7,RBL1,RBL2,RBPJ,RB1,RCOR1,RCOR2,REEP5,RELB,REST,RNF2,ROCK1,RPS6KA5,RUNX1,RXRA,SALL4,SAP1 8,SAP30,SCYL1,SETD7,SFPQ,SF3A1,SF3B3,SIN3A,SIN3B,SIRT7,SIX1,SMAD2,SMAD3,SMAD4,SMARCAD1,SMARCA1,SMARCB1,SMARCC1,SMARCC2,SMARCD1,SMARCD2,SMARCD3,SMARCE1,SMC3,SNIP1,SOX2,SOX4,SP1,SPTA1,SRCAP,SRF,SRGAP3,SRRM2,SSX1,SSX2,SS18,STAT1,STAT2,STAT3,STAT6,STAT5A,STK11,SUPT16H,SUZ12,TAF9,TAF10,TAF15,TBP,TBX5,TBX20,TBX21,TCF3,TENT4B,TERT,TET2,TFCP2L1,TOP2A,TOPBP1,TOR1AIP1,TP53,TRIM25,TRIM28,TRIM33,UBE2O,UBN1,UPF1,USF1,VAPA,VCAM1,VDR,WDR77,WWOX,YAP1,YWHAZ,YY1,ZBTB7A,ZEB1,ZMIZ1,ZMIZ2,ZMYND8*
***SNAP25***	Synaptosomal-associated 25 kDa protein (P60880)	SNAP-25 family	*CADPS,CDH1,DLG4,DNAJC5,KCNB1,LRRK2,PRRT2,RPH3A,SNAPIN,SRCIN1,STX1A,SYT1,VAMP2*	*APP,CPLX1,CPLX2,DLG4,GRIN2B,MAP6,SYN3*	*ADCY6,APBA1,AP2A1,AP2B1,ATP1A1,ATP6V0D1,CENPF,CLSTN1,CLTC,CPLX3,CPLX4DCTN2,DGUOK,DNAAF2,DNM1,EGFR,FBXO7,GAPDH,GLP1R,GOSR1,HGS,HSPA8,HSPG2,HTT,ITM2B,ITSN1,KIF5B,KIF5C,MTNR1A,MTNR1B,NAPA,NAPB,NCKAP1,NSF,NUFIP1,PACS1,PPP1R9B,RAB3A,RAD23B,SCAMP1,SGTA,SLC14A2,SNCA,STXBP1,STXBP5,STX1B,STX2,STX3,STX4,STX5,STX6,STX11,STX12,STXBP1,SYP,SYT2,SYT3,TRIM9,TUBA1A,TUBA4A,TUBB,TUBB2A,UBC,UNC13B,UQCC2,VAMP1,VAMP3,VAMP4,VAMP5,VAMP7,VAMP8,WASHC3,YWHAB,YWHAE,YWHAZ,ZDHHC17,ZFHX3,ZNF177*
***SNAPIN***	Synaptosomal-associated protein 25-binding protein (O95295)	SNAPIN family	*LRRK2,SNAP25,VAMP2*	*HERC2*	*ABI2,ACTN2,ADCY5,ADCY6,AP3B1,AP3B2,AP3D1,AP3M1,AP3M2,AP3S1,AP3S2,ATP1A1,BCAS4,BFSP1,BFSP2,BIN1,BLOC1S1,BLOC1S2,BLOC1S3,BLOC1S4,BLOC1S5,BLOC1S6,BNIPL,BORCS5,BORCS6,BORCS7,BORCS8,CCDC102B,CCDC151,CDC42BPA,CEBPG,CEP170,CEP170P1,CMYA5,CNGA4,COG6,COG7,COPS4,CSNK1A1,CSNK1D,CSNK1E,C20orf202,DDR1,DES,DNM2,DOCK9,DST,DTNBP1,DYSF,EEF1G,EGLN3,ELP1,ENO3,ENTHD1,EXOC1,EXOC7,FAM114A1,FSD2,GPRASP1,HAP1,HPS3,HPS5,HPS6,IKBIP,IMMT,INSYN1,KANK2,KAT5,KAT7,KIF5C,KLC1,KPNB1,KRT15,KRT16,KRT17,KRT19,KRT20,KRT24,KRT27,KXD1,LAMA2,LAMC1,LAMTOR1,LAMTOR5,LMNB1,LRP12,MACF1,MAN2C1,MAPK14,MRPS14,MTNR1A,MYH3,MYH7,MYH14,MYPOP,NDC80,NECAB2,NME7,NUP62,NUP62CL,OCRL,PCYT1A,PLAC9,PLEC,RABEP1,RABGEF1,RAB9A,RETREG1,RGS7,RNF13,SCLT1,SCOC,SLC2A4,SLC14A2,SMAD2,SMG1,SNAP23,SPAG5,SPP1,SPTB,SPTBN1,STX4,ST7,SYBU,TCP10L,TEX35,TFIP11,TFRC,TGOLN2,TMEM171,TOR1A,TOMM70,TPM1,TPM2,TPM4,TRAK2,TRIML2,TRIM63,TRIM69,TRPV1,TSKS,VAMP7,VAMP8,VOPP1,WASHC3,YWHAE,YWHAZ*
***SNCG***	Gamma-synuclein (O76070)	Synuclein family	*MYOC*	–	*ADRB2,AHCYL1,ATOH7,BMPR1A,BUB1B,CENPE,DDX5,DYNLL1,EXT2,FABP4,FAM78B,FAM90A16P,FUBP1,GLYR1,GRK1,GRK2,GRK5,GRK6,HNF4A,ILF3,JPT1,JPT2,KCNS3,MAPK1,MAPK3,MAPK8,MCM3,METTL14,MMRN2,MSN,NDNF,PFDN2,POT1,POU4F2,RBPMS,SLC6A4,SLC23A1,SNRPN,SULT1A1,SYNC,TERF1,TERF2IP,TIMP2,TUFM,UBA2,ZNRD2*
***SNTA1***	Syntrophin-1 (Q13424)	Syntrophin family	*ATP2B2,DAG1,SNTB2*		*ABCA1,ABCC4,ADAMTS6,ADGRB1,ADGRB2,ADGRB3,ADH5,ADRA1D,ADRA2A,ADRB1,AGRN,AGTR2,AKAP9,APOA1,ATP2B1,ATP2B4,ATP6V0B,CAV3,CD3E,CFAP69,COPB1,CTNNAL1,CYSLTR2,C3AR1,DGKZ,DMD,DTNA,DTNB,FABP1,FAM189B,FRMPD4,F8A1,GDA,GLS,GLS2,GNAT3,GOLGA2,GRB2,GUCY1A2,HMGA2,HMG20A,HTR2B,HTR2C,IL2RA,IL9,ITGB5,KCNA4,KCNA5,KCNJ4,KCNJ10,KCNJ12,KCNJ15,LMO1,MAGEE1,MAP4,MAPK12,MAS1,MCM7,MED8,MLC1,MTMR2,MYBPC3,NAT1,NDEL1,NEB,NMU,NOS1,PFN2,PLCB3,PLEKHA2,PPP1CA,PRLHR,PSKH1,PTGDR,RGS11,RPS6KA1,RYR2,SCN1A,SCN4A,SCN4B,SCN5A,SCRIB,SCTR,SGCA,SGCB,SGCD,SGCE,SGCG,SLC1A7,SLC2A3,SLC6A3,SLC16A7,SLC34A3,SNTB1,SNTG2,SPZ1,SSPN,SSTR1,SSTR2,STAM,TAGAP,TFAP2A,TGFA,TLX3,TNS2,TRA,TRBV12-3,TRPC1,UBE2V2,UTRN,WWTR1,XRCC6,YAP1,YWHAE*
***SNTB2***	Syntrophin-3 (Q13425)	Syntrophin family	*CPNE5,DAG1,DLG4,LIN7A,LIN7C,SNTA1*	*DLG4,DTNBP1,NDEL1,PTEN,XPO1*	*ABCA1,ADGRB1,ADRA1D,ANG,ARHGAP23,CASK,CAVIN1,CDKN1A,CFAP36,CFAP69,COG6,CTNNAL1,DGKZ,DMD,DTNA,DTNB,EGLN3,ELAVL1,ERBB4,ESR1,ESR2,EZR,FCGR1A,FLNB,G3BP1,GOLT1B,HMG20A,HSCB,KCNJ12,LRCH3,LURAP1L,MARK2,MAST1,MAST2,MCM7,PLEKHA2,PTPRN,PXDC1,RECQL4,RHPN1,RPP25L,SAV1,SCN5A,SCRIB,SGCA,SGCD,SGCE,SNTG1,SNTG2,SPAST,SPZ1,SSPN,SYNPO2L,SYT13,SYT15,TAGAP,TNS1,TNS2,TNS3,UBC,UTP4,UTRN,VCP,WDTC1,ZMYM1*
***SPHK1***	Sphingosine kinase 1 (Q9NYA1)	–	*MYO1C,MYO1D*	*HUWE1*	*AANAT,ACACA,ACER1,ACER2,ACER3,ACTB,ACTG1,AIF1L,ARMCX3,ARPC3,ARPC5L,ASAH1,ASAH2,BAG2,BECN1,BIRC3,CIT,CSRP1,CTSB,CYLD,DBN1,DCXR,DEGS1,DEGS2,DHPS,DPM1,DPP7,DSTN,EEF1A1,EP300,FHL2,FYN,GALK1,GMPS,GRB2,HNRNPA2B1,HP1BP3,HSPA8,LAD1,LLGL2,LTBP1,LYN,MAD2L1,MAP4K1,MARCKSL1,MBTPS1,MYH9,MYO1B,OAT,PCK2,PDZK1,PIK3CG,PLPP3,PPM1G,PPP2R2A,PRKCD,PRMT1,PSMA3,PSMA8,PSMB2,PSMB6,RAB35,RARG,RBM3,ROCK2,RPS6KC1,SGPL1,SGPP1,SGPP2,SPHKAP,SQSTM1,SRC,STAU1,SVIL,TAF4,TMOD3,TRAF2,TRAF6,TUFM*
***SRCIN1***	SNAP-25-interacting protein (Q9C0H9)	SRCIN1 family	*CDH1,SNAP25*	*DAO,SHANK3*	*AGAP2,BCAR1,BCAS4,CD2AP,CHSY3,CKAP5,CRK,CSK,CTTN,DMTN,ESR2,FBXO7,GIT1,GRID2,GRIP1,HNRNPL,ITM2B,LNX1,MAPRE1,MAPRE2,MAPRE3,NUFIP1,POLG2,PPP1R9B,SNCA,SORBS3,SRC,SYP,UBC,YWHAB,YWHAE*
***SS18L1***	Calcium-responsive transcription coactivator (O75177)	SS18 family	*SMARCA4*	*CUL3,DGCR6,HDAC4,SMARCA2,TCF7*	*AATF,ACTL6B,ANKRD22,ATF3,ATN1,BAG4,BCL7B,BCL7C,BICRA,BICRAL,BMI1,BRD1,CEP55,CREBBP,CSTF2,C1orf94,DPF1,DPF2,DPF3,ELF5,EP300,ESR2,FAM168A,GATAD1,HDAC2,HGS,LGALS3,MAPK1IP1L,MED30,MIA2,NAF1,NR1H3,NSD3,PAX8,PCGF6,PHF10,REC8,RFX6,RLIM,SF3B4,SKA3,SMAD1,SMAD3,SMARCB1,SMARCC1,SMARCC2,SMARCD1,SMARCD3,SMARCE1,SMC1B,SMC4,SNRPB,SNRPC,SSBP3,STAG3,STAT3,SYCP1,SYCP2,TAF9B,TFG,TNK1,TP53BP1,USP30,USP54,YWHAE,ZMIZ2,ZMYND19*
***STAC***	Src homology 3 and cysteine-rich domain-containing protein (Q99469)	Endophilin family	*LRRK2*	*APP*	*ANKLE2,CDCA4,CENPH,DNAJB13,FBXO11,FBXO25,FCHSD2,GOLGA2,HLA-G,KANK2,KIAA1958,KIR2DL1,KIR2DL4,KIR3DL1,KIR3DL2,KIR3DL3,LACTB2,LIMD1,LMNA,LZTS2,L3MBTL4,MEOX2,NCAPG2,NOTCH2NLA,NCKIPSD,NEDD4L,NEDD4,PARP2,PAX5,PAX6,PJA1,POU6F2,RNF208,SIRT1,SLCO5A1,SPART,STAC2,SULF1,TRIM45,TRIP6,TRMO,XKR9,YWHAB,YWHAE,YWHAZ*
***STMN2***	Superior cervical ganglion-10 protein (Q93045)	Stathmin family	*SNAP25,SYT4*	*NGFR,SESTD1,TRPC5*	*AHCYL1,ASRGL1,CCDC85A,CCHCR1,CEP70,CHD4,CHGA,CLSTN1,CTNNA3,DHRS2,DSTN,EEF1A1,FAM90A1,FANCC,FANCD2,GAP43,GATAD2A,GATAD2B,GCNT1,GNG3,GPRASP1,GPRASP2,IGBP1,MAPK8,MAPK10,MAPT,MBD2,MBD3,MTA1,MTA2,MTA3,NEFL,NMNAT2,OTUB1,PCDHGB1,PCDHGB2,PPM1G,PRKM8,PSMB1,PSMC1,RARB,RGS6,RGS20,SEC23A,SEC24A,SLC1A2,STAM,STMN1,STMN3,STUB1,SULT1A1,TAGLN3,TEX11,TFCP2,TUBB3,TXLNA,UBA2,UBE2I,VOPP1,WDYHV1,ZER1,ZNRD2*
***STX1A***	Synaptotagmin-associated 35 kDa protein (Q16623)	Syntaxin family	*CADPS,KCNB1,KCNMA1,LRRK2,RPH3A,SNAP25,SYT1,SYT7,UNC13A,VAMP2*	*CPLX1,CPLX2,DISC1,GRIN1,GRIN2B*	*AARD,ABI3,AGTRAP,AIG1,ANKRD46,AOC3,APBA1,APBA2,APOL2,APOL3,AQP3,ARL13B,ATP6V1B1,BET1,BLOC1S6,BNIP1,BTN2A2,CACNA1D,CASC4,CCSER2,CDC37,CDK5,CDR2,CD81,CFTR,CHGA,CLEC1A,CLN6,CLTC,CMTM7,CPLX3,CPLX4CYB5B,C4orf3,DDX49,DPP8,DGUOK,DNAAF2,EBAG9,EMD,ERG28,ETNK2,FAM3C,FAM9C,GIMAP1,GIMAP5,GOSR2,GPM6B,HAX1,HGS,HMOX1,HTT,ITM2B,JAGN1,KIFC3,MAL,MALL,MDM2,MIP,MMGT1,NAPA,NAPB,NAPG,NINJ2,NKG7,NRM,NSF,NUFIP1,OIP5,PGAP2,PLN,PLPP4,PLPP6,PLP1,PNLIPRP1,PPP1CA,PPP1CC,PSMA3,RAB3IL1,RMDN2,RNF4,RNF40,RTN1,RTP2,RUSC1,SCNN1A,SCNN1B,SCNN1G,SEC22B,SEPTIN1,SEPTIN5,SERP1,SERP2,SLC6A1,SLC6A2,SLC6A3,SLC6A4,SLC6A5,SLC6A9,SMIM1,SMIM3,SNAP23,SNAP29,SNAP47,SNCA,SNORC,STRIT1,STXBP1,STXBP2,STXBP3,STXBP5,STXBP6,STX1B,STX2,STX3,STX4,STX5,STX6,STX7,STX8,STX10,STX11,STX12,STX16,SUMO1P1,SYP,SYTL4,SYT2,TCEANC,TMEM14C,TMEM41A,TMEM60,TMEM100,TMEM120A,TMEM128,TMEM199,TMEM222,TMEM254,TNF,TNIP2,TRAF3IP3,TRDMT1,TSGA10IP,TSNARE1,TXLNA,TXLNB,UBC,UBE2I,UBTFL1,USE1,UNC13B,UPK1B,VAMP1,VAMP3,VAMP4,VAMP5,VAMP7,VAMP8,VAPB,VIM,VPS11,VPS16,VPS18,VSTM4,VTI1B,YWHAB,YWHAE,ZFPL1,ZNF12,ZNF136,ZNF250,ZNF440,ZNF441,ZNF490,ZNF479,ZNF557,ZNF696,ZNF707,ZNF785,ZNF835*
***SV2A***	Synaptic vesicle glycoprotein 2A (Q7L0J3)	Major facilitator superfamily	*CALM1,CALM2,CALM3,DLG4,LRRK2,SNAP25,SV2C,SYN1,SYT1,VAMP2*	*APP,DLG4*	*ADRB2,APBB1,AP2M1,ATXN1,BSN,CLEC2D,C9orf72,ETS1,FYN,GIT1,GP2,ITM2B,LAMA5,LGALS8,LTB4R2,MAGI2,NTRK1,PPP1R9B,RIN1,RNF4,SLC17A7,SV2B,SYNGR4,SYP,SYT2,UBC,UPK2,YWHAB,YWHAE*
***SV2C***	Synaptic vesicle glycoprotein 2C (Q496J9)	Major facilitator superfamily	*SNAP25,SV2A,SYT1*	*-*	*ABCC10,ABHD17B,EXTL3,GP2,LAMA5,LAMC2,MFSD5,MFSD8,MFSD10,MFSD11,NPLOC4,RHBDD1,SPNS1,SV2B,UBE4A,UFD1,UNC93A,VANGL2*
***SYN1***	Synapsin I (P17600)	Synapsin family	*CALM1,CALM2,CALM3,CAMK2A,DLG4,KCNMA1,LRRK2,SNAP25,STX1A,SYN2,SYT1,VAMP2*	*APP,CPLX1,CYFIP1,DAO,DLG4,NOS1AP,PRNP,SYNGAP1,SYN2*	*ACTB,AMPH,ANTXR1,AP2A2,AP3D1,ARMC1,ATG4A,BIN1,BLM,CASK,CRK,DDX5,DGCR8,DGKI,DGKZ,DGUOK,DNAAF2,DNM1,EGFR,ERC1,ERG,ERGIC1,ERI1,GRB2,GTF3C2,HAX1,HSPA8,HTT,ITM2B,ITSN2,KAT5,LUC7L3,MAPT,MAP2,MTNR1A,NCF1,NCKAP1,NOS1,NUFIP1,PFN2,PIK3R1,PPFIA3,PPP1R9B,PPP2CA,PRKACB,PRKN,PSEN1,PSMD2,RAB3A,RB1CC1,RIMS1,ROCK2,SAP130,SH3GL2,SH3GL3,SLC6A3,SNCA,SRC,SRRT,STXBP1,TSPOAP1,UBC,UNC13B,VIM,VPS54,YWHAB,YWHAE,YWHAZ*
***SYN2***	Synapsin II (Q92777)	Synapsin family	*CALM1,CALM2,CALM3,DLG4,HPCA,KCNMA1,SNAP25,STX1A,SYN1,SYT1,VAMP2*	*APP,CPLX1,CYFIP1,DLG4,NOS1AP,PRNP,SYN3*	*ACTB,ARMC1,CHD5,CHRM4,DGUOK,DNAAF2,DNM1,GRID2,HAX1,HSPA8,HTT,ITM2B,IQCB1,MED25,MTNR1A,NAPB,NCKAP1,NSF,PFN2,PLCG1,POLL,PPFIA3,PPP1R9B,PSMC3,RAB3A,RACK1,RIMS1,ROCK2,RPGRIP1L,SLC8A1,SNAP23,STX3,STX4,SYT2,UBC,UNC13B,VAMP4,VAMP8,YWHAB,YWHAE,YWHAZ*
***SYT1***	Synaptotagmin I (P21579)	Synaptotagmin family	*CACNA1A,CACNA1B,CALM1,CALM2,CALM3,DLG4,DNAJC5,LRP2,SNAPIN,SNAP25,STX1A,SV2A,SV2C,SYN1,SYN2,SYT4,SYT5,VAMP2*	*CPLX1,CPLX2,DLG4,FMR1,GRIN2B,PRNP,SCN2A,SYN2*	*APP,APH1A,AP1G1,AP2A2,ARMC1,BNIP2,BTN2A2,CACNB3,CACNB4,CASKIN1,CBFB,CDCA8,CLSTN1,CNKSR2,COPS4CSGALNACT2,CWC22,CYB5B,C6orf15,DGUOK,DNAAF2,EIF2B2,EIF2B3,EIF2B4,EIF2B5,EPB41L5,EPS15,FGF1,GIMAP5,GOLM1,HAX1,HMOX2,HTT,IKBKG,ITM2B,KCNA1,LRP1B,MFF,MIP,NAPB,NEDD4,NTRK1,NUFIP1,PCBD1,PGK2,PIP5K1C,PPP1R9B,PSENEN,PSEN1,RAB3A,RBM14,RD3,RRP7A,SCAMP5,SCN1A,SCN1B,SIN3A,SLC14A2,SLC17A7,SLC32A1,SMAD2,STON2,STXBP1,STX1B,STX2,STX3,STX4,SV2B,SYNCRIP,SYP,SYT2,SYT3,S100A13,TFCP2,TMEM14C,TMEM60,TMEM254,TSHR,UBC,UBIAD1,UNC13B,VLDLR,WNK1,YWHAB,YWHAE,YWHAZ,ZDHHC17,ZFPL1*
***SYT4***	Synaptotagmin IV (Q9H2B2)	Synaptotagmin family	*CACNA1B,NRXN1,SNAP25,STMN2,STX1A,SYN1,SYN2,SYT1,VAMP2*	*CPLX1,NRXN1,SYN2*	*CHGB,HNRNPC,MAPK8,MAPK9,MAPK10,NBR1,NDUFA12,NTRK1,OXT,PCSK2,PRKN,RAB3A,SGTA,SGTB,STX1B,STX3,STX6,SYNCRIP*
***SYT5***	Synaptotagmin V (O00445)	Synaptotagmin family	*CALM1,CALM2,CALM3,DNAJC5,ITPR1,SNAPIN,SNAP25,STX1A,SYT1,VAMP2*	*CACNA1D,CPLX1,HTR6*	*ATP6V1C2,CACNA2D2,CACNB3,CHGB,INS,ITPR3,PALM2,PCBP3,POLR2E,PRPSAP1,PSEN1,RAB1A,SNAP23,STXBP1,STX3,TOMM70,UBC*
***SYT7***	Synaptotagmin VII (O43581)	Synaptotagmin family	*CDH1,NRXN1,SNAP25,STX1A,VAMP2*	*CPLX1,NRXN1*	*ARNT,C2CD4C,EPS8L1,ESR1,FERMT2,HRAS,KRAS,MOV10,NPM1,NRXN2,NRXN3,SENP2,SIAH1,SNAP23,STX3,STX4,SUMO1,SYNCRIP,VAMP7,YWHAB*
***TNR***	Neural recognition molecule J1-160/180 (Q92752)	Tenascin family	*DLG4*	*DLG4,FMR1,HUWE1,PRNP,SHANK3*	*ACAN,AGAP2,BCAN,CCDC146,CNKSR2,FXR1,HOMER1,IL34,ITGAV,ITGA8,ITGA9,ITGB1,ITGB3,ITGB6,ITM2B,LGALS14,MAGEB4,MYC,NCAN,NCK2,PDE4DIP,PEX5L,POLR3C,PPP1R9B,PSMA1,PTPRZ1,RNF123,SDC4,TLR4,TNIK,TNN,TSC1,VCAN,WDYHV1,YWHAB,YWHAE,YWHAZ*
***TPM3***	Tropomyosin alpha-3 chain (P06753)	Tropomyosin family	*CTNNB1,LRRK2*	*CTNNB1,CUL3,DISC1,HDAC4,RPTOR,SHMT2*	*AAR2,ACTA1,ACTA2,ACTB,ACTC1,ACTG1,ACTG2,ACTN1,ACTN2,ACTN3,ACTN4,ADD1,AGR2,AHCYL1,AIF1L,ANLN,ANXA2,ANXA6,APBB1,APOE,AQP2,ATF2,ATP1A1,ATP5FPB,ATXN1,BCAR1,BCAR3,BLOC1S6,BRCA1,B9D1,CALCOCO1,CALML3,CAP1,CAPZA2,CAVIN3,CCDC102B,CCDC114,CCDC146,CCHCR1,CDC123,CDK2,CDK9,CFTR,CHRM3,CHUK,CKB,CKMT1A,CKMT1B,COQ2,COX4I1,COX5A,COX15,CSF1,CUL7,CYC1,C1orf216,DAPK1,DLD,DNM1L,DUX4,DVL2,EED,EFTUD2,EGFR,EIF1B,EIF4A2,EIF6,ELAVL1,ESR1,ESR2,FAM9C,FANCD2,FASN,FBXO5,FGA,FGF11,FKBP4,FKBP5,FN1,FTSJ1,GFM1,GPC1,GSN,HACE1,HDAC8,HDDC3,HIST2H2BE,HLA-B,HOOK2,HSF2,HSF4,HSP90AA1,HSP90AB1,HSPB1,IFIT3,IKBIP,IKBKE,IMP3,INVS,IQGAP1,ITGA5,ITM2B,JUN,KCNE1,KDM6B,KIFC3,KXD1,KYNU,LARS1,LCA5L,LURAP1,MAD1L1,MAGEA11,MAGED2,MAST2,MCC,MCM2,MCM3,MDC1,METTL2B,METTL14,MIB1,MIS12,MOV10,MTCH1,MYC,MYH7B,MYH9,MYH10,MYL3,MYL12B,MYO5C,MYO18A,MYO19,NDUFB10,NEK2,NOD2,NR2C2,NTRK1,NUP54,NXF1,OBSL1,OGA,OIP5,OSTF1,PAN2,PARK7,PAXIP1,PBX3,PDHA1,PDLIM7,PIH1D1,PLCB1,PPP1CB,PPP1CC,PRDX1,PRKAA1,PRKAB1,PRKN,PSMA2,PSMA6,PSMC5,RAD21,RIPK3,RNF2,RNF20,RNF181,RXRA,SCIN,SEC23IP,SEPTIN9,SF3A2,SGF29,SIPA1,SOAT1,SP1,SPAG9,SPTAN1,STMN1,SYCE1,TAB2,TBCD,TET2,TFPT,THAP1,THAP7,THOC1,TJAP1,TLK1,TMOD1,TMOD3,TMPO,TNF,TNNI1,TNNI2,TNNI3,TNNT1,TNNT2,TNNT3,TP53,TP73,TPM1,TPM2,TPM4,TRAP1,TRIM27,TRIP6,TSKS,TUBA1A,UBC,UQCRC2,VDAC1,VPS52,WASH3P,WDFY4,WTAP,YWHAB,YWHAE*
***TRPM7***	Long transient receptor potential channel 7 (Q96QT4)	Transient receptor family, LTrpC subfamily, TRPM7 sub-subfamily	*CTNNB1*	*ARID1B,CTNNB1,XPO1*	*ACTB,ACTBL2,ALYREF,ANXA1,APEX2,ARHGEF2,ARL15,BCL9,BUB1,BZW1,CAD,CANX,CCAR2,CCT2,CCT3,CCT4,CCT5,CCT6A,CCT7,CEP152,CORO1C,CUL4B,C1QBP,DDB1,DDX3X,DDX5,DDX6,DDX17,DNAH2,DNAH5,DNAH9,DNAH11,DSP,EED,EEF1A1,EIF4A1,EPPK1,EZH2,FLOT1,GTF2IRD1,G2E3,HDAC1,HIST3H3,HNRNPH1,HRAS,INO80,INO80B,ITPR2,JAK1,JUP,KAT6A,KDM4A,KIFC1,KLHL13,KNL1,KRAS,LAMB1,LAMP1,MARK2,MBD2,MBP,MLKL,MPP2,MYEF2,MYH2,MYH9,MYH10,MYH14,MYO9A,NEURL4,NKAIN1,NOD2,NOTCH2,NRAS,PHLDB3,PLCB1,PLCB2,PLCB3,PLCG1,PNLDC1,PRC1,PSCA,PTPRD,PTPRJ,PTP4A1,RAB7A,RANBP2,RING1,RNF2,RNF19A,RNPEP,ROCK1,RPL27,RTRAF,RUVBL1,RUVBL2,RYBP,SCYL1,SF1,SIRT1,SNAP29,SNRNP200,SNX27,SSB,STK10,TBC1D30,TCP1,TCTN3,TERT,TEX28,THSD7A,TIGD4,TIMELESS,TMED2,TOPBP1,TRERF1,TRIM3,TRIM25,TRPA1,TRPC1,TRPM2,TRPM6,TRPV2,TRPV4,TRPV5,TRPV6,TTC28,TTN,TUBA1A,TUBA1B,TUBA1C,TUBA4A,TUBB,TUBB2A,TUBB4B,UBE2V2,UBR1,USP24,VAPA,WDTC1,XRN2,YY1,ZACN*
***UNC13A***	Munc13-1 (Q9UPW8)	Unc-13 family	*CADPS,CALM1,SNAP25,STX1A,SYT1*	*CPLX1,DAO,FMR1,SHMT2*	*BSN,DNPEP,EPB41L3,ERC1,ERC2,FBXO45,MOV10,PEX5L,RAB3A,RIMS1,RIMS2,STXBP1,TNIK,UBXN10,UNC13B,UNC13C,YWHAB*
***VAMP2***	Synaptobrevin-2 (P63027)	Synaptobrevin family	*CALM1,CALM2,CALM3,DNAJC5,LRRK2,SNAP25,STX1A,SYT1*	*APP,CPLX2,HUWE1*	*ABCC2,ACAA2,ACAD9,AQP2,ARHGEF17,ARL13B,ASNA1,ASPSCR1,ATP1A1,ATP1A2,ATP1A3,ATP4A,ATP6V0A1,ATP6V0C,ATP6V0D1,ATP13A2,BAG6,BCAP31,BVES,CCDC155,CDC42,CENPF,CLSTN1,CPLX3,CPLX4CREB3L1,DDX60L,DGUOK,DNAJC13,DNM1,EBP,ERGIC3,EXOC3,FKBP2,FLOT1,FLOT2,GNAO1,GNB1,GNB3,GPM6A,HGS,HIBADH,HRAS,HSD17B4,HSPA8,IKBKB,ILF3,IQCB1,ITM2B,JAGN1,KRAS,LAMP1,LAMTOR3,LMNA,LPAR1,LYST,MAP1LC3B,MUC1,NAPA,NAPB,NCKIPSD,NRAS,NSF,NUFIP1,OSBPL2,PARK7,PICALM,PPM1H,PRDX5,PRKD3,RABAC1,RPS12,RTL8C,RTN1,S100A16,SCFD1,SEC22B,SEPTIN9,SF1,SFXN3,SLC7A14,SLC25A6,SLC30A3,SLC30A5,SLC30A8,SNAP23,SNAP29,SNAP47,SNCA,STXBP1,STXBP3,STX1B,STX2,STX3,STX4,STX7,STX8,STX11,STX12,STX19,SYP,SYPL1,SYT2,TAX1BP3,TCTN2,TDP1,TGOLN2,TIMM50,TMED10,TMEM17,TMEM216,TPCN2,TRAP1,UBA52,UBAP2L,UBE2M,UBXN1,UNC13B,USP7,VAMP3,VAMP8,VAPA,VAPB,VCP,WASHC1,WDFY1,WDFY2*
***VLDLR***	Very low-density lipoprotein receptor (P98155)	–	*PAFAH1B1,SYT1*	*PAFAH1B1,RELN*	*ANKRD36B,APOB,APOC1,APOE,CANX,CD36,CLU,CRKL,DAB1,EFNB3,FYN,KIAA1429,LPL,LRPAP1,LRRC4C,MYLIP,NR1H2,NR1H3,PAFAH1B2,PAFAH1B3,PCSK9,SEL1L,SH3KBP1,SNX17,YWHAE,ZNF408,ZSCAN12*
***WFS1***	Wolframin ER transmembrane glycoprotein (O76024)	Wofram syndrom or wolframin family	*-*	*APP,DRD2,KCNH2*	*ADIPOR1,AKT1,APLP2,ANTXR1,AQP6,ATF6,CD79A,CFTR,CKAP4,DNAJC3,EIF6,ESR2,FAM209A,F2RL1,GPC3,HSP90B1,IL6,ITGB3BP,KIAA1429,LAMC1,LTB4R2,LYPD3,MAPK6,MBTPS1,MIA3,PENK,PSMA5,P2RX2,P4HB,RNF4,SMURF1,STC2,SYVN1,TCTN3,TERF1,TPTE,TRARG1,UBE2L6,UPK1A,YIPF3*

Protein-protein interactions were based on data from human/mouse/rat homologous proteins. Some genes potentially related to severe mental illnesses (SMI) or other neuropsychiatric problems are summarized. Considering that schizophrenia and some other neuropsychiatric disorders such as autism may share common traits, we included some of the other neuropsychiatric disorders whose genes are the primary causes, and which are relatively mild-to-moderate. APC, PTEN, and TCF7 variants have been reported in autism spectrum disorder (ASD). CACNA1C variants have been reported in Timothy syndrome. CDKL5 variants have been reported in Rett syndrome and ASD. Both DISC1 and TCF4 variants have been reported in ASD, BD, and schizophrenia. DKK1, NEUROD2, and KREMEN1 variants have been reported in schizophrenia or schizoaffective disorder. FMR1 variants have been reported in Fragile X syndrome (FXS). SESTD1 variants have been reported in BD. TRPC6 variants have been reported in non-syndromic ASD. GFAP variants have been reported in Alexander disease (AxD). Other potential genes were identified in the UniProtKB protein annotation/knowledge database **Data Sheet 1**.

Following the transplantation of stem cells (such as several MSC subtypes) in different animal models of WM injury, increased MBP has been observed, demonstrating a potential for WM repair (36). Recent progress on modulating *in vivo* neuronal activity with non-invasive, high-precision, and high-resolution methods include the utilization of transcranial magnetic stimulation (TMS), optogenetics, chemogenetics, and optochemogenetics. TMS seems to be able to protect both the newly generated oligodendrocyte and the demyelinated neurons (37). Optogenetic stimulation of neurons in the premotor cortex in awake behaving mice has demonstrated a mitogenic response of NPC/OPC to neuronal activity for subsequent control of oligodendrogenesis and myelination within the deep layers of the premotor cortex (M2) and subcortical WM (38, 39). Ca^2+^/cation channel-optogenetics-based stimulation on transplanted neurons may show improved oligodendrogenesis and myelination upon the additional activity manipulations.

## Stem Cell Mechanisms of Neuropsychiatry and Non-Canonical Wnt

It is still difficult to mimic the severely progressed situations of human mental illnesses. Animal models have displayed attributes of neuropsychiatric problems after genetic/environmental inductions and/or systematic insults (inflammation, injury, pain, etc.). Stem cells carrying related gene mutations *in vitro* and *in vivo* can show particular disease phenotypes, such as decreased proliferation capacity, impaired migration and differentiation, abnormal neuronal/oligodendroglial maturation, abnormal neuronal activity/firing, and functional deficits (40–43).

Use of the patient-derived stem cells, such that the hiPSC are reprogrammed from patient fibroblasts, can help study NPC differentiation to a particular type of neuron and glia with potential neuropathological phenotypes (44). These have included hiPSC-differentiated: astrocytes and OPC with GFAP aggregate and deficient myelination phenotype for studying Alexander disease; types of neurons, NPC, or NPC spheroids with Rett syndrome mutations, major depressive disorders, or potential genetic variants from Dravet syndrome; α1 subunit of L-VOCC Ca_v1.2_ (CACNA1C variant) gain-of-function-related forebrain neurons for modeling Timothy syndrome; hippocampal neurons and NPC as an *in vitro* model of bipolar disorder; genetic deletions or duplications-caused or idiopathic schizophrenia models in almost all neural cell types. In other developmental disorders, NPC from Fragile X syndrome (FXS), for instance, showed augmented activity-dependent intracellular calcium responses and aberrant functional responses detected as early as in committed adult PSC-derived neuroblast stage (45). In their human PSC and mouse models, the role of L-VOCC was identified in NPC with migration, differentiation and fate determination defects. Neurons from FXS had been similarly affected by N- and P/Q-type Ca_v2_ channels and previously discovered by lost interactions with Fragile X mental retardation protein (FMRP, human gene name: FMR1).

Wnt/Ca^2+^ pathway. The Wnt-5a/Wnt-7a/Ca^2+^ signaling pathway shows neuroprotection. Activation of the Wnt/Ca^2+^ pathway includes increases in intracellular Ca^2+^ levels and Ca^2+^/calmodulin-dependent protein kinase II (CaMKII), protein kinase C (PKC) phosphorylation (e.g., at Ser579), and calcineurin dephosphorylation (e.g., at Ser637). CaMKII and PKC further activate some transcriptional factors (p-CREB and NFκB) and epigenetic regulators HDAC1/2/3, and calcineurin activates nuclear factor associated with T cells (NFAT). Wnt-5a binding to Frizzled 2 (FZD2)/FZD7 receptor also activates phospholipase C (PLC), which generates IP3 and binds to its receptor IP3R, releasing intracellular Ca^2+^ from the ER. Ca^2+^ transfer from endoplasmic reticulum excessive Ca^2+^-release into mitochondria can also lead to GTPase dynamin-related protein 1 (Drp1)-mediated mitochondrial fission through the activation of CaMKIα, PKCδ, and calcineurin complex. CaMKIα promotes p-Drp1 at Ser637, and PKCδ promotes p-Drp1 at Ser616. Wnt-5a promotes GABA_A_ receptor surface expression and recycling under activation of CaMKII. Wnt-5a/Ca^2+^ also shows an increase in nitric oxide (NO) production by nNOS in neuronal cells and inactivates voltage-gated K^+^ current through Wnt-5a-binding tyrosine kinase-like orphan receptor 2 (RoR2). Following the Wnt/Ca^2+^ signaling activation, Wnt-5a has shown neuroprotective effects, such as ameliorating induced neurotoxicity and synaptic loss through activated PKC. It may be synergistic with Wnt/β-catenin/GSK3β signaling (46, 47).

In addition to Wnt/β-catenin and Wnt/Ca^2+^ signaling pathways upon Wnts activation, the Wnt/planar cell polarity (PCP) pathway, controlling tissue polarity, migration, differentiation, and cell movement, involves many major signaling cascades downstream, both Ca^2+^-dependent and Ca^2+^-independent, such as c-Jun N-terminal kinase (JNK), nemo-like kinase (NLK), and RHOA (48). Human Wnt-5a/b and Wnt-11 are able to activate non-canonical Wnt/PCP signals through FZD3/6 receptors as well as some co-receptors, including ROR1/2 and PTK7. FZD3 variants have been reported in schizophrenia (49). Human Van Gogh homology protein VANGL1/2, Starry night homology protein CELSR1/2/3, Dishevelled homology protein DVL1/2/3, Prickle homology protein PRICKLE1/2, and Diego homology protein ANKRD6 can be activated by the PCP signaling. *PRICKLE1* and *PRICKLE2* variants may be involved in BD. Following Wnt/PCP signals, regulatory cascade mediators include Formin homology protein DAAM1/2, MAPKKK and MAPKK4/7, MAP3K7, or TAK1. Other molecules have included ANKRD6, GSK3B, and NKD1/2.

## Targeting Brain Pathology to Cognitive Impairments

Transplanted NPC/GPC, OPC, and immature neurons differentiated from types of PSC have been tested in various animal models with neuropsychiatric pathology and cognitive impairments or carrying mutations identified in human patients. Patient-derived differentiated cells and undifferentiated stem cells are invested models in neuropathological studies and drug screenings (50). It may be time to combine stem cell mechanisms and stem cell transplant with recent advances in MRI diagnosis and positron-emission tomography (PET) imaging and progress in computational neuroscience, potentially targeting brain pathology to cognitive impairments. Animals in the diseased states can show major pathophysiology in the cells, including demyelination, neuroinflammation, and cell/synaptic loss. Although there are many challenges to face, animal models for neuropsychiatric disorders and cognitive impairments have provided replicable myelin and oligodendrocyte function deficits. Animals are able to show some sorts of isolated behavior, latent inhibition, impaired attention, and working memory. On the other hand, the animals can show increased sensitivity to psychotomimetic drugs, increased psychomotor/locomotor agitation-like activity, cognitive deficits/depressive symptoms, loss of pre-pulse inhibition, and hyperactivity.

Transplantation of interneurons may facilitate host preceding neurons that can excite, inhibit, or stop the action of other neurons. Combined with an artificial activation on transplanted cells and transplant area, the connection between transplanted neurons and host neurons of high plasticity *via* synaptic formation may improve neuro-transmission, maintain balances between these cells and between excitatory and inhibitory signals, and help the individual function normally (i.e., cognition, learning and memory, etc.). For example, including a seizure treatment against excitation versus inhibition imbalance would be useful for treating neuropsychiatric disorders by means of decreasing excitation or increasing inhibition. Transplanted stem cell-derived neurons with high plasticity are more likely to form neuronal connections where they are needed most. Moreover, they provide intercellular communication/support and cell replacement. Encouragingly, inhibitory interneurons from the medial ganglionic eminences were transplanted into the brains of animals with epileptic seizures, and the animals showed increased inhibition and reduced seizures. There have been many important questions asked (refer to www.epilepsy.com) including but not limited to: How long would the effect of stem cells last? What side effects may happen? Could other problems that may occur in people with epilepsy (such as depression or anxiety) be helped by stem cells?

In a mouse ischemic stroke model, there was a significant decrease in myelination measured in the cortical tissue at several weeks following the ischemia. Mouse NPC transplantation increased the MBP level and the levels of synaptic proteins including synapsin-1 and PSD-95 in the peri-infarct regions (6). An elevation of intracellular Ca^2+^ initiated in the transplanted cells *via* a non-invasive intranasal drug-delivering route was able to further activate the differentiated cells, causing them to have even higher levels of Ca^2+^-dependent synaptic proteins. In a rat model of cranial irradiation-induced cognitive deficits, intrahippocampal transplantation of human NSC provided long-lasting cognitive restoration (51).

It may be concluded that modulating the Ca^2+^-dependent and activity-dependent synaptic plasticity has the potential to rescue abnormal neural/synaptic circuits in combination with a cellular therapy against progressed neuropsychiatric disorders and cognitive impairments. Enforced neuronal activity is also expected to promote oligodendrogenesis from OPC and adaptive myelination in the brain. Activity-dependent cell stimulation therapy may further enhance the cell-cell interactions and communications.

## Pluripotent Stem Cell-Derived Cell Therapy

There have been some great improvements in pluripotent stem cell-derived cell therapies. Cell applications have been used in recent preclinical studies for a certain group of patients or genetic models with mental disorders, for example, using inhibitory eNpHR3.0 optogenetic channel engineered mouse ESC differentiated cells for a schizophrenia model of the rat (52) and mouse ESC differentiated cells for post-TBI cognitive deficits in mice (53).

Regardless of the cell type, stem cells after transplantation promote their autocrine and paracrine protections, allowing cell-cell interactions and adaptive responses to the host environment as well as enriching the regenerative niches (54). Homing is one of the issues that are critically important for functional integration following transplantation (55, 56). Growth factors, chemokine, and extracellular matrix receptors that benefit homing and integration include but are not limited to the interleukin (IL)-8, monocyte chemo attractant protein 3 (MCP-3), stem cell factor (SCF), and stromal cell derived factor 1 (SDF-1).

Genetic stability. Studies have shown that human iPSC lines share numerous similarities in DNA damage response (DDR) with human ESC lines. The similarities can include G_2_/M cell cycle arrest, efficient double-strand break (DSB) repair, activation of checkpoint signaling such as p-ATM at Ser1981, p-CHEK2 at Thr68, p-NBS1 at Ser343, and p-TP53 at Ser15, and high levels of cell cycle arrest and DDR-related markers, including p-H3 at Ser10, RAD51, and γ-H2AX following exposure to γ-irradiation (57). Moreover, DDR pathways are widely involved during the cellular reprogramming processes in chromatin structure rearrangement and genetic/epigenetic regulations. Development for high-quality stable cell lines is being investigated. DDR and DNA repair response pathways can significantly contribute to the formation of variant and mutant sites during reprogramming and pluripotency maintenance at different stages of passage planning. When cells with SNV and detrimental mutations become differentiated, the quality of the cells and the phenotypes of differentiation/maturation are both affected. A standard operating procedure (SOP) with sets of step-by-step instructions must be developed to detect genetically unstable cells and cancer stem cells at every stage and before transplantation. To further eliminate the risk of tumorigenesis of transplanted cells giving rise to precancerous cells (although rare and cell source-dependent), quality control has been introduced based on the genetic properties of donor cells. One strategy can be planned during the iPSC banking combined with the applications of whole-genome sequencing. There have been some technological advances and corroboration proposals from human studies that allow the continuous monitoring of the exogenous cells and prevention of tumor formation. PET seems to be among the appropriate approaches for long-term *in vivo* cell tracking. The urgent safety concerns and future scientific needs also require the continuous control of transplanted cells. There may be several methods, at least for applied studies. For example, suicidal gene knock-in cells are ready for the elimination of potentially tumorigenic cells following transplantation.

Another concern is to reduce transplant rejection. The concern is that the cells, after transplantation, might trigger delayed transplant rejection. Universal donor cells or hypoimmunogenic cells, a promising solution, come from pluripotent PSC and differentiate into various cell types that are ready for being tested against a vast number of chronic and genetic diseases (58).

## Intranasal Delivery of Stem Cells

Intranasal administration has been considered as an efficient and non-invasive medication route to the brain or even for systemic administration to the CNS (59). The cells delivered within the nasal cavity have demonstrated the ability to migrate towards different brain regions, including the brainstem regions, cerebellum, cortex, hippocampus, olfactory bulb, and striatum, and even to the spinal cord (60). Moreover, the intranasal delivery of cells to targeted regions is less likely to generate ectopic tissue compared to systematic injections.

One of the well-tested migrating and regenerative mechanisms is SDF-1α/C-X-C chemokine receptor type 4 (CXCR4) axis involvement following intranasal delivery. SDF-1α (also named CXCL12) is produced by distinct types of adult cells and many tumor cells as a proliferation inducer, a cell death and survival regulator, and an angiogenic factor (61). Neural cells in the injury regions showed higher levels of SDF-1α. One of the best-known functions of the SDF-1α/CXCR4 axis is the regulation of progenitor cell (such as EPC and NPC) trafficking during development and after injury, cell chemotaxis, and homing into injury sites. Endothelial and smooth muscular expressions of CXCR4 contribute to the angiogenesis and regrowth of vessels into injury sites. CXCR4 and MMP-2 have been promoted under Wnt-5a activation in migrating OPC, neuronal outgrowth and some invasive tumor cells.

There are some other systematic effects of the chemokinetic and chemotactic SDF-1α/CXCR4 axis, which include:

1. Maintenance of HSC quiescence and hematopoiesis in the bone marrow as well as embryonic myelopoiesis in the liver and the bone marrow.
2. CXCR4-deficient mice exhibiting impaired limb innervation, myogenesis, and neuromuscular development.
3. Impaired vascular endothelium and blood vessel formation, for example, in renal vasculature, showing ballooning glomerular tuft and disorganized patterning.

As a manipulation method of improving neuronal cell therapy, SDF-1α-overexpressed mouse iPSC-NPC cells had increased presynaptic proteins such as synapsin-1 and SNAP25. SDF-1 also promoted migration, differentiation, and maturation of oligodendrocytes through regulating CXCR4-positive OPC. In other cell types, an overexpression of SDF-1α could promote MSC osteogenic differentiation and osteogenesis *via* upregulated ALP, OCN, Runx2, and p-Smadl/5 and osteoblastic angiogenesis, evident from increased CD31-positive cells and density (62). Increased migration of MSC was mediated by SDF-1 upregulation and was downregulated by CXCR4 antagonist AMD3100 (C_28_H_54_N_8_ 8HCl) treatment. The SDF-1α-secreting MSC and exosomes extracted from the cultured SDF-1α-secreting MSC reduced many pro-inflammatory factors and increased SDF-1α and VEGF (63). In the spinal cord, SDF-1α-engineered MSC further stimulates axonal growth.

Ischemic conditioning and hypoxic adaptation were beneficial for cellular protection against severe ischemia through upregulating cell survival/anti-apoptotic genes and metabolic reprogramming processes (64, 65). Hypoxic preconditioning of stem cells *in vitro*/ex vivo dramatically activated HIF-1α downstream and regulated NF-κB signaling. There were enriched anti-inflammatory/anti-oxidative and regeneration-promotive factors in hypoxia-preconditioned cells and the microenvironment. Therefore, it has been proposed that preconditioning stem cell therapy should be translated to cell clinics (12, 59, 66). In addition, increased migrating and homing capacity were demonstrated in hypoxia-primed stem cells through mechanisms involving CXCR4 upregulation, which further corroborate that the cells introduced *via* nose-to-brain methods are delivered into the host brain (67). There have been some significant concerns: How efficiently will the transplanted stem cell bypass the blood-brain barrier to reach different brain function areas? How long can the transplanted stem cell survive, and what is the ultimate destination of the transplanted stem cell? Considering that the transplanted stem cell takes therapeutic effect through autocrine or paracrine, what is its advantage or benefit compared to a virus-vector engineered benign factor delivery system? Considering that the transplanted stem cell goes through differentiation into different neuronal/glial cell types to replace the damaged endogenous nerve cell, can the transplanted stem cell integrate into the endogenous nervous system?

Intracranial injections of iPSC-NPC into the post-traumatic rat brain, an example of stem cell translational therapy, showed alleviated neuropsychiatric and cognitive defects (68). The cells could be detected with fluorescent pre-labeling at two weeks after the intracranial delivery. The transplantation procedure had to be skillfully performed. In the MCAO ischemic rat model, ESCs were injected into multiple locations and relatively evenly distributed around the ischemic core across the scarring tissue. Cells are generally unable to survive long and are barely able form functional units if their delivery is inside the dead injury core/macrophagic cell clusters, though there has been recent evidence showing long-term surviving vessel and functional cells in the injury core (69, 70), indicating that transplanted cells might have a chance to integrate within the core. We believe that intranasal delivery of cells can overcome the barriers, where the cells are recruited towards the formation of regeneration areas instead of migrating into the harsh microenvironment.

Intranasal delivery of stem cells has been demonstrated to increase retention and improve neurological/psychiatric outcomes. It is expected to facilitate cell homing toward the CNS and to avoid some potential side effects caused by other methods such as intravascular administration. Since there are intranasally delivered peptides, small molecules, viruses, plasmid, and bacterial phages that demonstrate successive entry into the brain, we also expect treatment *via* the intranasal delivery of cells to the CNS to be adopted for translation to the clinic. It may benefit Parkinson's disease, malignant gliomas, and stroke, as well as SMI. In combination with hypoxic pretreatment, BMSC have been shown to increase CXCR4 and MMP-2/9 and to reach the ischemic cortex and be deposited outside of blood vessels as early as 1.5 hours after administration. Several clinical trials are ongoing for treating neurological diseases with the intranasal delivery of cells.

## Study of Psychiatry *via* Brain Imaging

Brain imaging or neuroimaging may provide substantive evidence of brain structural, functional, and neurochemical alterations in neuropsychiatric disorders. It utilizes various techniques (such as fMRI, CT, PET, MEG, and EEG) to directly or indirectly image the structure, cellular function, or pharmacology of the nerves and neuronal network. It is therefore expected that neuroimaging observations can be consistent with the pathological biomarkers (such as hypermethylation/hypomethylation of specific DNA regions) or progression changes in numerous neurodevelopmental and neurodegenerative models such as FXS and MDD. Diffusion MRI and fMRI, which non-invasively measure WM structural changes and neural activities, respectively, have been proven to be sensitive to pathophysiological interpretation of neurological and psychiatric problems (71–74). Several lines of evidence from psychotic disorders have suggested that there is neural activity misintegration across different brain regions and circuit disruption/dysregulation. Evaluation of potential treatments can be performed to target, e.g., integration of neural activity revealed in the large-scale grey matter (GM) neuronal networks or circuits (75) and abnormalities in the WM integrity and structure (76).

Diffusion MRI based on water molecular diffusion, referring to the random translational (Brownian) motion of molecules, can be examined *in vivo* using diffusion tensor imaging (DTI). The MRI signal decay when diffusion gradients are applied reflects the displacement distribution of water molecules. Because diffusion of water molecules is restricted by tissue components such as cell membranes or macromolecules, diffusion MRI provides unique information about the internal structure of brain tissue. In such experiments, a diffusion tensor is calculated, and this consists of the three eigenvectors of diffusion arbitrarily labeled λ_1_, λ_2_, and λ_3_ from largest to smallest. Diffusion MRI has already been widely applied in the diagnosis and treatment of numerous brain disorders, most importantly in ischemic stroke, where a fall in apparent diffusion coefficient [ADC; a.k.a. mean diffusivity (MD) = (λ_1_ + λ_2_ + λ_3_)/3] of water molecules is seen within hours of the ischemic event. In the WM, water molecular diffusion takes place along the fiber orientation direction [λ_//_or axial diffusivity (AD) = λ_1_] to a much greater extent than perpendicular to it [λ_⊥_ or radial diffusivity (RD) = (λ_2_ + λ_3_)/2]. This process can be measured using DTI, where fractional anisotropy (FA—derived from λ_1_, λ_2_, and λ_3_) reflects the coherence of diffusion. DTI can be used to demonstrate WM abnormalities from a variety of neurological and psychiatric diseases such as multiple sclerosis (characterized by the destruction of myelin sheath) and schizophrenia. Interestingly, FA reductions are actually not as commonly interpreted to reflect a loss of WM integrity; the exact nature of this loss seems barely determinable by DTI. Abnormality signals could arise from intra- or extracellular water and an exchange between the intra- and extracellular water compartments, making it difficult to deduce the biological source. Reduced FA is likely to reflect demyelination, fiber crossing, axonal swelling, or atrophy.

The primary form of fMRI uses the blood-oxygen-level dependent (BOLD) contrast. This technique relies on the fact that cerebral blood flow and neuronal activation are somewhat coupled together. When activated within an area of the brain, blood flow to that region may also increase and be activity-dependent, which then increases the ratio of oxygenated hemoglobin (Hb) and deoxygenated hemoglobin (dHb), since the dHb is more magnetic (paramagnetic) than Hb, which is virtually resistant to magnetism (diamagnetic). Therefore, the change of the ratio leads to an improved BOLD-fMRI signal. This improvement can be mapped to show neurons that are active at the same time (77). The observed fMRI alterations are not regionally specific but are more pronounced in the association cortex (prefrontal, parietal, and temporal) and subcortical (limbic, striatal) brain regions (78). The default mode network (DMN) is of particular interest in the fMRI studies of neuropsychiatry (75). The DMN is a set of brain areas that are preferentially active in the absence of goal-directed activity and deactivate during the performance of sensorimotor or cognitive tasks (79). The DMN areas, including the lateral parietal cortex, posterior cingulate cortex, precuneus, medial prefrontal cortex, and hippocampus, show synchronous fMRI activity patterns. Because these brain areas are implicated in “internally focused tasks”, the DMN is hypothesized to serve ongoing, or default, functions of the brain such as self-referential mental activity and autobiographic memory retrieval. Multiple fMRI studies have shown abnormal DMN activities in schizophrenia, BD, depression, and some others (78). We expect that it can be used for the evaluation of stem cell therapy, which holds the potential of cell replacement, forming connections with host neurons in these areas, and facilitating neurotransmission and normal functions in relevant networks.

WM abnormalities are critical to the conceptualization of neuropsychiatric disorders as dysconnection (abnormal connection) syndromes (76). DTI studies have provided strong evidence that WM integrity has great functional significance. For example, DTI measures are correlated with fMRI activities in healthy controls. Compared with controls, late-onset depression patients showed decreased FA and increased RD between the DMN and the cingulo-opercular network, as well as the thalamus, and decreased FA and RD of the fiber tracts were both significantly correlated with cognitive performance (80). In schizophrenia, FA reductions have been associated with specific clinical presentations such as passivity phenomena, auditory hallucinations, or positive symptoms more generally, cognitive functioning including working memory, episodic memory, executive function, verbal learning, and visuomotor performance (81). This body of literature suggests that WM integrity is highly relevant to specific domains of brain function and dysfunction. It may be possible that FA reductions and WM abnormalities following stem cell transplantation are improved.

## Summary

Promotion of oligodendrogenesis and myelination can lead to functional benefits and regeneration to combat abnormal neurodevelopment and neurodegeneration of SMI. Modulation of stem cells and physiological Ca^2+^-dependent/activated proteins will be tested in future pre-clinical models and translational studies. Wnt signaling may be critical regulators of these mechanisms in psychiatry and stem cell transplantation therapy for regeneration and repair. Intranasal delivery of hypoxic preconditioned stem cells may be the most appropriate method for psychotic disorders. Neuroimaging is useful in the pathophysiological study of SMI and the evaluation for treatments based on changes in neural activities and WM integrity. Higher-resolution precise imaging may further allow the development of stem cell treatment targeting specific brain regions.

## Author Contributions

YoZ and ZW designed the research. YiZ, JS, CH, and XS contributed unpublished analytic tools. JS, CH, GC, ZL, JL, and XS analyzed the data. YoZ, XS, and ZW wrote the first draft of the paper. HL, CH, GC, XG, YaZ, LW, and ZL edited the paper. YiZ, XS, and ZW wrote the paper.

## Conflict of Interest

The authors declare that the research was conducted in the absence of any commercial or financial relationships that could be construed as a potential conflict of interest.

## Supplementary Material

Data Sheet 1 This file contains a list of all the potentially interacted genes of our identified calcium-related proteins with traced manual corrections and how many times each gene presented (specifically for genes that are interacted with more than one from the identified calcium-related proteins). As the data is generated and summarized across various databases and considering that at least a total of 2-3% gene set differences between human and rodent, the manual corrections are inevitable.
